# Experimental species introductions influence fungal community succession through positive and negative effects on resident species

**DOI:** 10.1093/ismeco/ycaf166

**Published:** 2025-09-19

**Authors:** Sonja Saine, Tadashi Fukami, Reijo Penttilä, Brendan Furneaux, Otso Ovaskainen, Nerea Abrego

**Affiliations:** Department of Agricultural Sciences, P.O. Box 27, FI-00014 University of Helsinki, Helsinki, Finland; Departments of Biology and Earth System Science, Stanford University, Stanford, CA 94305-5020, United States; Natural Resources Institute Finland (Luke), Latokartanonkaari 9, FI-00790 Helsinki, Finland; Department of Biological and Environmental Science, P.O. Box 35, FI-40014 University of Jyväskylä, Jyväskylä, Finland; Department of Biological and Environmental Science, P.O. Box 35, FI-40014 University of Jyväskylä, Jyväskylä, Finland; Organismal and Evolutionary Biology Research Programme, Faculty of Biological and Environmental Sciences, P.O. Box 65, FI-00014 University of Helsinki, Helsinki, Finland; Department of Biological and Environmental Science, P.O. Box 35, FI-40014 University of Jyväskylä, Jyväskylä, Finland

**Keywords:** assembly history, colonization, community development, competition, deadwood, DNA metabarcoding, facilitation, inoculation, joint species distribution modelling, species interactions

## Abstract

Successional pathways of microbial communities are influenced by the complex interactive dynamics among the resident and immigrating species, along with the interactive feedback loops with their environment. Although studies on microbial communities have described patterns of microbial succession, quantitative evidence of how resident communities respond to immigrating species and how such relationships translate into successional changes remains limited, especially for species-rich communities under natural settings. Here, we carried out a field experiment to investigate how the identity of immigrating species influences the successional pathways of wood-inhabiting fungi. We simulated immigration through inoculations of nine selected wood-inhabiting fungal species and characterized resident fungal communities before and one and two years after the inoculations through DNA metabarcoding. The experiments included 275 naturally fallen and 185 artificially felled fresh logs of Norway spruce, with different log types hosting distinct initial resident communities of fungi and representing different abiotic conditions. While the resident community succession was mostly explained by the log-level abiotic characteristics, the identity of immigrating species also influenced the composition of resident communities, and consequently community succession. The immigrating species influenced resident species mostly negatively, suggesting competitive interactions to be important determinants of community succession. The responses of resident species to the immigrating species were phylogenetically correlated, suggesting that shared traits underlie species interactions in the species-rich wood-inhabiting fungal communities. This study advanced the understanding of community succession in species-rich natural systems by providing experimental evidence that the immigrating species influence community succession through the phylogenetically structured responses of resident species.

## Introduction

Species succession is a well-known and fundamental feature of microbial communities. Studies in the field of environmental microbiology have demonstrated how microbial communities are posed to fast-changing species replacement dynamics, which can have profound effects on ecosystem function [1–3]. Microbial community succession thus provides an ideal setting for studying how the interplay between deterministic (e.g. biotic and environmental filtering) and stochastic (ecological drift) processes modulate community assembly [4, 5]. However, the driving mechanisms behind microbial community succession dynamics remain a key question in microbial community ecology, especially for communities under natural settings [6, 7]. Successional dynamics of natural microbial communities are highly complex. This is due to their highly species-rich nature and rapid changes in their community composition, the intricate interactive dynamics among the resident and immigrating species, and the continuous modifications that the species exert to the environment [1–5]. Importantly, understanding how the resident communities respond to the immigrating species and how such relationships translate into successional changes is needed to gain a more mechanistic understanding on community dynamics.

One of the factors affecting community structure and successional dynamics is species immigration history [8]. This phenomenon, known as priority effects, takes place when the arrival order and timing of early-arriving species affect the outcomes of local species interactions, which ultimately can affect the pathways of community succession and consequently community function [9–11]. Due to stochastic variation in colonization order and timing [12, 13], priority effects often lead to unpredictable community dynamics [8, 14, 15]. Furthermore, priority effects do not only depend on the timing of species arrival or the identity of interacting species, but also on the local abiotic conditions, which can alter the outcomes of species interactions [16–19].

Wood-inhabiting fungi provide an ideal study system to investigate how the interactions between the immigrating species and the resident communities influence community succession under natural settings. The local communities of wood-inhabiting fungi inhabit discrete pieces of deadwood, allowing manipulative treatments over a high number of experimental replicates [20]. Moreover, during the wood decay communities undergo succession [21–23] that involves strong interactions between the constituent species as they compete for space and nutrients [24, 25]. Priority effects have been documented to be important for wood-inhabiting fungal communities [10, 11, 22, 26–28], especially at the early stages of community succession [29, 30]. While capturing resources, wood-inhabiting fungi alter their abiotic environment, which can facilitate or prevent colonization of later arriving species and cause priority effects [26, 31, 32]. However, to the best of our understanding, no quantitative evidence exists on how the identity of immigrating species affects the interactions between arriving and resident wood-inhabiting fungal species and how this influences subsequent community succession. Interestingly, the responses of wood-inhabiting fungi to environmental factors have been found to be phylogenetically structured, suggesting that related wood inhabiting fungal species tend to share traits and hence, respond similarly to their environments [33–36]. However, whether community level responses to immigrating species with differing identities are phylogenetically structured remains unknown.

In this study, we investigated how the identity of immigrating species influenced the successional pathways of wood-inhabiting fungal communities under natural settings. For this, we carried out a field experiment including 275 naturally fallen and 185 artificially felled Norway spruce fresh logs hosting distinct initial resident communities [37] and representing different abiotic conditions [38]. In these logs, we artificially introduced nine wood-inhabiting fungal species via inoculations to simulate natural immigration events. To characterize past immigration history, we surveyed the initial resident fungal communities before the inoculations using DNA metabarcoding and then annually followed changes in community structure during succession for two years after the inoculations. To assess how the succession of resident fungal communities depended on the identity of inoculated species and the abiotic conditions, we used joint species distribution modelling as the main statistical tool. Specifically, we asked: (i) whether and to what extent immigration by an inoculated species affected the succession of resident fungal communities over the course of two years compared to the abiotic characteristics of the logs; (ii) whether these changes depended on the identity of immigrating species; (iii) whether the resident community responses to the inoculated species were phylogenetically structured, and (iv) whether the effects of immigrating species varied among the post-inoculation years or between the log types.

We expected that the arrival of new species would influence the succession of resident wood-inhabiting fungal communities. However, as different wood-inhabiting fungal species are known to exert different types of interactions [24, 25], we expected the type and strength of the influence to vary depending on the identity of both the inoculated and resident fungi. Furthermore, we hypothesized that the inoculated species would not influence the resident communities randomly across their phylogeny but that the community responses would be phylogenetically structured, reflecting the evolutionary origins of traits that determine species interactions. Finally, we expected the effects of immigrating species to vary across the years, being more prominent in the initial stages of colonization when species compete for establishment with the primary colonizers [39, 40]. We also expected the effects to differ between the log types, as natural and felled logs can provide different abiotic and biotic environments [37].

## Materials and methods

### Study sites and experimental design

Our study employed the same study sites and experimental design as in Saine *et al.* [37, 41]. In summary, we conducted the experiment at five forest sites dominated by Norway spruce (*Picea abies* [L.] Karst) in central and southern Finland (Supporting Information: Fig. S1). Each site covered two to five hectares (Supporting Information: Fig. S1), depending on the availability of suitable study logs. We selected both natural logs and living spruces with a minimum diameter of 20 cm at breast height (1.3 m). The selected natural logs included both broken and uprooted logs (50% and 50% of all natural logs, respectively), representing different mortality factors. The living spruces were selected among trees that had no visible signs of infection. To obtain felled logs, we cut living spruces using a chain saw in April–May 2019. All felled logs were in decay stage 1 (on a scale ranging from 1 to 5; [42]) and thus for comparability, we prioritized natural logs in decay stage 1, but if unavailable, we also selected logs in decay stage 2. Altogether, we selected 55 natural and 37 felled logs at each site, totaling 275 natural and 185 felled logs for the experiment. Among all natural logs, 65% were in decay stage 1 and 35% in decay stage 2. During the two-year experimental period from 2019 to 2021, all felled logs remained in decay stage 1, while 10% of natural logs progressed from decay stage 1 to decay stage 2. See Supporting Information: Table S1 for a site-level summary of study log characteristics.

### Fungal inoculations and DNA-based community surveys

For the fungal inoculation treatment, we included nine native spruce-associated wood-inhabiting fungal species, as in Saine *et al.* [41]: *Antrodia piceata, Antrodiella citrinella, Fomitopsis rosea, Perenniporia subacida, Physisporinus crocatus, Postia guttulata, Skeletocutis odora, Skeletocutis stellae,* and *Steccherinum collabens* (Supporting Information: Table S2; [43]). Each species was represented by multiple strains (Supporting Information: Table S2) originating from two to six locations in Finland (Supporting Information: Section S2.1) that we assumed to represent different genetic individuals. Fungal cultures were established from new source material collected from the field, and culture collections from the University of Helsinki were used as needed (Supporting Information: Section S2.1). The strains were grown in the laboratory on agar plates and species identifications were confirmed by Sanger-sequencing (Supporting Information: Section S2.2). Inoculation dowels were prepared by Kääpä Biotech Oy (Karjalohja, Finland). Mycelia were cut into pieces and placed in plastic growing bags together with oat grains. After one to two weeks, sterilized inoculation dowels made of industrial spruce timber (50 x 10 mm; Helsingin Erikoishöyläys Oy) were added to the bags, and the mycelia colonized the dowels in one to two months depending on the species.

In the field, we introduced the dowels into 10 inoculation points within each log. The points were positioned one meter apart on one side of the log, with the first point located one meter from the base. At every inoculation point, we drilled three holes towards the log center in a triangular pattern ~3 cm apart using a cordless drill (Makita, model DDF481) and a wood drill bit (11 x 105 mm) and placed two inoculation dowels into each hole. To prevent contamination, after each log we immersed the drill bit in 5% sodium hypochlorite (NaClO) for a minimum of 3 min, followed by rinsing in water and ethanol, and sealed the inoculation holes with gardening wax (Oy Neko Ab, Finland).

We inoculated all nine target species in five natural and three felled logs at every site, resulting in a total of 40 logs per species. The logs for each target species and the strain for each inoculation point were randomly chosen. Serving as controls, we additionally inoculated 10 natural and 10 felled logs per site with sterilized dowels. This resulted in 92 inoculated logs per site and 460 logs altogether. Prior to inoculations, we visually confirmed the absence of target species’ fruit-bodies on the logs. Furthermore, we conducted an 8-h survey in each study site and their immediate surroundings by two fungal experts, where natural occurrences of two out of the nine species were recorded: one occurrence in one site for *Antrodiella citrinella* and nine occurrences in three sites for *Fomitopsis rosea*. Due to the low number of natural occurrences, however, we proceeded with the planned inoculations.

**Figure 1:**
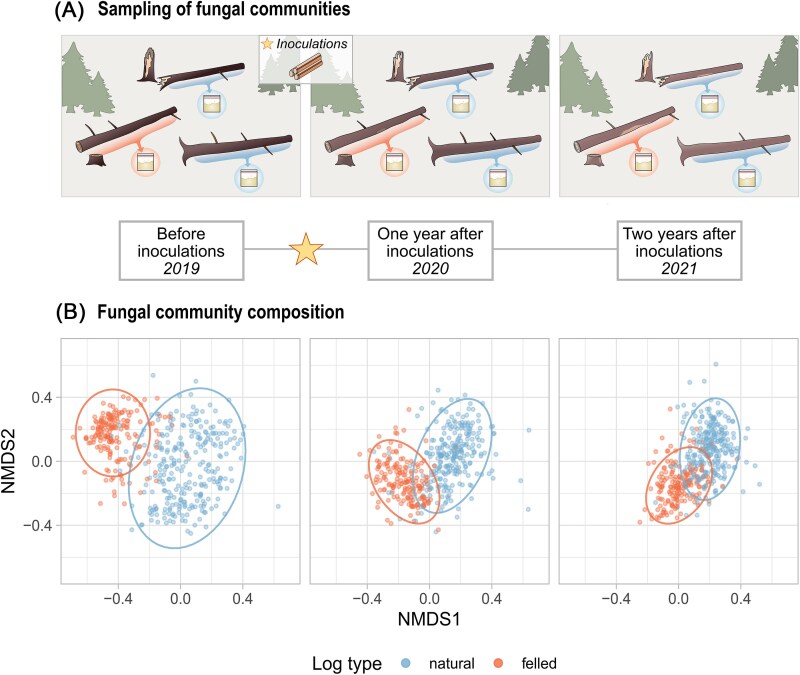
Illustration of (A) the study design where wood-inhabiting fungal communities were sampled during the three consecutive years, and (B) the fungal community composition for each corresponding year. (A) The experiment was implemented at five forest sites in southern and Central Finland. At each site, we selected a total of 92 Norway spruce logs, out of which 55 were naturally fallen fresh logs (broken or uprooted) and 37 were felled for the experiment. In 2019, we collected sawdust samples from each log prior to inoculations to describe the initial resident fungal communities with molecular analyses. Then, we inoculated each log with one out of the nine fungal target species using inoculation dowels (or with control dowels with no fungal mycelia). We collected corresponding sets of sawdust samples from each log one and two years after the inoculations in 2020 and 2021 to characterize the development of resident fungal communities. (B) Two-dimensional visualization of a four-dimensional NMDS ordination showing the change in resident community composition from before the inoculations (2019) to one (2020) and two years (2021) after the inoculations (stress = 0.165; *n*_2019_ = 451, *n*_2020_ = 435, *n*_2021_ = 453). Points represent logs as sampling units and point color shows the log type. Sampling units include both logs with fungal inoculations and controls. Ellipses encompass 95% of the sampling units for each log type. Other axis combinations are given in Supporting Information: Fig. S3.

From each inoculated log, we collected sawdust samples for the DNA-based survey of fungal communities. All logs underwent three rounds of sampling: one prior to inoculation to determine the initial resident community composition, and two post-inoculation to characterize the succession of resident communities (Fig. 1A). We conducted the first round of sampling at the same time as inoculation treatments in August–October 2019, and the second and third rounds in August–September 2020 and 2021, respectively. In the first year, we collected sawdust from the holes that were drilled for inoculation dowels. For the two subsequent years, we removed the bark, drilled holes ~2 cm away from each inoculation point and collected the resulting sawdust. We pooled samples from inoculation points at the log-level, and thus obtained one sample per log for each of the three years, for a total of 1380 samples.

### Sample preprocessing, DNA metabarcoding, and bioinformatic analyses

Procedures for sample preprocessing, DNA extraction, sequencing, and bioinformatic analyses followed the methodology described in Saine *et al.* [37]. We stored the samples at −20°C upon field sampling, after which we preprocessed them for DNA sequencing by freeze-drying and pulverizing. As a sequencing approach, we applied DNA metabarcoding using the ribosomal internal transcribed spacer. We additionally employed a spike-in approach to generate quantitative estimates on the sample-specific DNA amounts [44]. We used a development version of the OptimOTU pipeline for the bioinformatic analyses [45], including the following steps: filtering and trimming of raw sequence reads, denoising to generate amplicon sequence variants (ASVs), filtering and trimming of ASVs, probabilistic taxonomic identification using Protax-fungi [46], and the formation of operation taxonomic units (OTUs) based on taxonomically informed clustering. As a taxonomic unit for our statistical analyses, we employed OTUs that corresponded to clusters at species rank. The considered taxonomic classification were based on plausible identifications with a probability threshold of ≥50% [47]. See Supporting Information: Section S3 for a detailed description of each step.

### Statistical analyses

In our statistical analyses, we treated individual study logs as sampling units. The original community data consisted of 1380 sampling units (460 study logs sampled three times) and 4662 fungal OTUs. We excluded 11 sampling units with <10 000 reads, indicative of low sequencing quality, and removed classifications to the nine inoculated target species from all sampling units. The resulting community matrix included 1369 sampling units and 4646 OTUs.

We visualized the succession of resident fungal communities using NMDS (non-metric multidimensional scaling). For the ordination analyses, we excluded sampling units with less than five OTUs (*n* = 1339), applied the Hellinger transformation on the read count data, ran the analysis using the function metaMDS from the R package vegan [48] with four dimensions and Euclidean distances, and visualized the results using the R package ggplot2 [49].

As the main statistical tool for assessing the influence of inoculated species on the resident community succession, we fitted joint species distribution models using the Hierarchical Modelling of Species Communities framework with the R-package Hmsc [50, 51]. We fitted two joint species distribution models, one of which was based on OTU-level data and thus focused on OTU-level responses to the inoculations, and the other one on community-level data, focusing on responses at the community level. In the OTU-based model (henceforth called the presence-absence model), the response matrix consisted of the presence and absence of those 225 resident OTUs that occurred in at least 100 sampling units. Filtering the most commonly occurring OTUs was done to obtain sufficient data for estimating resident fungal responses to inoculated species at the OTU-level. In the community-level model (henceforth called the community facets model), we compressed the data matrix into six community facets. First, we included three response variables measuring OTU-richness, computed either for all resident OTUs (*n* = 4646), only for those commonly occurring OTUs, which were included in the presence-absence model (*n* = 225), or only for those rarely occurring OTUs which were excluded from the presence-absence model (*n* = 4421) (referred to as all, common, and rare OTUs, respectively). Second, we included the log-transformed fungal DNA amount. Third, we applied a model-based ordination with the R package gllvm [52] to presence-absence data on those resident OTUs occurring at least 10 times in the data, and used the two first latent variables as response variables characterizing community composition. The response matrix in the presence-absence model was modelled with a probit model, whereas the community facets model included a mixture of responses: OTU richness was modelled with log-normal Poisson, whereas DNA amount and community composition were modelled with linear models.

The explanatory parts of both models were the same. As environmental predictors, we included (i) log type (categorical predictor, natural versus felled), (ii) mortality factor of natural logs (categorical predictor, broken versus uprooted), (iii) decay stage (continuous predictor), (iv) sampling year (categorical predictor with levels 2019, 2020, and 2021), and (v) treatment (categorical predictor with 10 levels, corresponding to the nine inoculated species and the control). Since decay stage was measured only in 2019 and in 2021, we imputed the data for 2020 using the average. We included the treatment as its interaction with the years 2020 and 2021 to evaluate the year-specific effects of treatment on the post-inoculation years 2020 and 2021 since there is no treatment before the inoculation in year 2019. Note that both the inoculation treatment categories and the control category are part of the same covariate, but the control category is put as the intercept, i.e. the baseline category. In this way, any statistically supported result for any of the treatment species can be interpreted as a deviation from the control. We also included (vi) interaction between log type and sampling year to allow the average succession pathway to depend on log naturalness, and (vii) interaction between log type and treatment to ask whether the effects of immigrating species depended on the abiotic and biotic conditions associated with different log types. To control for the effect of sequencing depth, we also included in the model (viii) the log-transformed number of reads per sample (continuous predictor). To test the robustness of the results with respect to how sequencing depth was controlled for in the model, we also fitted an alternative version of the model, where we rarefied the community data to 10 000 sequences for each sample, and did not control for sequencing depth as a covariate. To account for the hierarchical spatiotemporal dependencies of the data (logs nested within sites, and each log sampled in three consecutive years), we included site (categorical predictor with five levels) and log (categorical predictors with 460 levels) as random effects. The log-level random effects accounted for the repeated replication in our experimental design. The presence–absence model included an additional hierarchical layer including a taxonomic tree of OTUs included in the model, which allowed us to estimate whether species responses to the predictors were phylogenetically structured [50]. For each taxonomic level, we assumed equal branch length in the taxonomic tree. We assessed the level of taxonomic signal in the species responses to the environmental predictors by estimating the parameter rho (ρ) and the posterior probability of its value being greater than 0 [53]. The rho parameter ranges from 0 to 1, with values closer to 1 indicating a higher taxonomic signal, i.e. more similar responses among related species. Additionally, we assessed which taxonomic groups showed systematic responses to the inoculated species. For this, we identified those groups of species which at the class-level had at least 10 OTUs of which at least 50% showed statistically supported responses of the same sign to a given introduced species.

We fitted the models with Hmsc-HPC [54], which is a high-performance computing (HPC) extension of the R-package Hmsc [51]. We sampled four MCMC (Markov chain Monte Carlo) chains with 3750 iterations, of which we dropped the first 1250 iterations as transient and thinned the remaining by 10, resulting in 250 posterior samples per chain and hence 1000 posterior samples in total. We evaluated MCMC convergence by examining the distribution of potential scale reduction factors [55] for the beta parameters that measure species responses to the included predictors (Supporting Information: Section S4).

We calculated the explanatory powers for the presence-absence model using Tjur’s *R*^2^, and for the community facets model using pseudo *R*^2^ for OTU richness and *R*^2^ for DNA amount and community composition. To quantify the relative contribution of each predictor included in the models, we applied a variance partitioning approach [51]. All statistical analyses were carried out with R version 4.3.2 [56].

## Results

### General patterns of resident community succession

We recorded a total of 4646 fungal OTUs in the resident communities, out of which 2343 were detected prior to inoculations, 3161 one year after the inoculations, and 3070 two years after the inoculations. The majority of the resident OTUs (57% of the OTUs and 63% of the sequence reads) were assigned to phylum *Ascomycota,* and a minority (38% of the OTUs and 36% of the sequence reads) to phylum *Basidiomycota*. Communities in natural logs were generally more species-rich than in felled logs: on average, communities in natural logs held 77 OTUs (range: 1–353), while communities in felled logs held 58 OTUs (range: 1–247) (Supporting Information: Fig. S2). The composition of resident communities changed during the two-year period after the inoculations, with their structure converging between natural and felled logs (Fig. 1B). Resident communities in felled logs experienced a greater average change in species composition (i.e. species turnover; Supporting Information: Fig. S4b), while the resident communities in natural logs increased more in species richness (Supporting Information: Fig. S2), thus increasing in nestedness (Supporting Information: Fig. S4a).

**Figure 2:**
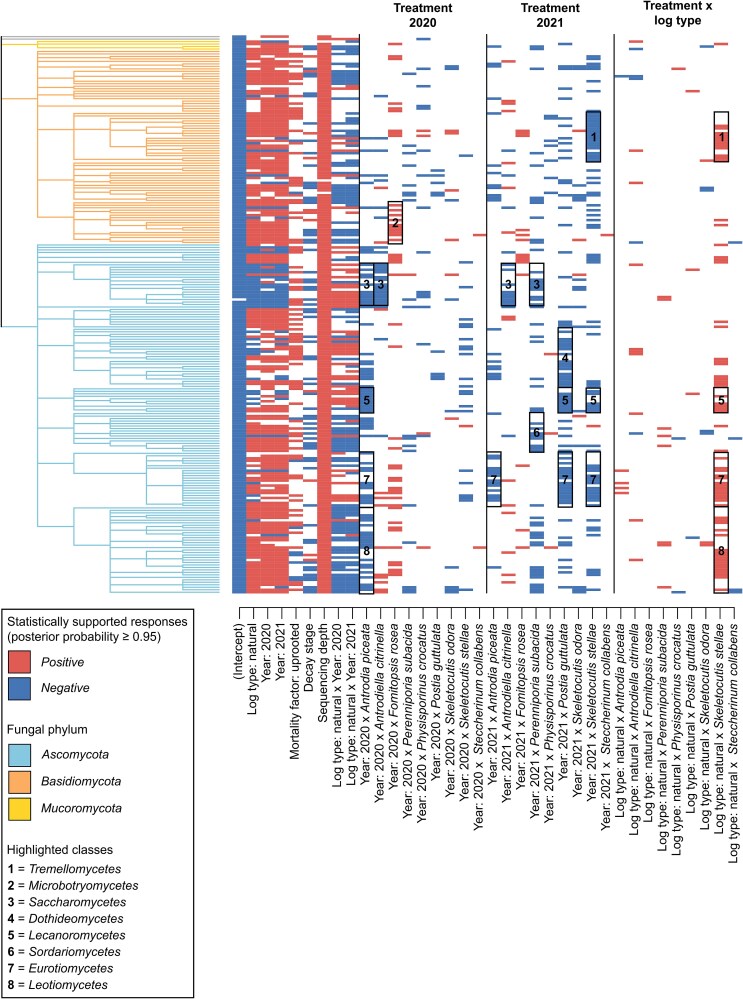
Posterior support for regression parameters (Beta) describing the responses of each OTU to the predictors in the presence-absence model. Plot shows those positive and negative responses to the model predictors that obtained at least 0.95 posterior probability. OTUs are displayed as rows, and they are ordered based on their taxonomic relationship. The rectangles highlight the fungal classes that show consistent responses, with the criteria that the class has at least 10 OTUs and at least 50% of them show statistically supported responses of the same sign. The taxonomical tree is colored by the fungal phyla. Taxonomic assignment for each OTU is provided in Supporting Information: Table S4. Posterior means for each regression parameter are provided in Supplementary Table S5.

The joint species distribution models confirmed the patterns observed in ordination analyses: sampling year influenced fungal communities in all models, suggesting that succession took place in the fungal communities during the experiment. The occurrence probability of most resident OTUs increased both one and two years after the inoculations, for some taxonomical groups more in the felled logs and for some other groups more in the natural logs (Fig. 2). Similarly, resident OTU richness (for all, common, and rare OTUs) and DNA amount increased during the post-inoculation years, the former more in natural logs and the latter more in felled logs (Supporting Information: Table S6). Likewise, community composition measured by the latent variables also changed over the years (Supporting Information: Table S6).

### Influence of abiotic and spatial predictors on resident communities

In both joint species distribution models, abiotic log characteristics captured most of the explained variance in the resident fungal communities across the years (Table 1). Among the measured log-level attributes, log type was the most important (Table 1). Since decay stage was rather homogeneous across the fresh experimental logs and changed little during the experiment, it explained only a small proportion of variation in the models (Table 1). Compared to log type and decay stage, the random effect of log explained a substantial amount of variation especially in resident OTU presence-absences, OTU richness, and DNA amount (Table 1), implying high relevance of nonmeasured abiotic and biotic log characteristics that had consistent effects over the study years. There was also site-specific variation in the resident fungal communities captured by the random effect of site in the models (Table 1). Finally, sequencing depth had a notable positive effect on observed OTU occurrences and community-level facets (Table 1; Fig. 2; Supporting Information: Table S6).

**Table 1:** Proportions of explained variance attributed to each model predictor in presence-absence and community facets models. The latter model included the response variables OTU richness, DNA amount, and community composition, for which results are shown separately in the table. For presence-absence, OTU richness, and community composition, the proportions of explained variance and explanatory powers are averaged across all OTUs, the richness of all, common, and rare OTUs, and the first and second latent variables, respectively. Predictors with the highest proportion of explained variance are bolded for each response variable.

	**Response variable**			
**Predictor**	*Presence-absence*	*OTU richness*	*DNA amount*	*Community composition*
*Log type*	14.2	4.3	7.8	**24.2**
*Year 2020*	8.5	5.1	3.3	11.1
*Year 2021*	13.9	3.5	7.4	19.5
*Mortality factor*	1.0	2.3	0.5	0.1
*Decay stage*	0.6	0.4	0.4	1.8
*Sequencing depth*	3.7	**24.0**	13.4	6.7
*Log type x Year 2020*	3.5	0.9	4.4	1.8
*Log type x Year 2021*	5.1	2.8	1.1	4.4
*Treatment x Year 2020*	0.9	4.5	2.1	0.6
*Treatment x Year 2021*	0.9	4.1	2.0	0.6
*Treatment x Log type*	1.1	6.9	2.8	1.0
*Random: log*	**35.9**	23.9	**47.6**	18.8
*Random: site*	10.6	17.4	7.0	9.5
*Explanatory power*	0.26 (Tjur R^2^)	0.32 (pseudo R^2^)	0.29 (R^2^)	0.69 (R^2^)

### Influence of immigrating species on the resident communities

Besides the abiotic and spatiotemporal characteristics, inoculated species had an additional effect on the resident communities. Although the effects of inoculated species were not large in terms of total variance explained (Table 1) and the level of community variation in the control and experimental logs was similar (Supporting Information: Figs S5–S6; Table S7), the identity of the arriving species altered the community succession in the treatment logs as compared to the succession in the control logs (Fig. 2). Resident OTUs showed predominantly negative responses to the inoculated species (Fig. 2), meaning that the occurrence probability of responsive OTUs was lower in logs inoculated with the target species than in the control logs. The negative effect of inoculations increased over the years: in 2021, the number of statistically supported negative effects was higher and the number of statistically supported positive effects was lower than in 2020 (Fig. 2). Our results showed that the inoculations affected resident OTUs consistently between natural and felled logs, with one notable exception: inoculations of *Skeletocutis stellae* had fewer negative effects in natural than felled logs (Fig. 2). This finding is however likely explained by the species’ poor colonization success, especially in natural logs (Supporting Information: Table S2).

Influence of the immigrating species depended strongly on its identity. Inoculated species with especially negative effects on the resident OTUs included *Antrodia piceata*, *Perenniporia subacida*, *Postia guttulata*, and *Skeletocutis stellae*, each affecting more than a fourth of all resident OTUs (Fig. 2). *Antrodia piceata* impacted more species one year after the inoculations, while *Perenniporia subacida*, *Postia guttulata*, and *Skeletocutis stellae* influenced the resident OTUs especially after two years (Fig. 2). In addition to the negative responses, some resident OTUs showed positive responses to the inoculations, i.e. they occurred more likely in logs where these target species were inoculated than in the control logs (Fig. 2). In particular, inoculations of *Fomitopsis rosea* had solely positive effects on the resident OTUs, especially in the first year after the inoculation, increasing the occurrence probability of one-fifth of the resident OTUs (Fig. 2). The results shown in Fig. 2 are robust with the method used for controlling for sequencing depth, as also the model based on rarefied sequencing data highlighted that species within a same class had consistent responses to the treatments (Supporting Information: Fig. S7). In line with OTU-level responses, the OTU richness and DNA amount showed consistent results (Supporting Information: Table S6; Section S5.1).

### Taxonomic signal in the resident community responses

Parameter rho, describing the strength of taxonomic signal in the resident OTU responses to the inoculations and other predictors, was high and statistically supported in the presence–absence model (ρ = .85, posterior probability >.99; Fig. 2). This finding indicated that more closely related OTUs showed more similar responses to model predictors than expected by random. Inoculations of *Antrodia piceata* affected more resident OTUs assigned to phylum *Ascomycota* than *Basidiomycota* particularly one year after the inoculations (Supporting Information: Table S8). Especially ascomycetous OTUs in classes *Eurotiomycetes, Lecanoromycetes*, *Leotiomycetes*, and *Saccharomycetes* were negatively affected by this species, with at least 50% of the assigned OTUs showing statistically supported responses (Fig. 2; Supporting Information: Table S8). This was also the case with the inoculations of *Postia guttulata*, affecting OTUs specifically in classes *Dothideomycetes, Eurotiomycetes*, and *Lecanoromycetes* (Fig. 2; Supporting Information: Table S8). In contrast, more *Basidiomycetes* were negatively affected by the inoculations of *Physisporinus crocatus* and *Skeletocutis stellae* than *Ascomycota* (Supporting Information: Table S8). Inoculations of *Fomitopsis rosea* positively affected especially OTUs in class *Microbotryomycetes* (*Basidiomycota*) *and Exobasidiomycetes (Basidiomycota)* and more than 40% of the OTUs in class *Eurotiomycetes* (*Ascomycota*) (Fig. 2; Supporting Information: Table S8)*. Antrodiella citrinella* had a negative effect especially on class *Saccharomycetes* (*Ascomycota*) (Fig. 2; Supporting Information: Table S8).

## Discussion

Community dynamics and species succession might not only result from the contemporary abiotic and biotic circumstances, but also from the history of community assembly, including past immigration events [8, 15]. However, quantifying the relevance of immigration events for natural species-rich communities has remained challenging, and consequently succession in natural communities has often been found highly unpredictable [8, 14, 15]. By simulating immigration through the inoculation of selected wood-inhabiting fungal species, in this study we demonstrated how the identity of immigrating species coupled with the initial abiotic environmental conditions influenced community succession, and that such changes were phylogenetically structured.

### Immigrations via inoculations shaped the fungal community succession

While the level of community variation in the control and experimental logs was similar, the identity of introduced species altered the community succession in the treatment logs as compared to the succession in the control logs. Inoculations affected different aspects of resident fungal communities, namely the species-level occurrence patterns, OTU richness, DNA amount, and community composition, mainly by negatively affecting the occurrence probabilities of resident species. We note, however, that because the community changes observed in our study were primarily driven by log-level characteristics, the inoculation treatment had only a relatively minor effect on the overall community variation. The type and strength of responses were influenced by the identity of inoculated species, while being generally consistent between log types with different characteristics. This result is in accordance with previous studies that have manipulated immigration order in wood-inhabiting fungal communities with a more limited set of initial colonizers both under field [11, 27, 28] and laboratory [10, 26] conditions, also showing that new species arrivals can change local community structure and successional pathways.

The effects of inoculated species depended on their identity, reflecting the importance of species immigration order and timing along community succession [8]. Across all inoculated species, the effects on resident communities were predominantly negative, although some positive effects were also recorded. Species with strong negative effects on the resident fungi decreased the occurrence probability of more than one in four OTUs, while also decreasing the resident community OTU richness and DNA amount. At the same time, one-fifth of the resident OTUs occurred more frequently in logs inoculated with *Fomitopsis rosea,* and logs inoculated with *Antrodiella citrinella* gained a higher OTU richness. These species-specific effects of inoculated fungi might reflect the different types of interspecific interactions that wood-inhabiting fungi are known to engage [24, 25, 57]. Although a majority of the described wood-inhabiting fungal interactions are competitive in nature [40, 58–60], facilitative interactions have also been reported [57]. As an example of the first case, inoculations of *Antrodia piceata* decreased occurrence probability of multiple resident OTUs as well as resident OTU richness and DNA amount. Establishment of *Antrodia piceata* may thus change the resident communities by inhibiting the growth of already occurring OTUs through competitive interactions. In fact, *Antrodia piceata* is a late-successional species, which generally are known to be stronger competitors [61]. *Fomitopsis rosea*, on the other hand, showed only positive effects on the resident species. However, elucidating whether such positive effects arise from direct facilitation or indirect effects (e.g. resource modification in ways that favor other species) remains untested from our study.

### Phylogenetically structured responses to immigrating species

The high taxonomic signal found in our study revealed that the more closely related the resident OTUs were, the more likely they were to respond in a similar way both to the inoculated species and the other predictors. Notably, the taxonomic signal was high in how the resident species responded to the inoculated species. Some taxonomic groups, even at the level of an entire class, showed consistent responses to immigration of a specific target species. This result suggests that the related OTUs share traits influencing their interactions with other species, as previously shown for example in plant communities [62, 63].

### Temporal dynamics

Considering how the effects of inoculations changed over time, the number of positive OTU-level responses to inoculations decreased from one to two years after the inoculations, while contrary to our expectations, we recorded more negative responses with time. When arriving to a community that is already occupied, a fungus must first compete for establishment and to quickly obtain resources [39, 40]. However, after such initial colonization phase, the fungus continues growing and gaining space. In this phase, competition with the co-inhabiting species may become stronger compared to the initial establishment phase. While our study only concentrated on the initial stages of wood decomposition, it is important to acknowledge that this process can persist for decades. Therefore, the data presented here cannot be used to evaluate community succession in the longer term, which can be an important consideration for understanding priority effects on wood decomposition. Likewise, we highlight that artificial species introductions differs from natural species colonization and may have stronger effects on resident communities than natural immigration events. There are two aspects that contribute to the higher establishment potential of artificially introduced species. Firstly, inoculated fungi are directly at a dikaryotic stage, which is known to have higher survival rates than the monokaryotic mycelium germinating from the spores [64]. Secondly, inoculum size, which is considerably larger in artificial inoculations than in natural colonizations, enhances establishment success of the inoculated fungi [61].

### Abiotic predictors of community succession

Compared to the species introductions, abiotic environmental factors explained the largest amount of variation in the resident communities. This was largely expected, since a large body of literature has shown that environmental conditions influence wood-inhabiting fungal communities especially at the log-level [20, 65]. In our study, log type, reflecting the naturalness of the logs, strongly affected fungal succession dynamics, and a majority of resident OTUs were more likely to occur in natural than in felled logs. The way a tree dies influences the associated fungal communities via variation in physiochemical deadwood properties [24, 38]. Differences in fungal community composition are especially pronounced between natural and artificially created deadwood habitats [37, 66–68]. Surprisingly, we found that decay stage, which is well known to increase fungal species richness from initial to intermediate decay stages [42, 69], had mostly negative effects on OTU occurrences. However, this results most likely relates to the fact that our sampling units predominantly belonged to decay stage 1 (75% of sampling units) and our analyses focused on frequently occurring species. Species that are more characteristic of decay stage 2 are thus less likely to be included in the analyses, potentially explaining the observed trend. Finally, across different models, a substantial part of the variation in fungal communities was explained by the random effect of log, meaning that some unmeasured factors at the log-level affected the communities consistently during the period of this study. These factors could include, e.g. abiotic environmental conditions such as local microclimatic conditions, dispersal limitations, or priority effects from earlier stages of colonization history than observed in this study.

## Conclusions

We carried out a manipulative field experiment with high level of replication to obtain still largely lacking and hence much needed empirical evidence on the factors shaping succession and priority effects in natural, species-rich communities. By simulating immigration through inoculations, we demonstrated how the identity of immigrating species affected resident community succession in a phylogenetically structured manner, providing conclusive evidence on the relevance of immigration events for microbial community succession under field conditions.

## Supplementary Material

SI_Saine_et_al_ycaf166

## Data Availability

Data and scripts used in this manuscript are available in Zenodo: 10.5281/zenodo.10959957.
