# Supplementary material for: Experimental species introductions influence fungal community succession through positive and negative effects on resident species
Source: ISME Commun. 2025 Sep 19;5(1):ycaf166. doi: 10.1093/ismeco/ycaf166 (PMC12510464; doi:10.1093/ismeco/ycaf166)
Supplement: SI_Saine_et_al_ycaf166 [file si_saine_et_al_ycaf166.pdf]

Supporting Information for

Experimental species introductions influence fungal community succession through positive and negative effects on resident species

Sonja Saine, Tadashi Fukami, Reijo Penttilä, Brendan Furneaux, Otso Ovaskainen & Nerea Abrego

Section S1. Study sites and logs

	1 Kesijärvi	2 Lapinjärvi	3 Luukki	4 Seitseminen	5 Sääjärvi
**Municipality**	Janakkala	Lapinjärvi	Espoo	Ylöjärvi	Janakkala
**Bioclimatic zone**	southern boreal	southern boreal	southern boreal	middle boreal	southern boreal
**Site type**	set-aside forest	set-aside forest	set-aside forest	national park	set-aside forest
**Site owner**	UPM	Metsähallitus	City of Helsinki	Metsähallitus	UPM
**Experimental area size (*ha*)**	2	4	5	4	5
**Mean stand age (*y*)**	58	64	89	83	72

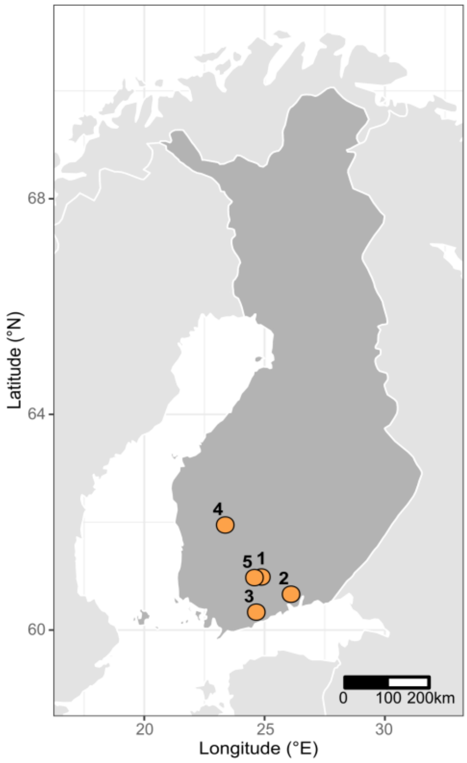

Figure S1. Table providing study site information and map of the study sites located in southern and central Finland. Sites were located in southern and middle boreal zones (Ahti *et al.* 1968), and they included set-aside forests owned by forestry company UPM, state owned set-aside forest and national park managed by Metsähallitus (State Forests, Finland), and a set-aside forest owned by the city of Helsinki. All sites were located in middle-aged or mature stands with a natural-like forest structure rich in deadwood and dominated by Norway spruce (*Picea abies* [L.] Karst), and their sizes varied from 2 to 5 hectares depending on the availability of spruce logs that fulfilled the selection criteria for study logs. Experimental area size was calculated as the area (ha) covered by the study logs with a 20-meter buffer surrounding each log. Mean stand age (y) was obtained by calculating average stand age for an area that covered all study logs with a 50-meter buffer. Data on stand age from Natural Resources Institute Finland (2019).

Table S1. Site-level summary of the study log characteristics. The experiment involved two log types: naturally fallen Norway spruce logs and felled Norway spruce logs that were created by cutting with a chainsaw. There were 55 and 37 natural logs in each site. Natural spruce logs had formed either by breakage or uprooting (*mortality factor*). As all felled logs were in decay stage 1 (on a scale from 1 to 5; Renvall 1995), we prioritized selecting fresh natural logs in decay stage 1. However, if natural logs in decay stage 1 were not available, we also selected natural logs in decay stage 2. Decay stage was measured in the beginning and end of the experiment in 2019 and 2021. Both natural logs and living spruces for the felled logs were selected with a diameter threshold of ≥ 20 cm at breast height (1.3 m from the base; *DBH*). In site Lapinjärvi, where large logs were scarcer, we also chose two natural logs with a DBH of 18 and 19 cm, and for felled logs, one spruce with a DBH of 18 cm and five with a DBH of 19 cm. Other measured log characteristics included ground contact and bark cover, measured as the proportion of inoculation area (from the log base up to 11 meters) touching the ground and covered by bark, respectively, and canopy openness measured as the proportion of open canopy above the inoculation area. In the table, decay stage 2019 and 2021 show the proportions of logs in decay stages 1 (DS1) and 2 (DS2) and with other variables, the first quantile, median, and the third quantile (Q1–median–Q3).

**Site**	**Log type**	**Mortality factor**	**n**	**Decay stage 2019**	**Decay stage 2021**	**DBH (*cm*)**	**Ground contact (%)**	**Bark cover (%)**	**Canopy openness (%)**
Kesijärvi	natural	broken	1	100/0%	100/0%	24.0	0	100	49.4
	natural	uprooted	54	56/44%	44/56%	23.0–27.0–29.0	0–10–20	90–90–100	31.2–39.8–53.1
	felled	-	37	100/0%	100/0%	27.0–29.0–32.0	10–20–40	90–100–100	20.8–23.6–30.2
Lapinjärvi	natural	broken	34	85/15%	79/21%	22.0–24.0–28.0	0–10–10	10–35–80	25.0–35.2–45.9
	natural	uprooted	21	86/14%	57/43%	27.0–29.0–36.0	0–10–10	70–80–90	24.5–29.4–38.7
	felled	-	37	100/0%	100/0%	20.0–24.0–26.0	0–10–10	40–80–90	23.5–28.5–31.9
Luukki	natural	broken	30	60/40%	50/50%	25.2–28.0–32.0	0–10–20	20–30–50	20.0–22.5–29.9
	natural	uprooted	25	36/64%	20/80%	32.0–36.0–39.0	0–0–10	10–20–40	20.6–22.6–28.3
	felled	-	37	100/0%	100/0%	27.0–30.0–33.0	0–10–30	30–40–70	19.5–22.6–24.6
Seitseminen	natural	broken	49	71/29%	63/37%	27.0–30.0–33.0	0–10–30	40–70–90	26.0–30.1–35.6
	natural	uprooted	6	67/33%	67/33%	29.2–31.5–33.8	0–5–25	75–90–97.5	29.7–35.2–36.3
	felled	-	37	100/0%	100/0%	25.0–28.0–31.0	10–30–40	90–100–100	23.8–26.4–29.8
Sääjärvi	natural	broken	23	78/22%	70/30%	22.0–24.0–26.0	5–10–20	5–20–50	17.2–32.8–40.4
	natural	uprooted	32	50/50%	47/53%	22.8–26.5–30.0	0–10–20	27.5–55–90	23.0–28.3–36.8
	felled	-	37	100/0%	100/0%	26.0–28.0–31.0	10–20–40	70–90–100	21.3–26.1–34.8

Section S2. Fungal inoculations

Table S2. Information on the inoculated fungal species, their ecology and colonization success. Each target species was represented by two to six strains each of which was obtained from different locations. The strains were obtained either from field collections or from the University of Helsinki fungal culture collections. All species use Norway spruce as their host tree species, and three of the species are brown-rot fungi and six white-rot fungi. All but one of the species are currently red-listed in Finland. Threatened species are marked with an asterisk. Colonization success shows the proportion of logs in which the inoculated target species occurred either one year (2020) or two years (2021) after the inoculations separately for natural and felled logs. Information on host tree species, rot type, and distribution follows Niemelä (2016), and the red-list statuses Kotiranta *et al.* (2019).

Species	Strains (n)	Host tree species in Finland	Rot type	Distribution in Finland	Red-list status	Colonization success 2020		Colonization success 2021	
						natural	*felled*	*natural*	*felled*
Antrodia piceata	3	spruce	brown	from southern Finland to Lapland	vulnerable*	12%	13%	12%	47%
Antrodiella citrinella	4	spruce, rarely birch, aspen, alder	white	the whole of Finland	near threatened	44%	60%	28%	47%
Fomitopsis rosea	5	spruce, rarely pine, kelo aspen	brown	the whole of Finland	near threatened	20%	40%	44%	67%
Perenniporia subacida	5	spruce, rarely pine, birch, aspen	white	the whole of Finland	near threatened	24%	60%	20%	93%
Physisporinus crocatus	2	birch, alder, coniferous trees	white	southern and south-eastern Finland	endangered*	8%	20%	4%	7%
Postia guttulata	4	spruce, pine	brown	up to the southern part of Northern boreal zone	least concern	44%	60%	32%	13%
Skeletocutis odora	6	spruce, aspen, rarely pine	white	the whole of Finland	near threatened	48%	80%	28%	80%
Skeletocutis stellae	3	spruce, pine	white	the whole of Finland	vulnerable*	4%	13%	0%	13%
Steccherinum collabens	5	spruce, rarely aspen	white	the whole of Finland	near threatened	52%	87%	24%	47%

Section S2.1. Source material for mycelial cultivations

We obtained the strains for mycelial cultivations by collecting new material in the field or by utilizing the University of Helsinki fungal culture collections (81 and 19% of the strains, respectively). Material for new fungal cultures were collected by sampling target species' populations in the field in August–November 2018. Altogether, the target fungi were sampled in 18 protected forest sites in southern and central Finland. The strains from the culture collection originated from two forest sites in central Finland and one forest site in southern Estonia.

For the new field collections, we used a non-destructive sampling protocol which depended on the target species. In the case of thin resupinate species, we taped a piece of a fruit-body on the lid of a malt extract agar plate (2%), closed and sealed the plate, and allowed agar gel to catch the spore deposition overnight. With thick resupinate and pileate species, we transferred a small piece of a fruit-body cut from the inside to an agar plate and sealed the plates.

Section S2.2. Cultivation of strains and Sanger-sequencing

The strains were cultivated and sequenced at the Natural Resources Institute Finland. The strains were grown in the laboratory at room temperature on malt extract agar plates until the mycelia fill the plates (90mm in diameter), taking from 11 to 74 days depending on the strain. Growth of the mycelia was visually checked on a regular basis and in the case of contamination, the uncontaminated mycelia was moved to new agar plates.

Each strain was Sanger-sequenced to confirm the species identifications. For DNA extraction, the strains were transferred to MOS agar plates. The isolates were sampled by scraping the fungus into a garnet bead tube, homogenized with a FastPrep homogenizer, and stored at -80°C. DNA extraction was done with the E.Z.N.A.® Forensic DNA Isolation Kit (WVR D3591-02). In the PCR, the ITS region was amplified using primers ITS1F (F) (CTTGGTCATTTAGAGGAAGTAA; Gardes and Bruns 1993) and ITS4 (R) (TCCTCCGCTTATTGATATGC; White *et al.* 1990). A 1 µl of 1:100 diluted DNA extract was added to a mixture of 12.5 µl Dreamtaq Green PCR Master Mix (2X), 0.125 µl of each primer (25 µM) and 11.25 µl of PCR grade water. The PCR cycles included 1) initial denaturation at 95°C for 3 minutes, 2) denaturation with 35 cycles at 95°C for 30 seconds, 3) 55°C for 30 seconds, 4) extension at 72 °C for 1 minute, and 5) final extension at 72 °C for 10 minutes. After the PCR, the amplicons were ran on 1% agarose gel and purified. The sequencing was done by Macrogen using primer ITS1F. After this, the sequences were trimmed and filtered using Geneious Prime software and taxonomic classification was performed using Unite and Nucleotide programs.

Section S3. Methods for sample pre-processing, DNA metabarcoding, and bioinformatic analyses

Section S3.1 Sample pre-processing

Before DNA sequencing, we pre-processed the sawdust samples at the University of Helsinki, Finland with an aim to pulverize the samples. In 2019, we collected individual sawdust samples for each of the ten inoculations points per log and stored these samples in freezer at -20°C in the laboratory. To obtain log-level samples, we pooled the ten samples per log by taking a sub-sample (ca. 2 ml) of each using sterile tweezers and combining them in a 50 ml tube for a ca. 20 ml pooled sample. After pooling, the samples were returned to freezer. In 2020 and 2021, we collected log-level samples already in the field by pooling the sawdust from all ten inoculation points within a log in one ziplock bag. In the laboratory, we took a 20 ml subset of each sample, transferred them into a 50 ml tube using a clean disposable teaspoon, and stored the samples in freezer at -20°C. Before pulverization, we freeze-dried the samples after the sample collection for a given year was completed. We sealed the frozen samples with parafilm, punched the parafilm with a needle, and freeze-dried them for 62–93 h at 0.57 mbar vacuum and 15°C self-temperature using a Christ GAMMA 2-16 LSC freeze dryer. After drying, the samples were pulverized with a Mixer Mill MM 400 (Retsch) homogenizer. We placed each sample in metallic grinding jar (25 or 50 ml) with sterile tweezers, added a mix of sterile metal beads (6 x 4 mm and 2 x 10 mm in diameter), ground the samples for five minutes at a frequency of 30 s-1, and repeated until the samples were fully pulverized. We placed a subset of pulverized sawdust in a 5 ml tube (for 2019 samples, a 2–4 ml subset, and for 2020 and 2021 samples, a 1 ml subset). After each sample, we rinsed the grinding jars with water, dried them, sprayed with a DNA/RNA decontamination solution (PDS-250, Biosan SIA), and wiped with clean paper towels. We sterilized the metal beads and tweezers by dry heating for four hours at 200°C.

Section S3.2 DNA extraction, PCR, and sequencing

In the workflow for DNA metabarcoding, the sample lysis, DNA extraction, and PCR amplification were carried out at the Canadian Centre for DNA Barcoding (CCDB), and the next generation sequencing of the indexed amplicon libraries was conducted at the Advanced Analysis Centre at the University of Guelph, Canada. In CCDB, the sets of samples collected in years 2019, 2020, and 2021 were registered under accessions CCDB-22-0650, CCDB-22-0651, and CCDB-22-0652, respectively. We sent each set of samples together with a plate record detailing the order of samples within a 96-well sample array. The 5 ml tubes were organized in tube racks to match the order specified in the corresponding plate record, and their exterior surfaces were sterilized with 70% ethanol. Prior to lysis, samples underwent a 2-minute centrifugation at 5000g. Depending on the sawdust volume, 2–4 ml of insect lysis buffer (ILB), supplemented with 1% polyvinylpyrrolidone (PVP) and 25 uL of Proteinase K (20 mg/ml) per 1 mL of buffer, was added to each sample. After a 2-minute centrifugation at 2000g, the samples were placed in a rack on a shaker at 100 RPM and subjected to a 2-hour incubation at 56°C, followed by an additional 2-hour incubation at 65°C.

DNA purification followed the protocol outlined in Ovaskainen et al. (2020) implemented for the Global Spore Sampling Project (GSSP). Lysates underwent centrifugation for 5 minutes at 2000g. Subsequently, using a single-channel pipette, 100 ul of the lysate was sub-sampled into a 1 ml deep-well plate (Eppendorf Cat No) and combined with 200 ul of 5M GuSCN Plant Binding buffer. The resulting 300 ul mixture was transferred onto a 96-well 1 um Glass Fiber (GF) plate (PALL) to facilitate DNA binding with the membrane, followed by a 5-minute centrifugation at 5000g. The first and second DNA washes involved centrifugation at 5000g for 2 minutes with 300 ul of 5M GuSCN buffer and then with 300 ul of Plant Protein Wash buffer. The final wash step was repeated twice with 600 ul of Wash buffer and a centrifugation at 5000g for 5 minutes, after which

the GF plate underwent a 30-minute incubation at 56°C. DNA elution from the dried membrane was achieved with 70 ul of 10mM TrisHCL pH 8.0 and a 5-minute centrifugation at 5000g.

PCR amplifications were conducted in a 96-well format using 10.5 ul of standard CCDB Platinum Taq Master Mix, with the extracted DNA used as template for amplification. A volume of 2 ul from each sample was transferred to a template-free and reaction-ready premade PCR 1 plate, containing primers ITS3-misN6 and ITS4-misN6 from Ovaskainen et al. (2020). For the spike-in approach, a mixture of nine synthetic controls at a concentration of 0.001 ng/ul was added to each sample. No DNA templates were added to wells H1 and H2 serving as negative controls. PCR 1 cycling conditions included an initial denaturation at 94°C for 2 minutes, followed by 40 cycles of 40 seconds denaturation at 94°C, 1 minute annealing at 51°C, and 1 minute extension at 72°C, concluding with a final extension at 72°C for 5 minutes. PCR 2 (indexing) employed the same mastermix but included fusion primers with standard i5 and i7 Illumina indices (N701, N702, N703, N704, N705, N706, N707, N710, and S502, S503, S505, S506, S507, S508, S510, S511, S513, S515, S516, S517). PCR 1 products were diluted 1:1, and 2 ul of the diluted product were used as the template in PCR 2. Cycling conditions for PCR 2 comprised an initial denaturation at 94°C for 2 minutes, followed by 20 cycles of 40 seconds denaturation at 94°C, 1 minute annealing at 60°C, and 1 minute extension at 72°C, concluding with a final extension at 72°C for 5 minutes. A Biomek FXP robot was used for the transfers of DNA templates and indexed primers.

Following the PCR step, the amplicons were visualized on precast agarose gel with a bufferless E-gel system (Invitrogen). Amplicons from individual wells were pooled without normalization, purified with AMPure beads, quantified using a Qubit 2.0 fluorometer, and their size was assessed using an Agilent Bioanalyzer with a high sensitivity kit. The subsequent sequencing process was executed on an Illumina MiSeq platform with PE2x300, adhering to the standard protocols by the manufacturer.

Section S3.3 Bioinformatic analyses

In our bioinformatics analyses, we employed a developmental version of the OptimOTU pipeline available at https://github.com/brendanf/deadwood_priority_effects. This pipeline was implemented in R version 4.2.2 (R Core Team 2022) using the targets workflow management package version 0.14.2 (Landau 2021). We used Cutadapt version 4.2 (Martin 2011) to trim and filter the raw sequence reads. Specifically, we truncated the paired-end fastq files at the first base with a quality score of ≤ 2 at both ends of R1, ≤ 2 for the 3' end of R1, and ≤ 10 for the 5' end of R2. Then, we removed the multiplexing indices at both ends from both R1 and R2, with the presence at the 3' end coded as optional. Reads with "N" bases or a length less than 100bp were discarded. Subsequent filtering was carried out using DADA2 version 1.26 (Callahan et al. 2016), excluding read pairs with R1 having more than 3 expected errors or R2 having more than 5 expected errors, as well as reads mapping to the PhiX genome. We applied the procedures outlined in the standard DADA2 ITS pipeline (Callahan 2020) for dereplication, denoising, merging, and chimera-checking of reads for each run. Identification of reads corresponding to the SynMock spike sequences was done using the -usearch_global command in VSEARCH version 2.22.1 (Rognes et al. 2016) with a pairwise identity threshold of 0.9. We then used the number of spike sequences per sample to estimate sample-specific DNA amount by calculating the proportion between non-spike and spike reads.

We employed Protax-Fungi (Abarenkov *et al.* 2018) to taxonomically identify the remaining Amplicon Sequence Variants (ASVs) using a 50% probability threshold (Somervuo *et al.* 2017). We applied taxonomically informed pseudo-single-linkage clustering in three phases to cluster the ASVs and to form operational taxonomic units (OTUs). First, we former reference cluster cores by joining ASVs that identified to the same taxon at the current rank. Second, we matched and joined unidentified sequences to the cluster

cores using the -usearch_global command in VSEARCH. To achieve approximately single linkage clusters, this phase was repeated until no new matches were found. We kept taxonomically distinct ASVs in separate clusters by not merging the cluster cores. Third, we used OptimOTU 0.6.4 (https://github.com/brendanf/optimotu) to single-linkage cluster the remaining ASVs, utilizing a sparse distance matrix based on global alignments calculated using the calc_distmx command in USEARCH version 11.0.667 (Edgar 2010). The optimal clustering threshold at each rank was determined through the hierarchical optimization technique developed by Dnabarcoder (Vu et al. 2020), with the same USEARCH + OptimOTU single-linkage clustering. Taxonomically identified fungal sequences from the GSSP (Ovaskainen et al. 2020) served as references for threshold optimization, as those sequences use the same primers and are thus based on the same amplicon region as our data. As Protax-Fungi does not identify non-fungi, we utilized the -usearch_global command in VSEARCH to find the closest match to each ASV sequence in the Unite sh_matching pipeline Sanger references dataset (Abarenkov 2022). We applied a minimum match identity of 0.8 to identify ASVs belonging to non-fungal groups and removed phylum-level clusters that contained more known non-fungi than known fungi. Taxonomically unidentified clusters at each rank were assigned unique placeholder names following the format "pseudo{rank}_NNNNN", such as "pseudofamily_00123".

Section S4. MCMC convergence

The joint species distributions models were successfully fitted to the data in terms of MCMC convergence: for the beta parameters in the presence-absence model (respectively, in the community facets model), the mean potential scale reduction factor was 1.01 (1.00), with 3rd quartile 1.01 (1.00) and maximal value 1.41 (1.02) (Table S3).

Table S3. Distribution of potential scale reduction factors (PSRFs) for the beta parameters measuring responses to the included model predictors in the presence-absence and community facets models. The table shows minimum, first quantile, median, mean, third quantile, and maximum values for the potential scale reduction factors.

	**Model**	
**Potential scale reduction factor**	*Presence-absence*	*Community facets*
Min	0.998	0.998
1st quantile	1.001	1.000
Median	1.004	1.001
Mean	1.007	1.002
3rd quantile	1.009	1.003
Max	1.407	1.018

Section S5. Supplementary results

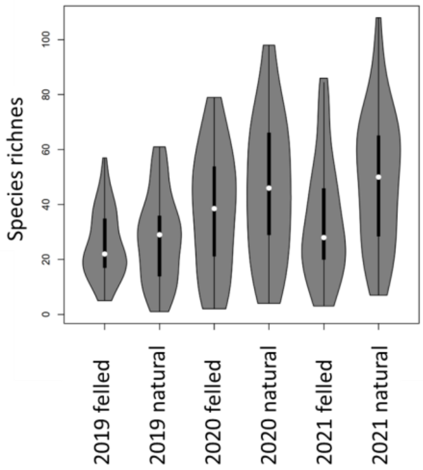

Figure S2. Variation in species (OTU) richness over the years in natural and felled logs.

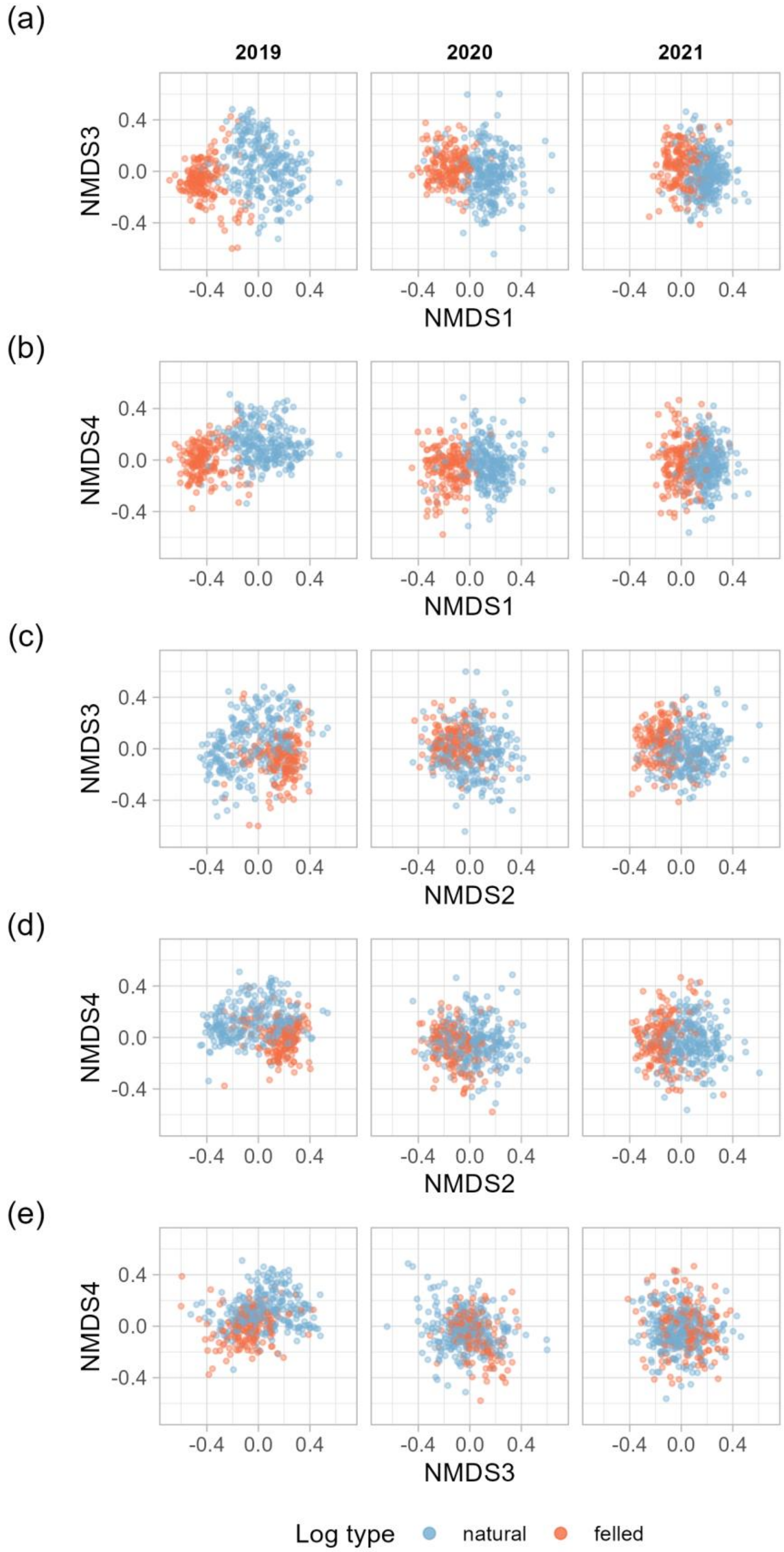

Figure S3. Two-dimensional visualization of a four-dimensional NMDS ordination showing the change in the resident community composition of wood-inhabiting fungi from before the inoculations (2019) to one (2020) and two (2021) years after the inoculations (stress = 0.165; $n_{2019} = 451$, $n_{2020} = 435$, $n_{2021} = 453$) for each additional axis combination (a–e). Points represent logs as sampling units and point color shows the log type.

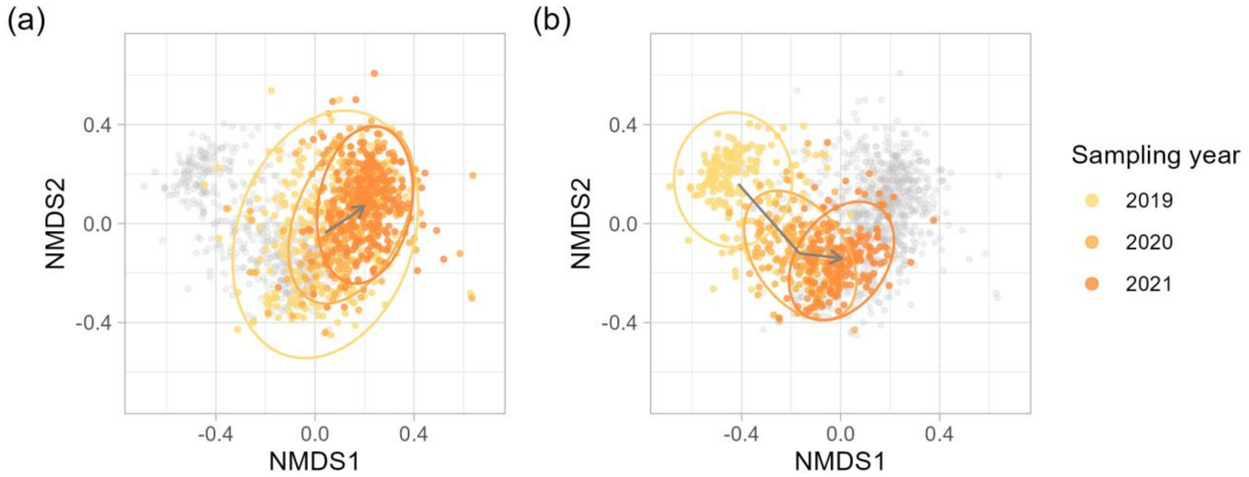

Figure S4. Two-dimensional visualization of a four-dimensional NMDS ordination showing the change in the resident fungal community composition through the three years of monitoring separately for (a) natural (both broken and uprooted logs) and (b) felled logs (stress = 0.165; $n_{natural}$ = 803, n_{felled} = 536; colored points for the targeted log type, points for the other log type shown in grey). Arrows show the average change in community composition from 2019 to 2021 by combining the annual centroids. Points represent logs as sampling units and point color shows the sampling year. Ellipses encompass 95% of the sampling units within each sampling year. Sampling in 2019 was conducted before the inoculation treatment, and the sampling years 2020 and 2021 correspond to time points one and two years after the inoculations.

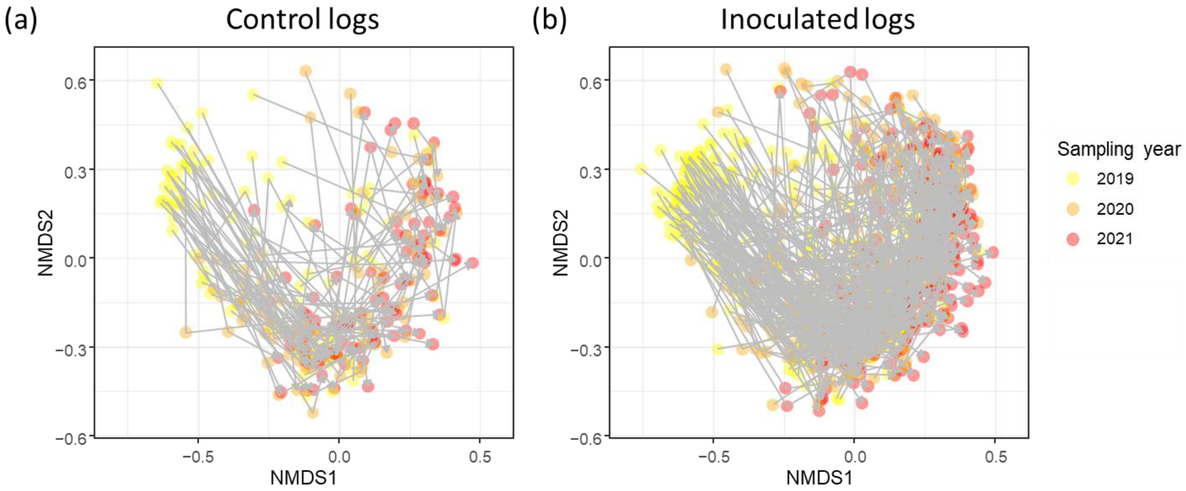

Figure S5. Two-dimensional visualization of a four-dimensional NMDS ordination showing the change in the resident fungal community composition through the three years of monitoring separately for (a) control (b) inoculated logs (stress = 0.176; $n_{control}$ = 295, $n_{inoculated}$ = 1044. Points represent logs as sampling units and point color shows the sampling year. Arrows show the average change in community composition from across the three years per log.

Table S4. Taxonomic assignment for OTUs included in the presence-absence model. OTUs are ordered based on the taxonomic tree in Figure 2 of the main document from top to bottom. Colors corresponding to different phyla are the same as in Figure 2, with *Mucoromycota* shown with yellow, *Basidiomycota* with orange, and *Ascomycota* with blue.

OTU	Phylum	Class	Order	Family	Genus	Species
OTU0224	pseudophylum 1797	pseudoclass 2141	pseudoorder 2693	pseudofamily 3436	pseudogenus 4094	pseudospecies 6471
OTU0159	*Chytridiomycota*	pseudoclass 0266	pseudoorder 0615	pseudofamily 1244	pseudogenus 1726	pseudospecies 3641
OTU0201	*Mucoromycota*	dummy class *Mucorales*	*Mucorales*	*Umbelopsidaceae*	*Umbelopsis*	*Umbelopsis ramanniana*
OTU0051	*Mucoromycota*	dummy class *Mortierellales*	pseudoorder 0287	pseudofamily 0782	pseudogenus 1221	pseudospecies 2903
OTU0162	*Mucoromycota*	pseudoclass 0276	pseudoorder 0627	pseudofamily 1256	pseudogenus 1738	pseudospecies 3660
OTU0042	*Mucoromycota*	pseudoclass 0276	pseudoorder 0627	pseudofamily 1256	pseudogenus 1738	pseudospecies 3659
OTU0223	*Basidiomycota*	pseudoclass 0211	pseudoorder 0554	pseudofamily 1178	pseudogenus 1657	pseudospecies 3549
OTU0178	*Basidiomycota*	pseudoclass 0254	pseudoorder 0600	pseudofamily 1227	pseudogenus 1706	pseudospecies 3606
OTU0170	*Basidiomycota*	pseudoclass 0198	pseudoorder 0533	pseudofamily 1145	pseudogenus 1618	pseudospecies 3483
OTU0145	*Basidiomycota*	pseudoclass 0198	pseudoorder 0533	pseudofamily 1151	pseudogenus 1627	pseudospecies 3495
OTU0127	*Basidiomycota*	Pucciniomycetes	*Septobasidiales*	*Septobasidiaceae*	*Septobasidium*	pseudospecies 0972
OTU0156	*Basidiomycota*	pseudoclass 0197	pseudoorder 0532	pseudofamily 1144	pseudogenus 1616	pseudospecies 3481
OTU0117	*Basidiomycota*	pseudoclass 0197	pseudoorder 0532	pseudofamily 1144	pseudogenus 1616	pseudospecies 3474
OTU0109	*Basidiomycota*	pseudoclass 0197	pseudoorder 0532	pseudofamily 1143	pseudogenus 1615	pseudospecies 3472
OTU0195	*Basidiomycota*	*Dacrymycetes*	*Dacrymycetales*	*Dacrymycetaceae*	*Dacrymyces*	*Dacrymyces stillatus*
OTU0203	*Basidiomycota*	*Dacrymycetes*	pseudoorder 0089	pseudofamily 0546	pseudogenus 0962	pseudospecies 2523
OTU0153	*Basidiomycota*	*Dacrymycetes*	pseudoorder 0089	pseudofamily 0546	pseudogenus 0962	pseudospecies 2529
OTU0132	*Basidiomycota*	*Dacrymycetes*	pseudoorder 0089	pseudofamily 0546	pseudogenus 0962	pseudospecies 2518
OTU0128	*Basidiomycota*	*Dacrymycetes*	pseudoorder 0092	pseudofamily 0551	pseudogenus 0968	pseudospecies 2538
OTU0094	*Basidiomycota*	*Dacrymycetes*	pseudoorder 0088	pseudofamily 0545	pseudogenus 0961	pseudospecies 2516
OTU0197	*Basidiomycota*	*Cystobasidiomycetes*	dummy order *Buckleyzymaceae*	pseudofamily 0391	pseudogenus 0795	pseudospecies 2257
OTU0063	*Basidiomycota*	*Cystobasidiomycetes*	dummy order *Symmetrosporaceae*	pseudofamily 0438	pseudogenus 0847	pseudospecies 2347
OTU0210	*Basidiomycota*	*Agaricostilbomycetes*	pseudoorder 0057	pseudofamily 0509	pseudogenus 0924	pseudospecies 2463
OTU0202	*Basidiomycota*	*Agaricostilbomycetes*	dummy order *Cystobasidiopsis*	pseudofamily 0415	pseudogenus 0820	pseudospecies 2301
OTU0137	*Basidiomycota*	*Agaricostilbomycetes*	pseudoorder 0040	pseudofamily 0492	pseudogenus 0906	pseudospecies 2440
OTU0047	*Basidiomycota*	*Agaricostilbomycetes*	pseudoorder 0039	pseudofamily 0490	pseudogenus 0904	pseudospecies 2433
OTU0191	*Basidiomycota*	*Exobasidiomycetes*	*Exobasidiales*	*Exobasidiaceae*	*Exobasidium*	pseudospecies 0474
OTU0089	*Basidiomycota*	*Exobasidiomycetes*	*Exobasidiales*	*Exobasidiaceae*	*Exobasidium*	*Exobasidium maculosum*
OTU0068	*Basidiomycota*	*Exobasidiomycetes*	*Exobasidiales*	*Exobasidiaceae*	*Exobasidium*	*Exobasidium arescens*
OTU0034	*Basidiomycota*	*Exobasidiomycetes*	*Exobasidiales*	*Exobasidiaceae*	*Exobasidium*	pseudospecies 0470
OTU0031	*Basidiomycota*	*Exobasidiomycetes*	*Exobasidiales*	*Exobasidiaceae*	*Exobasidium*	*Exobasidium bisporum*
OTU0167	*Basidiomycota*	*Tremellomycetes*	pseudoorder 0276	pseudofamily 0769	pseudogenus 1208	pseudospecies 2883
OTU0084	*Basidiomycota*	*Tremellomycetes*	pseudoorder 0273	pseudofamily 0764	pseudogenus 1203	pseudospecies 2877
OTU0139	*Basidiomycota*	*Tremellomycetes*	*Tremellales*	dummy family *Hannaella*	pseudogenus 0299	pseudospecies 1552
OTU0171	*Basidiomycota*	*Tremellomycetes*	*Tremellales*	*Phaeotremellaceae*	pseudogenus 0176	pseudospecies 1388
OTU0123	*Basidiomycota*	*Tremellomycetes*	*Tremellales*	*Phaeotremellaceae*	pseudogenus 0176	pseudospecies 1387
OTU0119	*Basidiomycota*	*Tremellomycetes*	*Tremellales*	*Carcinomycetaceae*	pseudogenus 0038	pseudospecies 1162
OTU0110	*Basidiomycota*	*Tremellomycetes*	*Tremellales*	*Carcinomycetaceae*	pseudogenus 0038	pseudospecies 1161
OTU0099	*Basidiomycota*	*Tremellomycetes*	*Tremellales*	*Carcinomycetaceae*	*Carcinomyces*	pseudospecies 0142
OTU0062	*Basidiomycota*	*Tremellomycetes*	*Tremellales*	*Carcinomycetaceae*	*Carcinomyces*	pseudospecies 0143
OTU0027	*Basidiomycota*	*Tremellomycetes*	*Tremellales*	*Carcinomycetaceae*	*Carcinomyces*	*Carcinomyces polyporina*
OTU0213	*Basidiomycota*	*Tremellomycetes*	*Tremellales*	*Tremellaceae*	pseudogenus 0223	pseudospecies 1455
OTU0138	*Basidiomycota*	*Tremellomycetes*	*Tremellales*	*Tremellaceae*	pseudogenus 0262	pseudospecies 1505
OTU0129	*Basidiomycota*	*Tremellomycetes*	*Tremellales*	*Tremellaceae*	pseudogenus 0246	pseudospecies 1484
OTU0106	*Basidiomycota*	*Tremellomycetes*	*Tremellales*	*Tremellaceae*	pseudogenus 0240	pseudospecies 1477
OTU0154	*Basidiomycota*	*Tremellomycetes*	*Tremellales*	*Tremellaceae*	*Cryptococcus*	pseudospecies 0399
OTU0093	*Basidiomycota*	*Tremellomycetes*	*Tremellales*	*Tremellaceae*	*Cryptococcus*	pseudospecies 0398
OTU0045	*Basidiomycota*	*Tremellomycetes*	*Tremellales*	*Tremellaceae*	*Tremella*	*Tremella encephala*
OTU0022	*Basidiomycota*	*Tremellomycetes*	*Tremellales*	*Tremellaceae*	pseudogenus 0218	pseudospecies 1450
OTU0147	*Basidiomycota*	*Tremellomycetes*	pseudoorder 0254	pseudofamily 0745	pseudogenus 1184	pseudospecies 2853
OTU0019	*Basidiomycota*	*Tremellomycetes*	pseudoorder 0254	pseudofamily 0745	pseudogenus 1184	pseudospecies 2850
OTU0169	*Basidiomycota*	*Agaricomycetes*	pseudoorder 0001	pseudofamily 0445	pseudogenus 0854	pseudospecies 2358
OTU0126	*Basidiomycota*	*Agaricomycetes*	*Sebacinales*	pseudofamily 0331	pseudogenus 0733	pseudospecies 2167
OTU0200	*Basidiomycota*	*Agaricomycetes*	*Atheliales*	*Atheliaceae*	*Athelia*	*Athelia decipiens*
OTU0122	*Basidiomycota*	*Agaricomycetes*	*Atheliales*	*Atheliaceae*	*Athelia*	*Athelia acrospora*
OTU0221	*Basidiomycota*	*Agaricomycetes*	*Agaricales*	*Inocybaceae*	*Crepidotus*	pseudospecies 0391
OTU0179	*Basidiomycota*	*Agaricomycetes*	*Agaricales*	*Physalacriaceae*	*Cylindrobasidium*	pseudospecies 0434
OTU0080	*Basidiomycota*	*Agaricomycetes*	*Agaricales*	pseudofamily 0001	pseudogenus 0326	pseudospecies 1603
OTU0220	*Basidiomycota*	*Agaricomycetes*	*Russulales*	*Peniophoraceae*	*Peniophora*	*Peniophora pithya*
OTU0036	*Basidiomycota*	*Agaricomycetes*	*Russulales*	*Bondarzewiaceae*	*Heterobasidion*	*Heterobasidion annosum*
OTU0035	*Basidiomycota*	*Agaricomycetes*	*Hymenochaetales*	dummy family *Trichaptum*	*Trichaptum*	*Trichaptum abietinum*
OTU0029	*Basidiomycota*	*Agaricomycetes*	*Cantharellales*	*Hydnaceae*	*Sistotrema*	*Sistotrema brinkmannii*
OTU0155	*Basidiomycota*	*Agaricomycetes*	*Polyporales*	pseudofamily 0313	pseudogenus 0715	pseudospecies 2133
OTU0079	*Basidiomycota*	*Agaricomycetes*	*Polyporales*	*Fomitopsidaceae*	*Antrodia*	*Antrodia serialis*
OTU0018	*Basidiomycota*	*Agaricomycetes*	*Polyporales*	*Fomitopsidaceae*	*Fomitopsis*	*Fomitopsis pinicola*
OTU0176	*Basidiomycota*	*Agaricomycetes*	*Auriculariales*	*Auriculariaceae*	*Exidia*	*Exidia glandulosa*
OTU0015	*Basidiomycota*	*Agaricomycetes*	*Auriculariales*	*Auriculariaceae*	*Auricularia*	pseudospecies 0061
OTU0131	*Basidiomycota*	*Microbotryomycetes*	dummy order *Slooffia*	dummy family *Slooffia*	*Slooffia*	pseudospecies 1009
OTU0124	*Basidiomycota*	*Microbotryomycetes*	pseudoorder 0161	pseudofamily 0639	pseudogenus 1076	pseudospecies 2710
OTU0092	*Basidiomycota*	*Microbotryomycetes*	dummy order *Trigonosporomyces*	dummy family *Trigonosporomyces*	*Trigonosporomyces*	pseudospecies 1090
OTU0081	*Basidiomycota*	*Microbotryomycetes*	dummy order *Oberwinklerozyma*	pseudofamily 0424	pseudogenus 0829	pseudospecies 2312
OTU0065	*Basidiomycota*	*Microbotryomycetes*	dummy order *Oberwinklerozyma*	dummy family *Oberwinklerozyma*	*Oberwinklerozyma*	*Oberwinklerozyma silvestris*
OTU0225	*Basidiomycota*	*Microbotryomycetes*	*Sporidiobolales*	pseudofamily 0373	pseudogenus 0777	pseudospecies 2232
OTU0165	*Basidiomycota*	*Microbotryomycetes*	*Sporidiobolales*	pseudofamily 0358	pseudogenus 0762	pseudospecies 2213
OTU0134	*Basidiomycota*	*Microbotryomycetes*	*Sporidiobolales*	pseudofamily 0363	pseudogenus 0767	pseudospecies 2218
OTU0193	*Basidiomycota*	*Microbotryomycetes*	*Sporidiobolales*	dummy family *Rhodotorula*	pseudogenus 0316	pseudospecies 1589
OTU0085	*Basidiomycota*	*Microbotryomycetes*	*Sporidiobolales*	dummy family *Rhodotorula*	pseudogenus 0315	pseudospecies 1588
OTU0070	*Basidiomycota*	*Microbotryomycetes*	*Sporidiobolales*	dummy family *Rhodotorula*	*Rhodotorula*	pseudospecies 0946
OTU0030	*Basidiomycota*	*Microbotryomycetes*	*Sporidiobolales*	dummy family *Rhodotorula*	*Rhodotorula*	pseudospecies 0944
OTU0151	*Basidiomycota*	*Microbotryomycetes*	*Heterogastridiales*	pseudofamily 0176	pseudogenus 0568	pseudospecies 1910
OTU0014	*Basidiomycota*	*Microbotryomycetes*	*Heterogastridiales*	*Heterogastridiaceae*	*Colacogloea*	pseudospecies 0313
OTU0190	*Basidiomycota*	*Microbotryomycetes*	dummy order *Chrysozymaceae*	pseudofamily 0404	pseudogenus 0809	pseudospecies 2282
OTU0023	*Basidiomycota*	*Microbotryomycetes*	dummy order *Chrysozymaceae*	*Chrysozymaceae*	*Hamamotoa*	*Hamamotoa singularis*

OTU0009	*Basidiomycota*	*Microbotryomycetes*	dummy order *Chrysozymaceae*	*Chrysozymaceae*	*Hamamotoa*	*Hamamotoa lignophila*

OTU	Phylum	Class	Order	Family	Genus	Species
OTU0219	*Ascomycota*	pseudoclass 0056	pseudoorder 0351	pseudofamily 0848	pseudogenus 1287	pseudospecies 3012
OTU0184	*Ascomycota*	pseudoclass 0016	pseudoorder 0308	pseudofamily 0804	pseudogenus 1243	pseudospecies 2956
OTU0112	*Ascomycota*	pseudoclass 0076	pseudoorder 0373	pseudofamily 0875	pseudogenus 1316	pseudospecies 3046
OTU0105	*Ascomycota*	pseudoclass 0002	pseudoorder 0292	pseudofamily 0788	pseudogenus 1227	pseudospecies 2935
OTU0182	*Ascomycota*	pseudoclass 0162	pseudoorder 0464	pseudofamily 0981	pseudogenus 1430	pseudospecies 3190
OTU0172	*Ascomycota*	pseudoclass 0162	pseudoorder 0464	pseudofamily 0981	pseudogenus 1430	pseudospecies 3189
OTU0076	*Ascomycota*	pseudoclass 0162	pseudoorder 0464	pseudofamily 0981	pseudogenus 1430	pseudospecies 3192
OTU0044	*Ascomycota*	pseudoclass 0117	pseudoorder 0417	pseudofamily 0929	pseudogenus 1376	pseudospecies 3124
OTU0121	*Ascomycota*	*Saccharomycetes*	dummy order *Danielozyma*	dummy family *Danielozyma*	pseudogenus 0296	pseudospecies 1549
OTU0187	*Ascomycota*	*Saccharomycetes*	*Saccharomycetales*	pseudofamily 0328	pseudogenus 0730	pseudospecies 2160
OTU0163	*Ascomycota*	*Saccharomycetes*	*Saccharomycetales*	pseudofamily 0330	pseudogenus 0732	pseudospecies 2166
OTU0136	*Ascomycota*	*Saccharomycetes*	*Saccharomycetales*	dummy family *Candida*	*Candida*	*Candida tenuis*
OTU0133	*Ascomycota*	*Saccharomycetes*	*Saccharomycetales*	*Debaryomycetaceae*	*Scheffersomyces*	*Scheffersomyces ergatensis*
OTU0116	*Ascomycota*	*Saccharomycetes*	*Saccharomycetales*	dummy family *Yamadazyma*	*Yamadazyma*	*Yamadazyma scolyti*
OTU0111	*Ascomycota*	*Saccharomycetes*	*Saccharomycetales*	dummy family *Peterozyma*	*Peterozyma*	*Peterozyma xylosa*
OTU0078	*Ascomycota*	*Saccharomycetes*	*Saccharomycetales*	dummy family *Peterozyma*	*Peterozyma*	*Peterozyma toletana*
OTU0073	*Ascomycota*	*Saccharomycetes*	*Saccharomycetales*	dummy family *Nakazawaea*	*Nakazawaea*	*Nakazawaea holstii*
OTU0060	*Ascomycota*	*Saccharomycetes*	*Saccharomycetales*	pseudofamily 0322	pseudogenus 0724	pseudospecies 2144
OTU0053	*Ascomycota*	*Saccharomycetes*	*Saccharomycetales*	pseudofamily 0322	pseudogenus 0724	pseudospecies 2143
OTU0183	*Ascomycota*	*Saccharomycetes*	*Saccharomycetales*	*Saccharomycetaceae*	*Ogataea*	*Ogataea pini*
OTU0125	*Ascomycota*	*Saccharomycetes*	*Saccharomycetales*	*Saccharomycetaceae*	*Ogataea*	*Ogataea neopini*
OTU0088	*Ascomycota*	*Saccharomycetes*	*Saccharomycetales*	*Saccharomycetaceae*	*Ogataea*	*Ogataea ramenticola*
OTU0066	*Ascomycota*	*Saccharomycetes*	*Saccharomycetales*	*Saccharomycetaceae*	pseudogenus 0197	pseudospecies 1413
OTU0095	*Ascomycota*	*Saccharomycetes*	*Saccharomycetales*	*Saccharomycetaceae*	*Kuraishia*	*Kuraishia molischiana*
OTU0040	*Ascomycota*	*Saccharomycetes*	*Saccharomycetales*	*Saccharomycetaceae*	*Kuraishia*	*Kuraishia capsulata*
OTU0038	*Ascomycota*	pseudoclass 0001	pseudoorder 0291	pseudofamily 0786	pseudogenus 1225	pseudospecies 2932
OTU0192	*Ascomycota*	pseudoclass 0177	pseudoorder 0507	pseudofamily 1114	pseudogenus 1585	pseudospecies 3438
OTU0168	*Ascomycota*	pseudoclass 0177	pseudoorder 0507	pseudofamily 1117	pseudogenus 1588	pseudospecies 3442
OTU0199	*Ascomycota*	pseudoclass 0177	pseudoorder 0504	pseudofamily 1083	pseudogenus 1551	pseudospecies 3370
OTU0114	*Ascomycota*	pseudoclass 0177	pseudoorder 0504	pseudofamily 1103	pseudogenus 1573	pseudospecies 3418
OTU0108	*Ascomycota*	pseudoclass 0177	pseudoorder 0504	pseudofamily 1078	pseudogenus 1546	pseudospecies 3363
OTU0083	*Ascomycota*	pseudoclass 0177	pseudoorder 0504	pseudofamily 1069	pseudogenus 1525	pseudospecies 3321
OTU0046	*Ascomycota*	pseudoclass 0177	pseudoorder 0504	pseudofamily 1072	pseudogenus 1539	pseudospecies 3353
OTU0033	*Ascomycota*	pseudoclass 0177	pseudoorder 0484	pseudofamily 1002	pseudogenus 1452	pseudospecies 3221
OTU0206	*Ascomycota*	*Dothideomycetes*	dummy order *Pyrenochaeta*	dummy family *Pyrenochaeta*	*Pyrenochaeta*	*Pyrenochaeta cava*
OTU0141	*Ascomycota*	*Dothideomycetes*	*Venturiales*	*Venturiaceae*	*Rhizosphaera*	*Rhizosphaera kalkhoffii*
OTU0150	*Ascomycota*	*Dothideomycetes*	*Mytilinidiales*	pseudofamily 0266	pseudogenus 0661	pseudospecies 2038
OTU0135	*Ascomycota*	*Dothideomycetes*	*Mytilinidiales*	*Mytilinidiaceae*	*Lophium*	*Lophium mytilinum*
OTU0216	*Ascomycota*	*Dothideomycetes*	*Pleosporales*	*Didymellaceae*	*Epicoccum*	*Epicoccum nigrum*
OTU0143	*Ascomycota*	*Dothideomycetes*	*Pleosporales*	pseudofamily 0275	pseudogenus 0673	pseudospecies 2074
OTU0104	*Ascomycota*	*Dothideomycetes*	*Pleosporales*	pseudofamily 0274	pseudogenus 0669	pseudospecies 2052
OTU0102	*Ascomycota*	*Dothideomycetes*	*Pleosporales*	*Pleosporaceae*	pseudogenus 0189	pseudospecies 1403
OTU0074	*Ascomycota*	*Dothideomycetes*	*Dothideales*	dummy family *Celosporium*	*Celosporium*	pseudospecies 0145
OTU0173	*Ascomycota*	*Dothideomycetes*	*Dothideales*	*Dothioraceae*	*Hormonema*	*Hormonema carpetanum*
OTU0041	*Ascomycota*	*Dothideomycetes*	*Dothideales*	*Dothioraceae*	*Sydowia*	*Sydowia polyspora*
OTU0212	*Ascomycota*	*Dothideomycetes*	*Botryosphaeriales*	pseudofamily 0036	pseudogenus 0367	pseudospecies 1653
OTU0039	*Ascomycota*	*Dothideomycetes*	*Botryosphaeriales*	pseudofamily 0036	pseudogenus 0364	pseudospecies 1650
OTU0215	*Ascomycota*	*Dothideomycetes*	*Capnodiales*	*Mycosphaerellaceae*	pseudogenus 0143	pseudospecies 1331
OTU0075	*Ascomycota*	*Dothideomycetes*	*Capnodiales*	pseudofamily 0076	pseudogenus 0445	pseudospecies 1750
OTU0130	*Ascomycota*	*Dothideomycetes*	*Capnodiales*	*Teratosphaeriaceae*	pseudogenus 0208	pseudospecies 1427
OTU0050	*Ascomycota*	*Dothideomycetes*	*Capnodiales*	*Teratosphaeriaceae*	pseudogenus 0208	pseudospecies 1426
OTU0211	*Ascomycota*	*Dothideomycetes*	*Capnodiales*	pseudofamily 0075	pseudogenus 0444	pseudospecies 1749
OTU0214	*Ascomycota*	*Dothideomycetes*	*Capnodiales*	pseudofamily 0075	pseudogenus 0442	pseudospecies 1744
OTU0097	*Ascomycota*	*Dothideomycetes*	*Capnodiales*	pseudofamily 0075	pseudogenus 0442	pseudospecies 1743
OTU0037	*Ascomycota*	*Dothideomycetes*	*Capnodiales*	pseudofamily 0075	pseudogenus 0440	pseudospecies 1740
OTU0181	*Ascomycota*	*Dothideomycetes*	*Capnodiales*	*Cladosporiaceae*	*Cladosporium*	pseudospecies 0298
OTU0061	*Ascomycota*	*Dothideomycetes*	*Capnodiales*	*Cladosporiaceae*	*Cladosporium*	pseudospecies 0297
OTU0012	*Ascomycota*	*Dothideomycetes*	*Capnodiales*	*Cladosporiaceae*	*Cladosporium*	*Cladosporium grevilleae*
OTU0204	*Ascomycota*	*Lecanoromycetes*	pseudoorder 0109	pseudofamily 0571	pseudogenus 0993	pseudospecies 2586
OTU0194	*Ascomycota*	*Lecanoromycetes*	dummy order *Fuscideaceae*	*Fuscideaceae*	*Fuscidea*	*Fuscidea pusilla*
OTU0185	*Ascomycota*	*Lecanoromycetes*	*Baeomycetales*	*Trapeliaceae*	*Sarea*	*Sarea resinae*
OTU0177	*Ascomycota*	*Lecanoromycetes*	pseudoorder 0135	pseudofamily 0609	pseudogenus 1043	pseudospecies 2665
OTU0196	*Ascomycota*	*Lecanoromycetes*	*Lecanorales*	*Pilocarpaceae*	*Micarea*	pseudospecies 0727
OTU0120	*Ascomycota*	*Lecanoromycetes*	*Lecanorales*	*Parmeliaceae*	*Platismatia*	*Platismatia glauca*
OTU0057	*Ascomycota*	*Lecanoromycetes*	*Lecanorales*	*Parmeliaceae*	*Hypogymnia*	*Hypogymnia physodes*
OTU0049	*Ascomycota*	*Lecanoromycetes*	pseudoorder 0120	pseudofamily 0589	pseudogenus 1019	pseudospecies 2629
OTU0103	*Ascomycota*	*Lecanoromycetes*	pseudoorder 0108	pseudofamily 0569	pseudogenus 0989	pseudospecies 2578
OTU0011	*Ascomycota*	*Lecanoromycetes*	pseudoorder 0108	pseudofamily 0569	pseudogenus 0988	pseudospecies 2570
OTU0186	*Ascomycota*	*Sordariomycetes*	pseudoorder 0229	pseudofamily 0718	pseudogenus 1157	pseudospecies 2819
OTU0152	*Ascomycota*	*Sordariomycetes*	pseudoorder 0205	pseudofamily 0690	pseudogenus 1127	pseudospecies 2785
OTU0188	*Ascomycota*	*Sordariomycetes*	*Hypocreales*	*Niessliaceae*	pseudogenus 0167	pseudospecies 1376
OTU0180	*Ascomycota*	*Sordariomycetes*	*Hypocreales*	pseudofamily 0219	pseudogenus 0611	pseudospecies 1969
OTU0160	*Ascomycota*	*Sordariomycetes*	*Hypocreales*	*Hypocreaceae*	*Trichoderma*	pseudospecies 1087
OTU0142	*Ascomycota*	*Sordariomycetes*	*Hypocreales*	pseudofamily 0185	pseudogenus 0577	pseudospecies 1924
OTU0059	*Ascomycota*	*Sordariomycetes*	*Hypocreales*	*Nectriaceae*	*Fusarium*	pseudospecies 0527
OTU0140	*Ascomycota*	*Sordariomycetes*	*Microascales*	pseudofamily 0257	pseudogenus 0651	pseudospecies 2021
OTU0098	*Ascomycota*	*Sordariomycetes*	*Microascales*	dummy family *Chalara*	*Chalara*	pseudospecies 0168
OTU0056	*Ascomycota*	*Sordariomycetes*	*Microascales*	dummy family *Endoconidiophora*	*Endoconidiophora*	*Endoconidiophora polonica*
OTU0006	*Ascomycota*	*Sordariomycetes*	*Lulworthiales*	*Lulworthiaceae*	*Zalerion*	*Zalerion arboricola*
OTU0198	*Ascomycota*	*Sordariomycetes*	*Coniochaetales*	pseudofamily 0097	pseudogenus 0480	pseudospecies 1802
OTU0205	*Ascomycota*	*Sordariomycetes*	*Coniochaetales*	*Coniochaetaceae*	*Coniochaeta*	*Coniochaeta fasciculata*
OTU0072	*Ascomycota*	*Sordariomycetes*	*Coniochaetales*	*Coniochaetaceae*	pseudogenus 0050	pseudospecies 1183
OTU0048	*Ascomycota*	*Sordariomycetes*	*Coniochaetales*	*Coniochaetaceae*	pseudogenus 0050	pseudospecies 1176
OTU0004	*Ascomycota*	*Sordariomycetes*	*Coniochaetales*	*Coniochaetaceae*	pseudogenus 0050	pseudospecies 1175
OTU0096	*Ascomycota*	*Eurotiomycetes*	*Phaeomoniellales*	*Phaeomoniellaceae*	*Phaeomoniella*	pseudospecies 0835
OTU0161	*Ascomycota*	*Eurotiomycetes*	*Chaetothyriales*	*Herpotrichiellaceae*	pseudogenus 0099	pseudospecies 1262
OTU0217	*Ascomycota*	*Eurotiomycetes*	*Chaetothyriales*	*Herpotrichiellaceae*	*Capronia*	*Capronia semi-immersa*
OTU0164	*Ascomycota*	*Eurotiomycetes*	*Chaetothyriales*	*Herpotrichiellaceae*	*Capronia*	pseudospecies 0118
OTU0107	*Ascomycota*	*Eurotiomycetes*	*Chaetothyriales*	*Herpotrichiellaceae*	*Capronia*	pseudospecies 0119
OTU0091	*Ascomycota*	*Eurotiomycetes*	*Chaetothyriales*	*Herpotrichiellaceae*	*Rhinocladiella*	*Rhinocladiella atrovirens*

OTU0010	*Ascomycota*	*Eurotiomycetes*	*Chaetothyriales*	*Herpotrichiellaceae*	pseudogenus 0087	pseudospecies 1245
OTU0209	*Ascomycota*	*Eurotiomycetes*	*Chaetothyriales*	*Herpotrichiellaceae*	*Cladophialophora*	pseudospecies 0214
OTU0166	*Ascomycota*	*Eurotiomycetes*	*Chaetothyriales*	*Herpotrichiellaceae*	*Cladophialophora*	pseudospecies 0219
OTU0158	*Ascomycota*	*Eurotiomycetes*	*Chaetothyriales*	*Herpotrichiellaceae*	*Cladophialophora*	pseudospecies 0236
**OTU**	**Phylum**	**Class**	**Order**	**Family**	**Genus**	**Species**
OTU0148	*Ascomycota*	*Eurotiomycetes*	*Chaetothyriales*	*Herpotrichiellaceae*	*Cladophialophora*	*Cladophialophora chaetospira*
OTU0115	*Ascomycota*	*Eurotiomycetes*	*Chaetothyriales*	*Herpotrichiellaceae*	*Cladophialophora*	pseudospecies 0206
OTU0077	*Ascomycota*	*Eurotiomycetes*	*Chaetothyriales*	*Herpotrichiellaceae*	*Cladophialophora*	pseudospecies 0286
OTU0071	*Ascomycota*	*Eurotiomycetes*	*Chaetothyriales*	*Herpotrichiellaceae*	*Cladophialophora*	pseudospecies 0290
OTU0064	*Ascomycota*	*Eurotiomycetes*	*Chaetothyriales*	*Herpotrichiellaceae*	*Cladophialophora*	pseudospecies 0280
OTU0043	*Ascomycota*	*Eurotiomycetes*	*Chaetothyriales*	*Herpotrichiellaceae*	*Cladophialophora*	pseudospecies 0293
OTU0021	*Ascomycota*	*Eurotiomycetes*	*Chaetothyriales*	*Herpotrichiellaceae*	*Cladophialophora*	pseudospecies 0199
OTU0013	*Ascomycota*	*Eurotiomycetes*	*Chaetothyriales*	*Herpotrichiellaceae*	*Cladophialophora*	pseudospecies 0223
OTU0008	*Ascomycota*	*Eurotiomycetes*	*Chaetothyriales*	*Herpotrichiellaceae*	*Cladophialophora*	pseudospecies 0253
OTU0005	*Ascomycota*	*Eurotiomycetes*	*Chaetothyriales*	*Herpotrichiellaceae*	*Cladophialophora*	pseudospecies 0198
OTU0218	*Ascomycota*	*Eurotiomycetes*	*Chaetothyriales*	*Herpotrichiellaceae*	*Exophiala*	pseudospecies 0506
OTU0003	*Ascomycota*	*Eurotiomycetes*	*Chaetothyriales*	*Herpotrichiellaceae*	*Exophiala*	pseudospecies 0486
OTU0100	*Ascomycota*	*Leotiomycetes*	*Rhytismatales*	pseudofamily 0318	pseudogenus 0720	pseudospecies 2138
OTU0207	*Ascomycota*	*Leotiomycetes*	*Helotiales*	*Leotiaceae*	pseudogenus 0133	pseudospecies 1316
OTU0157	*Ascomycota*	*Leotiomycetes*	*Helotiales*	pseudofamily 0130	pseudogenus 0516	pseudospecies 1849
OTU0144	*Ascomycota*	*Leotiomycetes*	*Helotiales*	*Dermateaceae*	pseudogenus 0062	pseudospecies 1207
OTU0118	*Ascomycota*	*Leotiomycetes*	*Helotiales*	*Dermateaceae*	*Mollisia*	*Mollisia cinerea*
OTU0090	*Ascomycota*	*Leotiomycetes*	*Helotiales*	*Phacidiaceae*	*Phacidium*	pseudospecies 0832
OTU0055	*Ascomycota*	*Leotiomycetes*	*Helotiales*	pseudofamily 0134	pseudogenus 0522	pseudospecies 1858
OTU0222	*Ascomycota*	*Leotiomycetes*	*Helotiales*	*Helotiaceae*	*Hymenoscyphus*	pseudospecies 0612
OTU0101	*Ascomycota*	*Leotiomycetes*	*Helotiales*	*Helotiaceae*	*Hymenoscyphus*	pseudospecies 0603
OTU0069	*Ascomycota*	*Leotiomycetes*	*Helotiales*	*Helotiaceae*	pseudogenus 0077	pseudospecies 1227
OTU0086	*Ascomycota*	*Leotiomycetes*	*Helotiales*	*Helotiaceae*	*Ascocoryne*	*Ascocoryne sarcoides*
OTU0026	*Ascomycota*	*Leotiomycetes*	*Helotiales*	*Helotiaceae*	*Ascocoryne*	*Ascocoryne cylichnium*
OTU0146	*Ascomycota*	*Leotiomycetes*	*Helotiales*	*Vibrisseaceae*	*Phialocephala*	*Phialocephala piceae*
OTU0087	*Ascomycota*	*Leotiomycetes*	*Helotiales*	*Vibrisseaceae*	*Phialocephala*	*Phialocephala lagerbergii*
OTU0052	*Ascomycota*	*Leotiomycetes*	*Helotiales*	*Vibrisseaceae*	*Phialocephala*	pseudospecies 0865
OTU0025	*Ascomycota*	*Leotiomycetes*	*Helotiales*	*Vibrisseaceae*	*Phialocephala*	*Phialocephala scopiformis*
OTU0024	*Ascomycota*	*Leotiomycetes*	*Helotiales*	dummy family *Leptodontidium*	*Leptodontidium*	*Leptodontidium trabinellum*
OTU0149	*Ascomycota*	*Leotiomycetes*	*Helotiales*	*Hyaloscyphaceae*	*Hyaloscypha*	pseudospecies 0584
OTU0067	*Ascomycota*	*Leotiomycetes*	*Helotiales*	*Hyaloscyphaceae*	*Hyaloscypha*	*Hyaloscypha aureliella*
OTU0208	*Ascomycota*	*Leotiomycetes*	*Helotiales*	*Hyaloscyphaceae*	*Hyphodiscus*	pseudospecies 0621
OTU0174	*Ascomycota*	*Leotiomycetes*	*Helotiales*	*Hyaloscyphaceae*	*Hyphodiscus*	*Hyphodiscus brachyconius*
OTU0113	*Ascomycota*	*Leotiomycetes*	*Helotiales*	*Hyaloscyphaceae*	*Hyphodiscus*	pseudospecies 0620
OTU0028	*Ascomycota*	*Leotiomycetes*	*Helotiales*	*Hyaloscyphaceae*	*Hyphodiscus*	*Hyphodiscus hymeniophilus*
OTU0054	*Ascomycota*	*Leotiomycetes*	*Helotiales*	*Hyaloscyphaceae*	*Ciliolarina*	pseudospecies 0194
OTU0017	*Ascomycota*	*Leotiomycetes*	*Helotiales*	*Hyaloscyphaceae*	*Ciliolarina*	*Ciliolarina pinicola*
OTU0082	*Ascomycota*	*Leotiomycetes*	*Helotiales*	pseudofamily 0119	pseudogenus 0502	pseudospecies 1829
OTU0020	*Ascomycota*	*Leotiomycetes*	*Helotiales*	pseudofamily 0119	pseudogenus 0502	pseudospecies 1832
OTU0016	*Ascomycota*	*Leotiomycetes*	*Helotiales*	pseudofamily 0119	pseudogenus 0502	pseudospecies 1828
OTU0007	*Ascomycota*	*Leotiomycetes*	*Helotiales*	dummy family *Infundichalara*	*Infundichalara*	pseudospecies 0658
OTU0189	*Ascomycota*	*Leotiomycetes*	*Helotiales*	*Tympanidaceae*	*Collophora*	pseudospecies 0345
OTU0175	*Ascomycota*	*Leotiomycetes*	*Helotiales*	*Tympanidaceae*	*Collophora*	pseudospecies 0340
OTU0058	*Ascomycota*	*Leotiomycetes*	*Helotiales*	*Tympanidaceae*	*Collophora*	pseudospecies 0325
OTU0032	*Ascomycota*	*Leotiomycetes*	*Helotiales*	*Tympanidaceae*	*Collophora*	pseudospecies 0321
OTU0002	*Ascomycota*	*Leotiomycetes*	*Helotiales*	*Tympanidaceae*	*Collophora*	pseudospecies 0320
OTU0001	*Ascomycota*	*Leotiomycetes*	*Helotiales*	dummy family *Xenopolyscytalum*	*Xenopolyscytalum*	*Xenopolyscytalum pinea*

Table S5. Posterior means for regression parameters (Beta) in the presence-absence model describing the responses of each OTU to each predictor in the model. OTUs are arranged in the same order as in Figure 2, following their taxonomic relationships, and colored based on their phyla as in Figure 2: OTUs assigned to *Mucoromycota* are shown with yellow, *Basidiomycota* with orange, and *Ascomycota* in blue. Felled log type, sampling year 2019, broken mortality factor, and the interaction terms between the control treatment and sampling years 2020 and 2021 and between the control treatment and felled log type are included in the intercept as reference levels.

	Predictor																																
OTU	(Intercept)	Log type: natural	Year: 2020	Year: 2021	Mortality factor: uprooted	Decay stage	Sequencing depth	Log type: natural x Year: 2020	Log type: natural x Year: 2021	Year: 2020 x *Antrodia piceata*	Year: 2020 x *Antrodiella citrinella*	Year: 2020 x *Fomitopsis rosea*	Year: 2020 x *Perenniporia subacida*	Year: 2020 x *Physisporinus crocatus*	Year: 2020 x *Postia guttulata*	Year: 2020 x *Skeletocutis odora*	Year: 2020 x *Skeletocutis stellae*	Year: 2020 x *Steccherinum collabens*	Year: 2021 x *Antrodia piceata*	Year: 2021 x *Antrodiella citrinella*	Year: 2021 x *Fomitopsis rosea*	Year: 2021 x *Perenniporia subacida*	Year: 2021 x *Physisporinus crocatus*	Year: 2021 x *Postia guttulata*	Year: 2021 x *Skeletocutis odora*	Year: 2021 x *Skeletocutis stellae*	Year: 2021 x *Steccherinum collabens*	Log type: natural x *Antrodia piceata*	Log type: natural x *Antrodiella citrinella*	Log type: natural x *Fomitopsis rosea*	Log type: natural x *Perenniporia subacida*	Log type: natural x *Physisporinus crocatus*	Log type: natural x *Postia guttulata*	Log type: natural x *Skeletocutis odora*	Log type: natural x *Skeletocutis stellae*	Log type: natural x *Steccherinum collabens*
OTU0224	-5.80	0.25	0.84	0.74	0.03	-0.08	0.32	-0.36	-0.39	-0.15	0.10	0.15	-0.15	-0.23	-0.06	-0.10	0.05	0.03	-0.25	0.07	-0.04	-0.21	-0.13	-0.23	-0.04	-0.07	0.02	0.02	0.02	0.15	0.04	0.09	-0.10	0.10	0.18	-0.01
OTU0159	-4.95	-0.32	-0.21	-0.07	0.03	0.13	0.30	0.59	0.51	-0.15	-0.15	-0.03	-0.17	-0.35	-0.03	-0.14	-0.27	0.01	-0.16	-0.20	0.11	-0.14	-0.24	-0.19	-0.10	-0.27	0.06	0.17	0.16	0.03	0.17	0.20	0.06	0.08	0.25	-0.04
OTU0201	-3.72	0.97	-0.52	-0.45	-0.06	0.00	0.17	-0.45	-0.51	-0.12	-0.23	0.14	-0.22	-0.16	-0.22	-0.10	0.02	-0.02	-0.26	-0.26	-0.02	-0.18	-0.18	-0.30	-0.13	-0.19	0.04	0.15	0.38	0.04	0.22	0.02	0.12	-0.01	0.19	-0.24
OTU0051	-9.26	1.44	0.68	0.87	0.03	0.03	0.59	-0.32	-0.45	-0.08	-0.02	0.36	-0.16	-0.08	-0.07	-0.19	-0.07	-0.03	-0.21	0.05	0.04	-0.14	-0.13	-0.29	-0.18	-0.14	0.03	0.06	0.19	0.02	0.13	0.06	0.08	0.22	0.37	-0.08
OTU0162	-4.92	0.40	1.26	0.67	0.77	-0.31	0.29	-1.18	-1.46	-0.02	-0.03	-0.05	-0.15	-0.01	-0.14	-0.09	-0.11	-0.01	-0.32	-0.14	0.01	-0.48	0.19	-0.10	-0.27	-0.09	0.10	-0.01	-0.21	0.16	0.09	-0.06	0.17	-0.34	0.02	-0.21
OTU0042	-7.34	2.19	2.43	2.68	0.04	-0.49	0.42	-1.78	-2.40	0.04	0.26	0.10	-0.13	0.10	-0.05	0.03	-0.13	0.07	-0.19	0.13	0.01	-0.48	0.11	-0.08	-0.25	-0.09	0.23	0.00	-0.34	0.11	0.08	-0.19	0.01	-0.24	0.18	-0.13
OTU0223	-7.33	2.37	1.04	1.77	-0.01	0.11	0.23	-0.87	-1.27	-0.01	-0.05	0.33	-0.02	-0.05	-0.04	0.14	-0.04	0.01	-0.09	0.10	0.24	0.12	-0.24	0.03	0.15	-0.27	0.16	0.07	0.00	0.05	0.00	0.05	0.05	0.11	0.11	0.02
OTU0178	-9.97	1.13	2.07	2.26	0.31	0.07	0.54	-0.87	-1.17	0.00	0.08	0.17	0.03	-0.11	0.03	0.01	-0.08	-0.16	-0.25	0.25	0.01	-0.06	0.05	-0.10	-0.03	-0.08	-0.04	-0.06	0.04	-0.02	-0.12	0.14	0.11	-0.20	0.09	-0.05
OTU0170	-8.77	1.86	0.81	1.30	0.31	0.44	0.37	-0.43	-0.87	0.02	-0.09	0.49	-0.05	-0.10	0.02	0.03	-0.05	-0.02	-0.12	0.07	0.28	0.05	-0.10	-0.08	0.11	-0.46	0.10	0.05	0.17	-0.01	0.17	0.21	0.07	-0.04	0.04	0.12
OTU0145	-7.68	0.79	0.55	0.45	0.15	0.12	0.43	0.05	-0.03	-0.08	-0.15	0.37	-0.21	-0.26	0.02	0.06	-0.08	0.02	-0.05	0.08	0.29	-0.10	-0.19	-0.19	0.06	-0.36	0.10	0.14	0.09	-0.04	0.24	0.08	-0.03	0.16	0.20	0.13
OTU0127	-10.91	0.96	2.48	2.95	0.36	0.00	0.59	-0.69	-0.67	-0.11	-0.03	0.25	0.21	-0.07	0.12	-0.02	-0.12	-0.15	-0.18	0.39	0.08	0.11	0.00	-0.16	0.15	-0.14	0.04	-0.10	0.02	0.00	-0.10	0.06	0.09	-0.07	0.08	0.02
OTU0156	-5.65	0.71	1.02	1.29	0.20	-0.11	0.28	-0.65	-0.86	0.00	0.00	0.12	-0.15	-0.25	0.01	-0.24	-0.16	-0.13	-0.26	0.02	-0.01	-0.20	-0.02	-0.23	-0.20	-0.24	-0.01	0.01	-0.03	0.10	-0.01	0.13	0.00	-0.07	0.03	-0.17
OTU0117	-5.36	0.90	0.64	1.34	0.24	-0.03	0.28	-0.35	-0.73	0.04	0.07	0.06	-0.19	-0.16	-0.10	-0.32	-0.25	-0.19	-0.27	0.01	0.02	-0.23	-0.08	-0.27	-0.32	-0.36	-0.08	0.03	-0.03	0.13	-0.02	0.14	0.06	-0.06	0.10	-0.17
OTU0109	-6.10	-0.04	0.43	0.21	0.09	-0.21	0.43	0.02	-0.07	-0.06	-0.02	0.07	-0.25	-0.36	-0.03	-0.33	-0.18	-0.06	-0.19	-0.09	0.03	-0.36	-0.20	-0.27	-0.33	-0.30	0.06	0.04	0.11	0.06	0.02	0.26	0.04	0.10	0.01	-0.12
OTU0195	-9.24	2.28	1.44	1.98	0.19	0.25	0.38	-1.01	-1.37	-0.12	-0.02	0.18	0.00	-0.13	0.10	0.11	0.00	-0.08	-0.22	0.22	0.06	0.07	-0.20	-0.13	0.13	-0.16	0.04	-0.15	-0.12	0.05	0.00	0.08	0.15	-0.14	0.11	0.04
OTU0203	-6.64	1.67	1.16	1.33	0.57	-0.37	0.34	-1.00	-1.16	0.09	0.03	0.04	-0.28	-0.22	0.01	-0.12	-0.08	-0.22	-0.10	0.08	-0.17	-0.15	-0.27	-0.23	-0.29	-0.25	0.05	-0.20	-0.26	0.10	0.11	-0.03	0.14	-0.08	0.12	-0.04
OTU0153	-7.59	2.35	1.73	1.99	0.39	-0.36	0.37	-1.43	-1.66	0.08	0.18	0.16	-0.26	-0.16	0.06	-0.05	0.02	-0.17	-0.12	0.13	-0.05	-0.16	-0.28	-0.15	-0.25	-0.23	0.06	-0.24	-0.32	0.12	0.05	-0.06	0.03	-0.14	0.10	0.01
OTU0132	-7.19	2.04	1.54	1.72	0.53	-0.10	0.34	-1.19	-1.34	0.09	0.10	0.03	-0.28	-0.17	0.08	-0.14	0.06	-0.24	-0.11	0.14	-0.13	-0.13	-0.28	-0.22	-0.24	-0.22	0.06	-0.21	-0.31	0.10	0.14	-0.06	0.08	-0.06	0.20	-0.08
OTU0128	-7.24	1.53	1.20	1.41	0.21	0.15	0.34	-0.85	-1.10	0.02	-0.05	0.27	-0.14	-0.19	0.08	-0.01	-0.15	-0.15	-0.15	0.04	0.06	-0.08	-0.24	-0.12	-0.13	-0.28	0.14	-0.04	-0.08	-0.01	0.15	0.10	0.01	-0.08	0.15	0.06
OTU0094	-7.54	1.64	1.55	1.48	0.51	-0.09	0.40	-1.46	-1.59	-0.03	0.06	0.05	-0.21	-0.13	0.07	-0.07	-0.09	-0.13	-0.13	0.01	-0.07	-0.28	-0.24	-0.24	-0.18	-0.17	0.01	-0.14	-0.23	0.15	0.11	-0.02	0.10	-0.24	0.04	-0.05
OTU0197	-6.25	-0.36	0.16	-0.26	-0.11	-0.22	0.43	0.34	0.45	-0.27	-0.24	0.11	-0.17	-0.29	-0.06	-0.05	-0.12	0.00	-0.10	-0.11	-0.05	-0.12	-0.17	-0.24	-0.06	-0.20	0.13	0.07	0.17	0.00	0.16	0.05	0.05	0.01	-0.02	-0.04
OTU0063	-7.47	0.24	0.46	0.45	0.01	-0.05	0.50	0.43	0.49	-0.15	-0.09	0.17	-0.07	-0.24	-0.09	0.00	-0.20	-0.02	-0.03	-0.01	-0.13	-0.13	-0.21	-0.28	-0.09	-0.25	0.01	0.06	0.20	0.03	0.19	0.08	0.05	0.02	0.23	0.05
OTU0210	-6.69	0.59	0.68	0.73	0.16	-0.10	0.37	-0.11	-0.35	-0.10	-0.03	0.07	-0.15	-0.16	-0.06	-0.06	-0.09	-0.11	-0.25	-0.01	0.12	-0.07	-0.17	-0.14	0.03	-0.32	-0.04	0.16	0.04	0.03	0.00	0.06	0.29	-0.07	0.14	-0.10
OTU0202	-7.09	0.50	0.71	0.53	-0.05	-0.35	0.46	-0.40	-0.38	-0.05	-0.15	0.23	-0.20	-0.21	0.08	-0.05	-0.08	-0.01	-0.14	-0.08	0.10	-0.18	-0.06	-0.12	0.16	-0.22	0.07	-0.06	0.03	0.04	0.07	0.08	0.10	0.02	0.11	-0.17
OTU0137	-5.06	-1.58	0.13	-1.15	0.72	-0.40	0.42	-0.04	0.30	-0.25	-0.44	0.14	-0.16	-0.22	-0.07	-0.04	-0.14	-0.03	-0.30	-0.22	0.16	-0.30	0.16	-0.21	-0.07	-0.14	-0.05	-0.05	0.18	0.11	0.08	0.06	0.18	-0.14	-0.04	-0.13
OTU0047	-8.49	1.16	1.28	1.19	0.06	-0.11	0.55	-0.75	-0.84	-0.01	-0.10	0.16	-0.17	-0.26	0.01	-0.11	-0.01	-0.11	-0.29	-0.06	-0.06	-0.14	0.04	-0.27	-0.01	-0.16	0.11	0.07	0.03	0.03	0.07	0.02	0.02	-0.09	0.07	-0.16
OTU0191	-11.40	0.52	2.48	2.76	0.25	-0.11	0.64	-0.33	-0.52	-0.14	-0.10	0.16	0.23	-0.25	0.14	0.08	-0.17	-0.13	-0.11	0.28	-0.03	0.12	-0.03	-0.12	0.15	-0.12	-0.07	-0.06	-0.06	0.01	-0.12	0.05	0.11	-0.25	0.08	-0.02
OTU0089	-11.30	0.58	2.36	2.45	0.48	0.04	0.64	0.09	-0.02	-0.19	-0.05	0.34	0.23	-0.21	0.15	0.05	-0.17	-0.04	-0.01	0.33	0.00	0.11	-0.05	-0.11	0.11	-0.21	-0.03	-0.04	-0.01	0.01	-0.06	0.10	0.07	-0.18	0.15	0.04
OTU0068	-10.66	-0.08	1.98	1.83	0.24	-0.10	0.68	0.36	0.33	-0.22	-0.02	0.23	0.14	-0.29	0.10	-0.01	-0.23	-0.03	-0.06	0.23	-0.05	0.01	-0.09	-0.15	0.06	-0.14	-0.09	-0.05	0.04	0.00	-0.06	0.03	0.10	-0.13	0.20	-0.02
OTU0034	-11.29	0.10	2.23	2.23	0.50	0.04	0.70	0.50	0.45	-0.18	-0.08	0.36	0.20	-0.26	0.16	-0.02	-0.22	-0.06	0.01	0.26	0.01	0.06	-0.03	-0.15	0.10	-0.22	-0.08	-0.05	0.09	-0.06	-0.03	0.10	0.05	-0.05	0.22	0.00
OTU0031	-11.52	0.21	2.33	2.35	0.50	-0.04	0.73	0.20	0.15	-0.13	-0.07	0.35	0.19	-0.20	0.17	0.03	-0.27	-0.09	-0.05	0.29	0.05	0.03	0.01	-0.08	0.14	-0.26	-0.06	-0.09	-0.04	-0.04	-0.04	0.05	0.10	-0.08	0.18	-0.07
OTU0167	-6.25	1.35	0.63	1.06	0.50	-0.10	0.31	-0.34	-0.91	0.04	-0.03	0.12	-0.15	-0.14	-0.09	-0.13	-0.17	-0.21	-0.29	-0.03	0.07	-0.11	-0.16	-0.23	-0.12	-0.43	0.07	0.00	0.21	-0.13	0.02	0.24	0.14	0.10	0.06	-0.16
OTU0084	-7.84	1.08	0.97	1.46	0.37	0.10	0.41	-0.02	-0.46	-0.01	-0.09	0.03	-0.12	-0.24	0.00	-0.10	-0.19	-0.19	-0.06	0.04	0.00	-0.06	-0.32	-0.16	-0.07	-0.45	-0.11	0.02	-0.03	-0.13	0.02	0.04	0.08	0.16	0.22	-0.13
OTU0139	-7.66	1.25	0.80	1.09	0.27	0.03	0.42	-0.15	-0.61	0.06	-0.08	-0.01	-0.06	-0.15	-0.14	-0.19	-0.13	-0.26	-0.18	-0.05	-0.09	-0.04	-0.33	-0.32	-0.22	-0.29	0.02	0.09	0.08	-0.10	0.15	0.07	-0.03	0.04	0.22	-0.02
OTU0171	-9.22	1.05	0.78	0.80	0.13	-0.05	0.55	-0.38	-0.58	-0.06	-0.18	0.18	-0.19	-0.10	0.07	0.02	-0.07	-0.06	-0.09	-0.09	0.16	-0.08	-0.18	-0.10	-0.04	-0.33	0.04	-0.01	-0.05	0.03	0.20	0.04	0.00	0.07	0.20	-0.13
OTU0123	-8.32	1.06	0.97	1.03	0.11	-0.24	0.53	-0.58	-0.84	-0.06	-0.10	0.22	-0.24	-0.04	0.04	0.02	-0.11	0.00	-0.15	-0.05	0.15	-0.14	-0.03	-0.16	-0.10	-0.34	0.06	-0.02	-0.05	0.08	0.20	0.01	0.06	-0.07	0.20	-0.09
OTU0119	-7.81	0.85	0.56	0.75	-0.23	0.19	0.43	0.28	-0.04	-0.22	-0.12	0.14	-0.05	-0.10	-0.02	0.13	-0.15	-0.15	-0.09	-0.04	0.12	0.10	-0.24	-0.29	-0.01	-0.36	0.04	0.19	0.23	0.01	-0.04	0.10	0.09	0.04	0.38	-0.13
OTU0110	-7.83	1.04	0.69	0.85	-0.02	0.01	0.44	0.06	-0.26	-0.22	-0.12	0.17	-0.04	-0.01	-0.01	0.11	-0.12	-0.16	-0.10	-0.08	0.09	0.09	-0.16	-0.25	0.02	-0.36	0.08	0.18	0.17	0.07	-0.03	0.06	0.12	-0.05	0.37	-0.12
OTU0099	-7.87	1.88	1.70	2.01	0.27	0.11	0.36	-1.20	-1.56	-0.14	-0.06	0.74	0.18	0.31	0.20	0.45	-0.11	0.08	-0.15	0.10	0.78	0.07	0.13	0.14	0.42	-0.40	0.23	-0.04	0.07	0.01	0.00	0.02	0.07	0.07	0.15	-0.07
OTU0062	-8.72	2.25	1.16	1.92	0.25	0.45	0.38	-0.67	-1.02	-0.12	-0.02	0.65	0.12	0.27	0.10	0.31	-0.13	0.01	-0.06	0.05	0.63	0.14	0.01	0.06	0.20	-0.47	0.20	0.03	0.17	-0.04	0.03	0.13	0.13	-0.03	0.37	0.01
OTU0027	-9.59	2.72	1.58	2.31	0.02	0.02	0.53	-1.14	-1.56	-0.07	0.13	0.62	0.00	0.30	0.10	0.22	-0.13	0.08	-0.07	0.08	0.64	-0.02	0.01	0.06	0.08	-0.49	0.23	0.01	0.02	-0.03	0.06	0.04	0.04	0.01	0.41	-0.08
OTU0213	-5.68	0.02	-0.22	-0.43	0.04	0.07	0.33	0.59	0.51	-0.26	-0.24	0.15	-0.13	-0.20	-0.08	-0.03	-0.15	-0.01	-0.12	-0.15	0.07	-0.03	-0.19	-0.31	-0.10	-0.35	0.04	0.04	0.14	0.07	0.15	0.18	0.13	0.02	0.25	-0.01
OTU0138	-6.39	1.04	0.57	0.77	-0.27	-0.28	0.37	-0.17	-0.51	-0.12	-0.06	0.01	-0.22	-0.25	-0.03	-0.03	-0.12	-0.01	-0.03	-0.09	0.10	-0.13	-0.35	-0.23	-0.13	-0.33	0.14	0.03	0.01	0.00	0.13	-0.01	0.02	-0.05	0.31	-0.03
OTU0129	-6.59	-0.46	0.01	-0.12	0.26	-0.07	0.48	0.65	0.69	-0.19	-0.22	0.15	-0.14	-0.13	-0.08	-0.02	-0.20	-0.07	-0.10	-0.08	0.09	-0.12	-0.09	-0.30	-0.13	-0.36	0.03	0.00	0.20	-0.03	0.18	0.12	0.06	-0.02	0.26	0.04
OTU0106	-7.35	1.57	0.60	0.94	-0.16	-0.01	0.40	-0.37	-0.72	-0.09	-0.06	0.11	-0.25	-0.15	-0.07	-0.01	-0.09	-0.03	-0.12	-0.09	0.14	-0.07	-0.33	-0.23	-0.11	-0.39	0.07	0.11	0.00	-0.01	0.16	-0.04	-0.06	0.11	0.25	-0.07
OTU0154	-6.47	1.25	0.85	1.37	-0.03	0.05	0.29	-0.22	-0.40	-0.16	-0.13	0.26	0.07	-0.04	-0.05	0.13	-0.19	-0.13	-0.16	0.06	0.18	0.10	-0.13	-0.23	-0.08	-0.30	0.08	0.09	0.22	-0.03	0.10	0.08	0.05	-0.12	0.24	0.01
OTU0093	-4.54	-3.04	-1.43	-3.18	0.21	-0.13	0.47	1.32	1.96	-0.43	-0.68	-0.20	-0.28	-0.40	-0.20	-0.14	-0.16	-0.05	-0.26	-0.49	-0.15	-0.23	-0.03	-0.43	-0.30	-0.17	-0.02	0.03	0.38	0.05	0.20	0.23	0.13	-0.04	0.00	0.03
OTU0045	-4.41	1.06	0.74	0.70	0.22	-0.50	0.30	-0.91	-1.08	-0.13	-0.11	-0.03	-0.32	-0.06	-0.01	-0.07	-0.12	0.06	-0.18	-0.14	0.02	-0.32	-0.21	-0.18	-0.27	-0.31	0.13	-0.11	0.01	-0.03	0.17	-0.12	-0.01	-0.09	0.07	-0.08
OTU0022	-8.95	0.39	0.74	0.76	0.18	0.11	0.64	0.14	0.04	-0.11	-0.03	0.23	-0.12	-0.09	0.01	-0.04	-0.24	-0.03	-0.10	-0.01	0.12	-0.14	-0.09	-0.20	-0.15	-0.42	0.04	-0.01	0.05	-0.11	0.11	0.01	0.06	0.11	0.34	-0.01
OTU0147	-7.93	1.24	0.24	0.64	-0.10	0.28	0.42	0.18	-0.16	-0.14	0.00	0.10	-0.19	-0.25	0.08	-0.16	-0.18	-0.15	-0.11	-0.05	0.02	-0.03	-0.28	-0.30	-0.15	-0.28	0.06	0.13	0.15	-0.12	-0.01	0.21	0.11	0.13	0.35	-0.02
OTU0019	-8.25	1.94	0.85	1.56	0.14	0.11	0.47	-0.06	-0.50	-0.06	0.14	0.29	-0.16	-0.20	0.16	-0.14	-0.24	-0.11	-0.02	0.07	0.10	-0.16	-0.30	-0.22	-0.06	-0.38	0.07	0.09	0.06	-0.14	0.07	0.06	0.02	0.32	0.37	-0.08
OTU0169	-9.33	1.63	2.44	3.02	0.06	-0.02	0.43	-1.10	-1.28	-0.21	0.15	0.25	0.17	-0.04	0.09	0.26	-0.03	-0.03	-0.21	0.29	0.13	0.01	-0.04	0.00	0.15	-0.07	0.12	-0.12	-0.09	0.00	-0.12	0.02	0.06	-0.12	0.12	-0.03
OTU0126	-7.06	1.49	2.18	1.66	0.07	-0.52	0.43	-2.24	-2.15	-0.15	0.08	-0.07	-0.38	-0.28	-0.04	-0.10	0.02	0.11	-0.31	0.03	-0.28	-0.35	-0.11	-0.19	-0.17	0.04	0.07	-0.25	-0.26	0.02	0.10	-0.12	-0.09	-0.08	-0.01	-0.13
OTU0200	-7.92	1.40	1.19	2.11	0.21	0.12	0.33	-0.53	-0.62	-0.16	-0.05	0.02	0.16	-0.07	-0.19	0.14	-0.12	-0.06	-0.15	0.12	0.04	0.03	-0.24	-0.06	0.12	-0.26	-0.01	-0.03	0.00	-0.01	0.05	0.16	0.14	-0.15	0.11	-0.09
OTU0122	-6.57	0.21	0.41	0.62	0.22	-0.15	0.43	0.18	0.25	-0.26	-0.08	0.00	-0.06	-0.17	-0.28	-0.03	-0.21	0.00	-0.11	-0.07	-0.01	-0.24	-0.23	-0.18	-0.16	-0.29	-0.05	0.03	0.10	0.03	0.06	0.12	0.07	-0.03	0.23	-0.15
OTU0221	-5.73	1.59	1.42	2.08	0.01	-0.15	0.25	-1.66	-2.19	0.11	0.02	0.01	-0.14	0.00	-0.21	-0.07	-0.19	-0.14	-0.36	0.02	0.13	-0.08	-0.09	-0.05	-0.11	-0.21	-0.06	0.01	-0.08	-0.05	-0.11	-0.02	0.12	-0.06	-0.11	-0.27

OTU	(Intercept)	Log type: natural	Year: 2020	Year: 2021	Mortality factor: uprooted	Decay stage	Sequencing depth	Log type: natural x Year: 2020	Log type: natural x Year: 2021	Year: 2020 x *Antrodia piceata*	Year: 2020 x *Antrodiella citrinella*	Year: 2020 x *Fomitopsis rosea*	Year: 2020 x *Perenniporia subacida*	Year: 2020 x *Physisporinus crocatus*	Year: 2020 x *Postia guttulata*	Year: 2020 x *Skeletocutis odora*	Year: 2020 x *Skeletocutis stellae*	Year: 2020 x *Steccherinum collabens*	Year: 2021 x *Antrodia piceata*	Year: 2021 x *Antrodiella citrinella*	Year: 2021 x *Fomitopsis rosea*	Year: 2021 x *Perenniporia subacida*	Year: 2021 x *Physisporinus crocatus*	Year: 2021 x *Postia guttulata*	Year: 2021 x *Skeletocutis odora*	Year: 2021 x *Skeletocutis stellae*	Year: 2021 x *Steccherinum collabens*	Log type: natural x *Antrodia piceata*	Log type: natural x *Antrodiella citrinella*	Log type: natural x. *Fomitopsis rosea*	Log type: natural x *Perenniporia subacida*	Log type: natural x *Physisporinus crocatus*	Log type: natural x *Postia guttulata*	Log type: natural x *Skeletocutis odora*	Log type: natural x *Skeletocutis stellae*	Log type: natural x *Steccherinum collabens*
OTU0179	-5.25	-2.01	-1.09	-2.35	0.28	-0.05	0.43	0.58	0.99	-0.38	-0.59	-0.22	-0.26	-0.41	-0.31	-0.07	-0.09	-0.06	-0.32	-0.36	-0.20	-0.21	-0.19	-0.34	-0.20	-0.17	-0.07	-0.02	0.17	0.03	0.03	0.09	0.14	-0.08	-0.12	-0.12
OTU0080	-5.56	1.79	0.09	0.95	0.64	0.03	0.24	-0.41	-1.02	-0.02	-0.12	-0.08	-0.24	-0.17	-0.26	-0.19	-0.20	-0.14	-0.24	-0.06	-0.18	-0.11	-0.44	-0.27	-0.30	-0.44	-0.07	0.14	0.02	0.03	0.02	-0.04	0.13	-0.14	0.19	-0.12
OTU0220	-3.77	0.01	0.18	0.11	0.57	0.08	0.15	-0.17	-0.38	-0.12	-0.27	-0.09	-0.17	-0.27	-0.27	-0.01	-0.21	-0.07	-0.24	-0.10	-0.18	-0.07	-0.20	-0.32	-0.15	-0.23	0.05	0.12	0.08	-0.07	0.10	-0.03	0.03	0.13	0.02	-0.01
OTU0036	-3.83	0.10	-0.31	-0.44	1.03	-0.21	0.30	-0.02	0.09	-0.34	-0.17	0.06	-0.22	0.02	-0.28	-0.10	-0.16	-0.04	-0.23	-0.14	-0.18	-0.20	-0.09	-0.37	-0.14	-0.30	-0.05	0.00	0.30	-0.06	0.04	0.01	0.03	0.06	0.19	-0.16
OTU0035	-5.69	0.58	0.70	0.68	0.23	-0.28	0.40	-1.10	-1.43	-0.10	0.00	0.00	-0.38	-0.16	-0.16	-0.20	-0.11	-0.03	-0.30	-0.04	0.07	-0.26	-0.16	-0.16	-0.30	-0.18	-0.07	-0.10	0.08	0.02	0.03	-0.06	0.02	-0.08	0.00	-0.23
OTU0029	-8.40	2.02	2.50	3.07	0.20	-0.34	0.52	-2.30	-2.71	-0.15	0.29	-0.05	-0.05	0.04	-0.14	0.08	-0.18	-0.05	-0.42	0.27	0.02	-0.16	-0.05	-0.13	-0.21	-0.20	-0.09	-0.15	-0.11	0.10	-0.09	-0.26	-0.10	-0.33	-0.05	-0.10
OTU0155	-7.46	2.16	1.63	2.26	0.22	-0.02	0.32	-1.50	-1.94	-0.21	-0.01	0.07	0.01	0.06	-0.20	0.51	0.03	0.11	-0.22	0.01	0.21	-0.05	-0.11	-0.07	0.24	-0.23	0.27	0.03	-0.07	0.02	-0.08	0.06	-0.01	-0.40	0.11	-0.04
OTU0079	-3.97	2.06	-0.10	0.73	-0.93	-0.07	0.12	-0.68	-0.91	-0.25	0.00	-0.11	-0.31	-0.18	-0.34	0.05	0.01	0.01	-0.21	-0.11	-0.08	-0.06	-0.33	-0.28	-0.10	-0.29	0.08	0.12	0.21	0.04	0.16	0.07	-0.02	0.06	0.12	-0.08
OTU0018	-3.73	1.07	0.23	0.54	0.37	0.07	0.21	-0.70	-0.81	-0.22	0.01	-0.14	-0.35	-0.05	-0.34	-0.13	-0.17	-0.11	-0.38	-0.11	-0.18	-0.25	-0.05	-0.27	-0.30	-0.27	-0.07	-0.02	0.12	0.02	0.16	0.09	0.08	-0.19	0.16	-0.12
OTU0176	-4.24	-0.04	0.55	0.23	0.26	-0.31	0.27	-0.93	-1.02	-0.27	-0.22	-0.07	-0.26	-0.19	-0.20	-0.17	-0.10	-0.05	-0.39	-0.13	-0.08	-0.34	-0.05	-0.25	-0.32	-0.01	-0.06	0.02	0.04	0.07	0.01	0.00	0.01	-0.10	-0.02	-0.08
OTU0015	-3.84	0.68	1.28	1.04	0.38	-0.57	0.32	-1.67	-1.93	-0.20	0.12	-0.15	-0.43	-0.05	-0.17	-0.30	-0.10	0.00	-0.44	-0.09	-0.21	-0.61	0.00	-0.20	-0.52	-0.07	-0.07	-0.08	-0.15	0.04	0.01	-0.06	-0.03	-0.11	-0.08	-0.10
OTU0131	-5.62	0.47	1.50	0.86	0.50	-0.49	0.34	-1.36	-1.61	-0.05	-0.05	0.32	-0.12	-0.16	-0.04	0.02	-0.12	0.11	-0.11	-0.13	0.20	-0.41	0.15	-0.06	-0.11	-0.16	0.15	-0.09	-0.11	0.06	0.06	-0.17	-0.03	0.01	-0.05	-0.25
OTU0124	-10.21	1.45	1.72	2.09	0.04	0.20	0.55	-0.61	-0.83	-0.04	0.05	0.51	-0.01	-0.15	0.11	-0.06	-0.09	-0.01	0.00	0.17	0.26	-0.08	-0.16	-0.12	0.01	-0.28	0.03	0.01	0.00	-0.13	-0.04	0.00	-0.01	0.13	0.13	-0.02
OTU0092	-4.34	-3.56	-1.48	-3.50	0.35	0.07	0.44	1.60	2.40	-0.38	-0.66	-0.25	-0.28	-0.56	-0.24	-0.22	-0.12	0.00	-0.22	-0.49	-0.25	-0.28	-0.14	-0.45	-0.24	-0.16	0.10	0.04	0.33	0.05	0.12	0.26	0.13	0.12	-0.12	-0.07
OTU0081	-9.45	1.73	1.40	1.71	-0.10	0.21	0.49	-0.48	-0.64	-0.04	-0.04	0.47	-0.07	-0.14	0.14	0.04	-0.11	0.00	-0.03	0.16	0.21	0.02	-0.16	-0.23	0.10	-0.29	0.19	-0.06	0.12	-0.06	0.05	0.08	-0.04	0.18	0.11	0.04
OTU0065	-7.91	-0.45	1.37	1.27	0.37	0.28	0.47	0.45	0.56	-0.05	-0.10	0.30	0.12	-0.17	0.02	-0.08	-0.21	-0.11	-0.16	0.13	0.06	0.06	0.10	-0.23	0.02	-0.24	0.11	0.00	0.28	-0.08	0.06	0.16	-0.02	0.14	0.13	-0.09
OTU0225	-8.53	1.78	0.85	1.25	0.07	0.14	0.40	-0.46	-0.55	-0.10	-0.13	0.47	-0.01	-0.13	0.04	0.02	0.01	0.00	-0.03	0.08	0.20	0.04	-0.17	-0.15	0.18	-0.35	0.03	0.04	0.03	-0.02	0.11	0.01	0.04	0.21	0.23	-0.09
OTU0165	-4.18	-1.21	0.01	-1.09	0.59	-0.29	0.31	0.02	0.29	-0.23	-0.32	0.15	-0.19	-0.24	-0.11	-0.12	-0.07	-0.02	-0.16	-0.26	0.05	-0.32	-0.04	-0.24	-0.13	-0.23	-0.03	0.00	0.11	0.09	0.03	0.01	0.10	0.03	0.01	-0.18
OTU0134	-7.52	1.23	0.53	0.78	-0.02	0.26	0.37	-0.09	-0.21	-0.08	-0.11	0.50	-0.08	-0.15	-0.02	0.03	-0.11	-0.05	-0.05	0.03	0.24	-0.04	-0.20	-0.23	-0.01	-0.39	0.13	0.08	0.19	-0.04	0.09	0.10	0.02	0.18	0.25	-0.04
OTU0193	-6.35	0.89	1.59	1.32	-0.15	-0.22	0.33	-1.02	-1.09	-0.04	-0.09	0.24	-0.07	-0.24	0.03	-0.01	-0.05	0.08	-0.02	0.03	0.00	-0.12	-0.10	-0.17	0.06	-0.19	0.10	-0.03	-0.04	-0.02	0.05	-0.08	-0.01	0.02	-0.05	-0.18
OTU0085	-8.20	1.19	0.77	1.21	0.17	-0.14	0.51	-0.18	-0.40	-0.05	0.10	0.32	-0.09	-0.16	-0.15	-0.08	-0.15	0.00	-0.01	0.02	0.15	-0.15	-0.19	-0.27	-0.02	-0.38	-0.02	0.10	0.19	-0.04	-0.05	-0.02	0.07	0.15	0.27	-0.19
OTU0070	-8.54	0.78	0.78	1.00	0.11	0.12	0.51	0.00	-0.14	-0.09	-0.03	0.45	-0.07	-0.15	-0.04	-0.06	-0.11	-0.01	-0.06	0.03	0.16	-0.10	-0.18	-0.10	0.02	-0.29	0.05	0.02	0.21	-0.08	0.00	-0.04	0.15	0.13	0.15	-0.13
OTU0030	-7.69	1.37	1.78	1.72	0.17	-0.25	0.49	-1.19	-1.10	-0.14	0.07	0.62	-0.04	0.05	0.06	0.15	0.01	0.22	0.04	0.05	0.30	-0.22	0.12	-0.01	0.21	-0.13	0.18	-0.12	-0.01	-0.01	0.08	-0.20	0.02	0.15	0.18	-0.19
OTU0151	-10.52	1.79	2.42	2.69	0.09	-0.36	0.58	-1.16	-1.45	0.25	0.04	0.51	-0.05	-0.18	0.23	-0.07	-0.11	-0.08	-0.07	0.30	0.14	-0.10	-0.03	-0.08	-0.09	-0.07	0.21	-0.12	0.00	0.05	-0.02	0.04	-0.01	-0.05	0.06	-0.09
OTU0014	-8.93	1.86	2.08	2.17	-0.05	-0.26	0.56	-1.40	-1.44	-0.14	0.10	0.79	-0.01	0.10	0.21	0.21	-0.11	0.37	0.00	0.15	0.53	-0.22	0.23	0.08	0.15	-0.25	0.39	-0.14	0.02	0.03	0.10	-0.12	-0.09	0.13	0.17	-0.10
OTU0190	-12.39	1.97	1.74	2.54	0.20	0.11	0.65	-0.59	-0.89	0.01	0.02	0.19	0.15	-0.14	-0.04	0.06	-0.02	-0.17	-0.12	0.23	0.07	0.20	-0.17	-0.22	0.10	-0.28	0.05	0.12	-0.02	0.03	0.00	0.13	-0.01	-0.05	0.24	-0.06
OTU0023	-7.56	1.01	2.11	1.95	0.28	-0.20	0.47	-1.31	-1.19	-0.23	0.19	0.53	0.05	0.14	0.01	0.28	0.03	0.26	-0.16	0.08	0.35	-0.27	0.25	0.07	0.41	-0.04	0.27	-0.13	-0.05	0.07	-0.02	-0.03	0.07	0.07	0.09	-0.18
OTU0009	-7.68	1.86	2.60	2.71	0.21	-0.56	0.51	-1.65	-1.71	-0.03	0.30	0.19	-0.18	0.00	-0.08	-0.08	-0.10	-0.03	-0.13	0.17	-0.14	-0.38	0.02	-0.17	0.00	-0.15	0.13	-0.06	-0.32	0.01	-0.01	-0.18	-0.02	0.10	0.13	-0.26
OTU0219	-7.10	1.80	0.28	0.97	-0.22	-0.01	0.29	-0.14	-0.35	-0.20	0.02	0.20	0.01	-0.04	-0.09	0.10	0.02	0.00	-0.05	0.04	0.22	-0.04	-0.24	-0.14	0.26	-0.30	-0.06	0.24	0.15	0.00	-0.01	0.15	0.11	0.12	0.36	-0.10
OTU0184	-4.99	-2.27	-0.46	-1.97	0.45	-0.18	0.41	0.85	1.45	-0.53	-0.41	0.01	-0.09	-0.09	-0.13	0.01	-0.01	0.10	-0.24	-0.28	-0.05	-0.23	0.16	-0.30	0.14	-0.01	-0.08	0.06	0.17	0.06	0.12	0.15	0.17	-0.04	-0.01	-0.03
OTU0112	-4.51	-0.86	0.39	-0.47	0.74	-0.46	0.34	-0.13	-0.17	-0.32	-0.14	0.33	-0.02	0.02	-0.20	0.00	-0.21	0.14	-0.27	-0.12	0.21	-0.38	0.26	-0.15	-0.06	-0.18	-0.12	0.05	0.07	0.14	0.08	-0.02	0.16	-0.05	-0.03	-0.14
OTU0105	-5.55	-3.03	-0.41	-2.61	0.66	-0.05	0.49	0.78	1.47	-0.38	-0.34	-0.13	-0.10	-0.42	-0.21	-0.15	-0.09	-0.02	-0.30	-0.36	-0.26	-0.34	0.08	-0.40	-0.25	0.02	-0.13	0.01	0.14	0.13	0.11	0.15	0.14	-0.10	-0.13	0.00
OTU0182	-9.14	2.23	1.21	1.92	-0.02	0.04	0.42	-0.60	-1.04	-0.06	0.15	0.84	0.07	0.08	0.02	0.15	-0.16	0.13	0.05	0.18	0.63	-0.04	-0.05	0.02	0.12	-0.42	-0.06	0.11	0.10	-0.03	-0.01	0.03	-0.03	0.09	0.21	0.06
OTU0172	-9.48	1.88	1.36	1.80	0.19	0.04	0.45	-0.64	-1.00	-0.08	0.15	1.08	0.12	0.14	0.06	0.22	-0.11	0.21	0.04	0.16	0.87	-0.03	0.11	0.11	0.18	-0.38	-0.02	0.01	0.14	-0.02	0.00	0.09	0.00	0.00	0.28	0.06
OTU0076	-7.86	2.12	1.49	1.91	0.00	-0.20	0.39	-0.92	-1.21	-0.11	0.19	0.98	0.01	0.12	0.10	0.16	-0.20	0.25	0.18	0.15	0.72	-0.17	0.01	0.11	0.09	-0.38	-0.04	-0.01	0.00	-0.02	0.03	-0.08	-0.03	0.06	0.32	-0.02
OTU0044	-8.52	1.57	1.14	1.60	0.20	0.00	0.46	0.02	-0.17	-0.15	0.27	1.13	0.12	0.17	-0.02	0.29	-0.37	0.19	0.02	0.23	0.93	-0.01	0.05	-0.02	0.04	-0.45	0.03	0.10	0.11	-0.06	0.12	0.05	0.09	0.19	0.32	0.15
OTU0121	-3.13	-2.72	-1.52	-3.05	0.05	-0.05	0.33	0.97	1.58	-0.47	-0.51	-0.12	-0.17	-0.34	-0.31	-0.17	-0.08	0.04	-0.32	-0.42	-0.11	-0.30	-0.02	-0.34	-0.29	-0.05	-0.12	0.08	0.40	0.08	0.10	0.07	0.22	0.08	-0.04	-0.09
OTU0187	-5.03	-1.41	0.25	-0.84	0.51	-0.16	0.35	-0.07	0.38	-0.25	-0.35	0.08	-0.03	-0.19	-0.08	-0.08	-0.16	-0.03	-0.27	-0.10	0.05	-0.19	0.15	-0.19	-0.22	-0.06	-0.03	-0.13	0.12	0.03	0.17	-0.01	0.20	-0.02	-0.13	-0.05
OTU0163	-6.28	-3.37	-1.56	-3.47	0.44	-0.06	0.55	1.36	2.04	-0.49	-0.71	-0.02	-0.08	-0.30	-0.28	-0.10	-0.11	0.08	-0.25	-0.45	0.06	-0.18	0.08	-0.30	-0.16	-0.07	-0.11	-0.02	0.32	0.11	0.13	0.18	0.34	-0.03	-0.11	-0.01
OTU0136	-4.50	-1.78	-0.42	-1.61	0.47	-0.36	0.40	0.22	0.58	-0.23	-0.39	0.10	-0.14	-0.18	-0.17	-0.10	-0.23	-0.01	-0.21	-0.24	0.14	-0.32	0.10	-0.20	-0.29	-0.12	0.00	-0.10	0.10	0.03	0.14	-0.03	0.19	0.02	-0.07	-0.03
OTU0133	-8.69	0.94	1.40	1.42	-0.19	-0.02	0.47	-0.41	-0.30	-0.33	-0.01	0.83	0.32	0.09	0.09	0.48	-0.10	0.23	-0.05	0.16	0.71	0.20	0.23	0.03	0.37	-0.06	0.09	-0.02	0.16	0.01	0.17	-0.03	0.15	0.09	0.20	0.07
OTU0116	-4.80	-2.43	-0.93	-2.21	0.29	-0.30	0.45	0.88	1.27	-0.29	-0.54	-0.02	-0.13	-0.28	-0.26	-0.22	-0.22	-0.03	-0.28	-0.25	0.04	-0.28	0.08	-0.33	-0.35	-0.12	-0.06	0.03	0.21	0.05	0.15	0.04	0.23	0.03	-0.03	-0.02
OTU0111	-2.66	-1.44	-0.06	-0.92	0.44	-0.31	0.23	0.05	0.13	-0.19	-0.26	0.06	-0.24	-0.17	-0.20	-0.11	-0.20	-0.04	-0.26	-0.13	0.08	-0.35	0.11	-0.20	-0.37	-0.15	-0.11	-0.13	0.11	0.10	0.13	-0.07	0.21	-0.07	-0.02	-0.13
OTU0078	-3.09	-0.49	0.52	-0.13	0.46	-0.29	0.22	-0.43	-0.44	-0.02	-0.16	0.13	-0.22	-0.08	-0.18	-0.07	-0.22	0.00	-0.22	-0.07	0.18	-0.34	0.12	-0.14	-0.31	-0.16	-0.08	-0.15	0.07	0.07	0.18	-0.14	0.15	-0.03	0.02	-0.15
OTU0073	-3.55	-2.11	-0.61	-2.07	0.25	-0.19	0.35	0.56	1.06	-0.34	-0.43	0.15	-0.13	-0.22	-0.14	-0.09	-0.23	0.11	-0.25	-0.31	0.12	-0.34	0.16	-0.16	-0.23	-0.13	-0.08	-0.08	0.16	0.06	0.16	0.03	0.26	0.08	-0.06	-0.13
OTU0060	-3.28	-2.08	-0.74	-1.94	0.51	-0.51	0.37	0.67	0.87	-0.18	-0.37	0.11	-0.24	-0.20	-0.24	-0.28	-0.33	0.07	-0.21	-0.25	0.29	-0.41	0.09	-0.19	-0.37	-0.27	-0.15	-0.05	0.07	0.10	0.16	-0.04	0.28	0.11	-0.02	-0.14
OTU0053	-4.06	-1.84	0.14	-1.28	0.59	-0.48	0.40	0.05	0.30	-0.17	-0.31	0.06	-0.22	-0.22	-0.15	-0.29	-0.25	0.00	-0.22	-0.23	0.22	-0.46	0.15	-0.15	-0.35	-0.16	-0.14	-0.12	-0.04	0.09	0.20	-0.05	0.18	0.09	-0.11	-0.17
OTU0183	-4.89	-2.45	-0.76	-2.14	0.31	-0.22	0.42	0.66	1.20	-0.41	-0.57	-0.28	-0.13	-0.39	-0.13	-0.13	-0.10	0.00	-0.24	-0.33	-0.08	-0.26	0.01	-0.28	-0.25	0.04	-0.13	-0.07	0.16	0.07	0.18	0.09	0.23	-0.04	-0.10	-0.05
OTU0125	-6.07	-3.19	-1.02	-2.90	0.33	-0.16	0.56	1.03	1.85	-0.52	-0.62	-0.27	-0.08	-0.43	-0.15	-0.08	-0.08	0.08	-0.25	-0.41	-0.10	-0.26	0.07	-0.27	-0.20	0.04	-0.12	-0.09	0.20	0.09	0.19	0.11	0.26	0.00	-0.10	-0.02
OTU0088	-3.85	-2.97	-1.32	-3.14	0.30	-0.17	0.42	1.03	1.82	-0.45	-0.55	-0.25	-0.24	-0.36	-0.16	-0.19	-0.09	0.08	-0.23	-0.45	-0.10	-0.33	0.03	-0.32	-0.29	-0.06	-0.11	-0.10	0.22	0.06	0.29	0.11	0.22	0.05	-0.02	-0.07
OTU0066	-1.54	-2.11	-1.45	-2.95	0.40	-0.36	0.25	0.66	1.29	-0.37	-0.44	-0.30	-0.42	-0.24	-0.22	-0.33	-0.13	0.14	-0.23	-0.45	-0.11	-0.48	-0.02	-0.29	-0.31	-0.16	-0.05	-0.13	0.10	0.14	0.29	-0.01	0.15	0.12	0.11	-0.11
OTU0095	-4.91	-2.93	-1.36	-3.21	0.38	-0.22	0.51	1.08	1.85	-0.55	-0.62	-0.13	-0.22	-0.29	-0.17	-0.09	-0.04	0.12	-0.23	-0.46	0.01	-0.33	0.10	-0.22	-0.20	-0.08	-0.10	-0.09	0.22	0.12	0.16	0.06	0.27	0.00	-0.01	0.00
OTU0040	-4.24	-2.66	-0.65	-2.35	0.74	-0.43	0.48	0.48	1.10	-0.39	-0.44	-0.10	-0.22	-0.21	-0.20	-0.16	-0.18	0.05	-0.27	-0.36	0.04	-0.47	0.19	-0.22	-0.33	-0.10	-0.13	-0.13	0.11	0.11	0.16	-0.01	0.27	-0.05	-0.07	-0.07
OTU0038	-4.26	-2.82	-0.95	-2.76	0.04	-0.20	0.49	1.16	2.05	-0.41	-0.44	-0.20	-0.29	-0.42	-0.17	-0.29	-0.22	-0.04	-0.16	-0.39	-0.28	-0.42	-0.09	-0.42	-0.38	-0.16	-0.10	-0.05	0.19	0.01	0.27	0.06	0.09	0.18	-0.07	0.00
OTU0192	-6.95	2.27	1.82	2.74	0.40	-0.38	0.27	-1.23	-1.63	-0.13	0.34	0.13	0.14	-0.02	-0.16	0.06	-0.18	-0.05	-0.21	0.15	0.02	-0.06	0.03	-0.16	-0.06	-0.18	-0.16	-0.06	-0.09	0.11	-0.10	-0.03	0.09	-0.21	0.07	-0.02
OTU0168	-6.84	2.10	1.23	1.95	0.15	-0.38	0.30	-0.70	-1.02	-0.14	0.22	0.13	0.06	-0.20	-0.12	-0.08	-0.18	-0.04	-0.13	0.02	-0.06	-0.10	-0.26	-0.21	-0.18	-0.20	-0.06	0.10	-0.02	0.02	0.01	-0.11	0.11	-0.09	0.09	-0.06
OTU0199	-8.99	1.02	2.24	2.52	0.56	-0.14	0.43	-0.62	-0.49	-0.19	0.00	0.26	0.28	-0.13	0.22	0.08	-0.18	-0.14	-0.07	0.27	-0.13	0.05	0.18	-0.21	0.15	-0.07	0.03	0.02	-0.07	-0.02	0.02	0.03	0.06	-0.16	0.08	0.04
OTU0114	-8.89	1.32	2.76	2.96	0.11	-0.26	0.45	-1.22	-1.42	-0.17	0.20	0.26	0.22	-0.04	0.16	0.05	-0.17	-0.09	-0.18	0.36	0.01	-0.16	0.08	-0.15	0.02	-0.07	-0.02	-0.04	-0.11	0.03	0.09	-0.05	-0.03	-0.17	0.04	-0.03
OTU0108	-8.60	0.75	2.25	2.31	0.45	-0.01	0.44	-0.39	-0.27	-0.20	-0.01	0.30	0.24	-0.18	0.18	-0.05	-0.29	-0.08	-0.07	0.30	-0.04	-0.06	0.12	-0.23	0.10	-0.10	0.09	0.08	-0.05	-0.01	0.08	0.02	0.00	-0.04	0.04	0.03
OTU0083	-6.49	1.46	0.71	1.48	0.18	-0.17	0.35	-0.30	-0.60	-0.19	0.10	0.04	-0.03	-0.17	-0.15	-0.20	-0.25	-0.15	-0.13	0.04	-0.12	-0.22	-0.15	-0.34	-0.27	-0.31	-0.13	0.20	0.07	-0.04	0.04	-0.06	0.11	0.09	0.29	-0.12
OTU0046	-10.14	0.40	1.01	0.77	0.34	0.01	0.71	-0.22	-0.05	-0.33	0.05	0.30	-0.05	-0.18	0.02	-0.05	-0.29	0.02	-0.10	0.08	0.03	-0.27	0.14	-0.27	-0.12	-0.23	-0.02	0.06	0.06	-0.05	0.14	-0.09	0.14	0.07	0.20	0.03
OTU0033	-10.29	1.15	0.38	1.21	0.03	0.02	0.69	0.10	-0.23	-0.16	0.13	0.16	-0.10	-0.13	-0.15	-0.19	-0.34	-0.13	-0.12	0.05	0.02	-0.19	-0.13	-0.30	-0.33	-0.38	-0.18	0.19	0.04	-0.09	0.12	0.15	0.12	0.05	0.32	-0.16
OTU0206	-6.46	-0.07	0.59	0.40	0.52	-0.18	0.39	-0.38	-0.14	-0.30	-0.21	0.00	0.01	-0.02	-0.13	0.04	-0.02	-0.03	-0.33	-0.05	-0.14	-0.16	0.27	-0.28	0.16	-0.02	-0.02	-0.01	0.07	0.12	0.03	0.00	0.05	-0.26	0.09	-0.04
OTU0141	-7.23	-0.30	-0.12	-0.39	0.19	0.09	0.52	0.36	0.54	-0.26	-0.15	0.23	-0.04	-0.12	-0.16	0.01	-0.15	0.04	-0.16	-0.03	-0.06	-0.21	0.09	-0.33	0.04	-0.18	-0.08	0.06	0.19	-0.02	0.06	0.02	0.10	0.01	0.19	0.02
OTU0150	-8.24	-0.32	0.36	0.13	0.30	0.08	0.56	0.34	0.55	-0.20	-0.11	0.24	-0.06	-0.14	-0.09	-0.14	-0.23	0.02	-0.16	0.10	0.09	-0.12	0.10	-0.24	-0.05	-0.33	-0.03	0.06	0.04	0.07	0.19	0.07	0.10	0.11	0.21	0.01
OTU0135	-7.78	0.69	1.69	1.36	0.37	-0.37	0.52	-1.37	-1.25	-0.17	0.09	0.04	-0.08	0.05	0.03	-0.15	-0.17	-0.04	-0.25	0.21	-0.04	-0.37	0.19	-0.11	-0.16	-0.12	-0.08	-0.10	-0.24	0.07	0.13	-0.06	0.09	-0.18	0.00	-0.17
OTU0216	-6.12	-0.77	-0.11	-0.33	0.47	-0.09	0.42	0.61	0.79	-0.15	-0.30	0.04	0.00	-0.05	-0.14	-0.13	-0.30	-0.17	-0.25	0.05	-0.05	-0.14	0.11	-0.30	-0.17	-0.17	-0.19	0.04	0.26	-0.03	0.12	0.18	0.16	-0.05	0.19	-0.13
OTU0143	-5.97	0.02	0.90	0.64	0.58	-0.40	0.40	-0.61	-0.67	-0.20	-0.04	-0.07	-0.05	-0.10	-0.12	-0.09	-0.25	-0.09	-0.26	0.09	-0.18	-0.35	0.19	-0.29	-0.14	-0.16	-0.11	0.03	-0.03	0.02	0.09	0.06	0.06	-0.25	0.07	-0.06
OTU0104	-7.33	-0.58	-0.24	-0.37	0.22	0.00	0.53	0.68	0.73	-0.20	-0.18	-0.05	-0.05	-0.27	-0.19	-0.20	-0.19	-0.09	-0.23	0.01	-0.16	-0.18	-0.10	-0.36	-0.22	-0.23	-0.10	0.14	0.17	0.03	0.15	0.16	0.05	0.05	0.12	0.01
OTU0102	-7.27	-0.33	0.14	0.36	0.43	-0.01	0.50	0.47	0.49	-0.12	-0.17	0.32	0.05	-0.05	-0.08	-0.20	-0.30	-0.08	-0.22	0.00	0.12	-0.15	0.13	-0.26	-0.18	-0.21	-0.12	0.02	0.22	-0.05	0.19	0.14	0.13	-0.01	0.19	0.05
OTU0074	-8.21	-0.73	0.62	0.62	0.52	0.07	0.57	0.92	0.88	-0.11	-0.04	0.10	0.09	-0.10	-0.15	-0.18	-0.37	-0.20	-0.21	0.14	-0.14	-0.10	0.12	-0.27	-0.04	-0.14	-0.13	0.02	0.30	-0.14	0.07	0.21	0.18	0.07	0.20	-0.07
OTU0173	-5.40	-0.60	-0.33	-0.05	0.35	0.12	0.35	0.80	0.67	-0.22	-0.16	0.19	0.00	0.06	-0.31	-0.05	-0.27	-0.10	-0.23	-0.06	0.01	-0.04	0.25	-0.24	-0.04	-0.17	-0.09	0.11	0.46	0.07	0.07	0.20	0.19	0.00	0.19	-0.12
OTU0041	-6.81	-0.60	0.03	-0.01	0.50	0.02	0.52	0.78	0.63	-0.25	-0.07	0.20	-0.01	0.03	-0.20	-0.11	-0.32	-0.03	-0.30	0.00	-0.09	-0.14	0.29	-0.33	-0.08	-0.21	-0.14	-0.02	0.35	0.03	0.07	0.18	0.20	0.03	0.21	-0.12
OTU0212	-6.21	0.75	1.13	1.17	0.35	-0.26	0.33	-0.30	-0.40	-0.15	0.08	0.12	0.01	-0.08	-0.11	-0.04	-0.15	-0.09	-0.14	0.13	0.01	-0.16	0.02	-0.20	-0.06	-0.14	-0.02	0.04	0.03	-0.04	0.10	0.09	0.09	-0.11	0.17	-0.06
OTU0039	-6.88	1.61	1.59	1.89	0.25	-0.31	0.36	-0.53	-0.88	-0.13	0.21	0.14	0.05	0.12	-0.08	-0.05	-0.13	-0.08	-0.13	0.17	0.08	-0.17	0.10	-0.22	0.03	-0.19	-0.02	0.07	0.02	0.02	0.12	0.10	0.12	-0.15	0.31	-0.10
OTU0215	-7.59	0.34	0.72	0.90	0.26	-0.03	0.44	0.19	0.19	-0.25	0.06	0.09	0.10	-0.12	-0.20	-0.04	-0.19	-0.08	-0.25	0.07	-0.13	-0.05	0.00	-0.30	0.02	-0.18	-0.07	0.02	0.06	0.06	0.05	0.15	0.08	-0.12	0.29	0.06

OTU	(Intercept)	Log type: natural	Year: 2020	Year: 2021	Mortality factor: uprooted	Decay stage	Sequencing depth	Log type: natural x Year: 2020	Log type: natural x Year: 2021	Year: 2020 x *Antrodia piceata*	Year: 2020 x *Antrodiella citrinella*	Year: 2020 x *Fomitopsis rosea*	Year: 2020 x *Perenniporia subacida*	Year: 2020 x *Physisporinus crocatus*	Year: 2020 x *Postia guttulata*	Year: 2020 x *Skeletocutis odora*	Year: 2020 x *Skeletocutis stellae*	Year: 2020 x *Steccherinum collabens*	Year: 2021 x *Antrodia piceata*	Year: 2021 x *Antrodiella citrinella*	Year: 2021 x *Fomitopsis rosea*	Year: 2021 x *Perenniporia subacida*	Year: 2021 x *Physisporinus crocatus*	Year: 2021 x *Postia guttulata*	Year: 2021 x *Skeletocutis odora*	Year: 2021 x *Skeletocutis stellae*	Year: 2021 x *Steccherinum collabens*	Log type: natural x *Antrodia piceata*	Log type: natural x *Antrodiella citrinella*	Log type: natural x. *Fomitopsis rosea*	Log type: natural x *Perenniporia subacida*	Log type: natural x *Physisporinus crocatus*	Log type: natural x *Postia guttulata*	Log type: natural x *Skeletocutis odora*	Log type: natural x *Skeletocutis stellae*	Log type: natural x *Steccherinum collabens*
OTU0075	-8.81	-0.22	0.04	0.20	0.28	-0.15	0.64	0.82	0.93	-0.41	-0.04	0.05	0.06	-0.14	-0.25	-0.02	-0.25	-0.04	-0.23	0.06	-0.09	-0.04	-0.06	-0.41	0.00	-0.15	-0.10	0.05	0.23	0.07	0.00	0.10	0.20	-0.10	0.32	0.02
OTU0130	-9.31	0.72	1.27	1.40	0.23	0.01	0.54	-0.35	-0.50	-0.22	-0.04	0.12	0.11	-0.15	-0.01	-0.02	-0.22	-0.08	-0.31	0.13	-0.09	-0.05	0.01	-0.26	0.01	-0.12	-0.03	0.09	0.01	0.04	0.06	0.04	0.08	-0.14	0.05	-0.01
OTU0050	-9.22	0.72	1.06	1.09	0.11	-0.13	0.62	-0.17	-0.31	-0.30	0.02	0.11	0.01	-0.13	-0.06	-0.04	-0.15	-0.04	-0.33	0.13	-0.19	-0.05	0.06	-0.35	-0.03	-0.10	-0.07	0.01	0.11	0.03	0.10	-0.03	0.11	-0.06	0.19	-0.04
OTU0211	-10.13	-0.35	0.34	-0.30	0.11	-0.07	0.69	0.35	0.60	-0.45	-0.07	0.19	0.14	-0.27	-0.14	0.08	-0.04	0.11	-0.21	-0.07	-0.08	-0.10	-0.04	-0.23	0.05	-0.10	-0.03	0.04	0.12	0.11	0.01	0.09	0.07	-0.02	0.13	0.15
OTU0214	-9.33	-0.24	0.16	-0.26	0.25	-0.14	0.66	0.21	0.23	-0.35	-0.01	0.24	0.06	-0.17	-0.26	-0.05	-0.18	0.06	-0.27	-0.08	-0.02	-0.15	-0.05	-0.29	-0.08	-0.13	-0.06	-0.04	0.14	0.09	0.02	0.10	0.09	0.02	0.20	0.14
OTU0097	-9.65	-0.30	-0.01	-0.35	0.15	-0.05	0.72	0.45	0.67	-0.35	0.05	0.03	-0.03	-0.24	-0.24	-0.20	-0.23	0.00	-0.28	-0.04	-0.13	-0.18	-0.14	-0.35	-0.19	-0.18	-0.05	-0.04	0.09	0.13	0.07	0.03	0.10	0.06	0.30	0.09
OTU0037	-8.68	-0.56	-0.10	-0.47	0.13	-0.24	0.72	0.55	0.62	-0.36	0.04	0.06	-0.02	-0.22	-0.29	-0.13	-0.29	0.09	-0.32	-0.02	-0.17	-0.28	-0.03	-0.39	-0.22	-0.16	-0.05	0.05	0.12	0.03	0.03	0.02	0.12	0.14	0.30	0.10
OTU0181	-8.53	1.69	0.77	1.43	0.31	-0.19	0.46	-0.34	-0.59	-0.16	0.09	-0.05	0.12	-0.13	-0.23	-0.04	-0.14	-0.10	-0.16	0.02	-0.06	-0.08	-0.18	-0.27	0.07	-0.31	0.00	0.06	0.00	0.02	0.02	0.08	0.01	-0.23	0.26	-0.04
OTU0061	-6.56	2.12	1.01	1.67	0.38	-0.28	0.34	-0.70	-1.02	-0.15	0.21	0.00	0.02	-0.09	-0.23	-0.03	-0.18	0.00	-0.13	0.06	-0.03	-0.19	-0.16	-0.26	0.00	-0.31	0.01	0.03	-0.06	0.06	0.08	-0.08	0.01	-0.17	0.31	-0.09
OTU0012	-7.49	-0.23	0.28	0.02	0.26	-0.14	0.62	0.45	0.54	-0.29	0.04	-0.04	0.01	-0.18	-0.17	-0.16	-0.30	0.00	-0.17	-0.11	-0.09	-0.27	-0.01	-0.29	-0.07	-0.33	-0.04	0.02	0.08	0.04	0.15	-0.03	0.02	-0.05	0.25	-0.15
OTU0204	-7.30	-0.16	0.49	0.19	0.34	0.11	0.44	0.26	0.49	-0.34	0.03	0.05	0.04	-0.21	-0.22	-0.12	-0.26	0.04	-0.15	-0.01	-0.09	-0.13	-0.07	-0.23	-0.14	-0.20	-0.01	0.07	0.18	0.01	0.04	0.07	0.09	0.06	0.14	0.02
OTU0194	-7.88	-0.88	-0.04	-0.39	0.26	0.24	0.52	0.72	0.95	-0.42	-0.05	0.29	0.04	-0.17	-0.14	-0.06	-0.28	0.07	-0.08	-0.04	0.12	-0.09	-0.09	-0.28	-0.16	-0.36	-0.03	0.03	0.21	0.03	0.03	0.10	0.11	0.10	0.16	0.10
OTU0185	-5.43	-0.62	-0.23	-0.79	0.21	-0.22	0.39	0.59	0.85	-0.34	-0.05	0.02	-0.18	-0.18	-0.18	-0.12	-0.26	-0.01	-0.09	-0.08	-0.15	-0.20	-0.14	-0.37	-0.15	-0.28	-0.06	0.03	0.04	0.14	0.11	0.07	0.24	0.08	0.22	-0.10
OTU0177	-7.76	0.14	0.65	0.45	0.24	0.09	0.47	0.22	0.30	-0.36	0.12	0.15	-0.06	-0.16	-0.11	-0.10	-0.14	-0.01	-0.06	0.06	-0.02	-0.14	-0.05	-0.30	-0.12	-0.15	-0.11	0.04	0.03	0.06	-0.04	0.14	0.10	-0.06	0.31	0.01
OTU0196	-5.97	-0.39	-0.33	-0.51	-0.05	-0.17	0.37	0.96	1.07	-0.32	-0.06	0.15	-0.04	-0.12	-0.15	-0.11	-0.20	0.03	-0.09	-0.09	-0.08	-0.02	-0.19	-0.36	-0.04	-0.29	0.03	0.09	0.17	0.12	0.20	0.20	0.20	0.07	0.17	0.05
OTU0120	-7.80	-0.04	0.50	0.25	0.09	-0.12	0.53	0.39	0.55	-0.48	0.14	0.00	-0.06	-0.20	-0.18	-0.05	-0.21	0.10	-0.14	0.00	-0.24	-0.06	-0.12	-0.38	-0.09	-0.24	-0.07	-0.01	-0.03	0.09	0.09	0.06	0.21	-0.12	0.24	0.08
OTU0057	-7.36	-0.72	0.05	-0.18	0.41	-0.14	0.56	0.96	1.10	-0.42	-0.01	-0.09	-0.05	-0.21	-0.20	-0.20	-0.23	0.05	-0.14	-0.07	-0.24	-0.13	-0.20	-0.35	-0.18	-0.27	-0.13	0.01	0.10	0.02	0.05	0.02	0.20	0.05	0.27	-0.02
OTU0049	-7.34	0.31	0.37	0.55	0.21	-0.01	0.48	0.26	0.42	-0.32	0.13	0.21	-0.11	-0.09	-0.04	-0.28	-0.45	-0.06	-0.14	0.17	-0.15	-0.18	-0.18	-0.29	-0.30	-0.25	-0.05	0.05	0.17	0.00	0.10	0.09	0.15	0.05	0.31	-0.07
OTU0103	-10.32	1.40	1.03	1.07	0.10	-0.20	0.68	-0.78	-0.79	-0.28	0.17	0.09	-0.19	-0.07	-0.12	-0.17	-0.16	0.01	-0.16	0.07	-0.09	-0.29	-0.14	-0.29	-0.07	-0.37	-0.03	0.03	-0.21	0.04	0.20	-0.09	-0.01	0.00	0.30	-0.05
OTU0011	-9.59	0.58	0.61	0.76	0.06	0.00	0.72	0.17	0.09	-0.30	0.19	0.15	-0.22	-0.09	-0.12	-0.27	-0.39	-0.10	-0.16	0.12	-0.11	-0.31	-0.16	-0.35	-0.38	-0.40	-0.11	0.10	0.09	-0.10	0.16	-0.02	0.02	0.14	0.36	-0.03
OTU0186	-4.69	0.04	-0.23	-0.17	0.01	-0.19	0.30	0.28	0.23	-0.22	-0.20	0.14	-0.14	-0.14	-0.11	0.03	-0.12	0.01	-0.28	-0.06	-0.03	-0.20	-0.01	-0.25	-0.12	-0.22	0.10	0.11	0.32	0.03	0.16	0.10	0.12	-0.01	0.13	-0.08
OTU0152	-8.16	1.99	0.86	1.26	0.05	-0.32	0.44	-0.92	-1.25	-0.28	0.04	0.15	0.05	0.19	-0.08	0.20	0.09	0.10	-0.16	-0.05	0.25	-0.19	0.01	-0.07	0.46	-0.30	-0.02	0.03	-0.14	0.08	0.13	0.02	0.14	-0.14	0.21	-0.28
OTU0188	-6.84	1.89	0.82	0.57	-0.79	-0.49	0.38	-1.51	-1.43	-0.38	-0.05	0.11	-0.18	0.01	-0.10	0.35	0.22	0.15	-0.18	-0.04	0.02	-0.17	0.00	-0.20	0.13	-0.02	0.17	0.02	-0.03	0.09	0.12	-0.17	0.02	-0.06	0.10	-0.12
OTU0180	-4.81	-0.27	0.02	-0.23	0.30	-0.21	0.31	0.19	0.50	-0.35	-0.08	0.12	-0.10	-0.04	-0.14	0.02	-0.19	-0.01	-0.07	-0.01	-0.09	-0.13	-0.01	-0.33	-0.13	-0.24	0.09	0.10	0.08	-0.01	0.19	-0.03	0.12	-0.17	0.19	-0.04
OTU0160	-5.12	1.00	0.24	0.13	0.03	-0.38	0.32	-1.28	-1.32	-0.31	-0.01	0.10	-0.20	0.15	-0.22	0.03	0.03	0.03	-0.24	-0.09	-0.06	-0.32	0.21	-0.24	-0.18	-0.10	0.11	-0.05	0.01	-0.02	0.16	-0.08	0.05	-0.15	0.01	-0.19
OTU0142	-8.47	1.25	0.88	1.11	-0.11	0.17	0.43	-0.57	-0.41	-0.38	-0.06	0.00	-0.02	0.01	-0.08	0.25	0.44	-0.10	-0.22	0.07	-0.12	0.02	-0.08	-0.28	0.30	0.33	0.00	-0.01	0.08	-0.02	0.07	0.03	0.10	-0.14	0.38	-0.09
OTU0059	-5.63	0.38	0.88	0.37	0.24	-0.38	0.41	-0.93	-0.83	-0.26	-0.03	0.23	-0.16	0.09	-0.05	0.02	-0.08	0.13	-0.16	-0.05	0.05	-0.35	0.21	-0.28	-0.08	-0.18	0.15	-0.15	-0.03	0.03	0.29	-0.05	0.10	-0.12	0.12	-0.19
OTU0140	-8.91	1.48	1.42	1.32	0.04	-0.12	0.53	-1.18	-1.17	-0.24	0.02	0.12	-0.06	0.09	0.05	0.07	-0.03	0.14	-0.16	0.06	-0.05	-0.21	0.08	-0.17	-0.02	-0.15	0.07	-0.12	-0.25	0.13	0.22	-0.10	0.14	-0.29	0.11	0.00
OTU0098	-7.85	2.12	1.74	1.84	0.59	-0.31	0.43	-2.06	-2.26	-0.23	0.09	0.32	-0.10	0.30	-0.01	0.26	0.03	0.20	-0.19	0.03	0.23	-0.35	0.31	0.00	0.13	-0.10	0.10	-0.02	-0.27	0.20	0.13	-0.20	0.06	-0.44	0.13	-0.25
OTU0056	-4.22	-2.34	-1.54	-3.48	0.51	-0.16	0.46	0.90	1.51	-0.41	-0.44	-0.17	-0.35	-0.33	-0.14	-0.14	-0.05	0.09	-0.23	-0.51	-0.23	-0.47	0.01	-0.42	-0.19	-0.18	-0.05	-0.01	0.14	0.20	0.33	0.10	0.21	-0.13	0.09	-0.13
OTU0006	-7.94	1.44	1.54	1.58	0.00	-0.37	0.59	-1.30	-1.44	-0.22	0.20	0.36	-0.29	0.16	0.05	-0.01	-0.23	0.19	-0.16	0.10	0.15	-0.42	0.18	-0.15	-0.09	-0.20	0.05	-0.06	-0.19	-0.01	0.16	-0.26	0.05	0.07	0.21	-0.25
OTU0198	-6.24	1.95	1.33	1.69	0.32	-0.14	0.24	-1.34	-1.67	-0.21	0.08	0.24	-0.01	0.03	-0.04	0.15	-0.11	0.03	-0.22	0.15	0.14	-0.08	0.03	-0.15	-0.03	-0.23	0.00	0.03	-0.02	0.10	0.05	-0.15	0.01	-0.23	0.21	-0.05
OTU0205	-6.81	1.55	0.98	1.26	-0.12	-0.24	0.34	-0.97	-1.25	-0.15	-0.01	0.04	-0.14	-0.16	0.02	0.03	-0.06	0.07	-0.19	-0.02	-0.05	-0.14	-0.10	-0.20	-0.11	-0.23	0.09	0.10	-0.12	0.08	0.20	-0.13	0.05	-0.16	0.06	0.00
OTU0072	-6.11	1.68	0.47	0.91	0.11	-0.19	0.34	-0.71	-1.04	-0.07	0.02	0.14	-0.32	-0.14	-0.10	-0.07	-0.09	0.03	-0.13	-0.02	0.04	-0.24	-0.17	-0.29	-0.28	-0.29	0.03	0.19	-0.05	-0.06	0.23	-0.09	0.00	0.00	0.15	-0.07
OTU0048	-7.29	1.81	1.08	1.46	0.32	-0.30	0.45	-1.08	-1.53	-0.16	0.10	0.26	-0.23	0.06	-0.03	0.08	-0.04	0.06	-0.17	0.01	0.20	-0.36	0.04	-0.19	-0.10	-0.24	-0.01	0.05	-0.19	0.04	0.17	-0.20	0.09	-0.13	0.20	-0.15
OTU0004	-7.38	2.30	0.71	1.68	-0.09	-0.08	0.47	-0.70	-1.08	-0.20	0.24	0.26	-0.30	-0.02	-0.07	0.02	-0.18	0.08	-0.13	0.09	0.12	-0.33	-0.15	-0.25	-0.11	-0.34	-0.01	0.15	-0.05	-0.08	0.15	-0.21	0.01	0.09	0.33	-0.14
OTU0096	-5.54	0.17	-0.12	0.21	-0.15	-0.08	0.36	0.46	0.31	-0.29	-0.01	0.18	-0.14	-0.20	-0.21	-0.14	-0.15	-0.02	-0.20	0.09	-0.14	-0.10	-0.12	-0.37	-0.10	-0.23	-0.10	0.21	0.17	0.08	0.06	0.08	0.07	0.07	0.24	-0.16
OTU0161	-7.96	0.08	0.81	0.97	0.58	0.04	0.48	0.25	-0.04	-0.14	0.12	0.26	0.00	-0.12	-0.12	-0.01	-0.20	-0.09	-0.29	0.12	-0.01	-0.03	0.04	-0.24	-0.03	-0.19	-0.07	0.18	0.15	-0.01	-0.03	0.09	0.22	-0.08	0.23	-0.10
OTU0217	-6.82	1.25	0.69	0.92	0.69	-0.46	0.39	-0.65	-1.00	-0.13	0.10	0.21	-0.11	-0.02	-0.20	0.02	-0.05	-0.01	-0.26	0.00	-0.05	-0.07	0.12	-0.27	-0.05	-0.23	-0.03	0.10	0.08	0.15	0.03	0.06	0.18	-0.18	0.24	-0.15
OTU0164	-8.95	1.54	0.65	0.75	-0.35	-0.36	0.56	-0.78	-1.01	-0.22	0.08	0.30	-0.12	-0.03	-0.20	0.21	0.03	0.19	-0.17	-0.03	0.04	-0.03	0.05	-0.24	0.04	-0.22	0.13	0.11	0.03	0.16	0.12	-0.06	0.05	-0.08	0.20	-0.07
OTU0107	-9.56	0.14	0.84	0.66	0.15	-0.11	0.66	-0.08	-0.04	-0.27	0.07	0.24	0.00	-0.10	-0.14	0.06	-0.03	0.03	-0.21	0.01	-0.10	-0.01	0.19	-0.30	-0.02	-0.15	0.01	0.05	0.13	0.10	0.01	0.09	0.15	-0.13	0.22	-0.03
OTU0091	-7.82	2.54	1.00	1.83	-0.14	-0.08	0.40	-1.29	-1.79	-0.17	0.21	0.52	-0.16	0.12	-0.11	0.12	-0.09	0.16	-0.21	0.14	0.34	-0.03	-0.03	-0.10	0.04	-0.41	0.18	0.12	-0.11	0.00	0.12	-0.18	0.08	0.00	0.17	-0.10
OTU0010	-8.74	0.14	0.89	0.96	0.06	-0.24	0.68	0.16	0.18	-0.24	0.25	0.35	-0.06	-0.06	-0.08	-0.05	-0.19	0.06	-0.28	0.10	0.15	-0.11	0.13	-0.31	-0.12	-0.30	-0.03	0.02	0.11	0.02	0.10	0.00	0.17	0.06	0.29	-0.10
OTU0209	-5.61	-0.55	-0.37	-0.46	0.16	-0.01	0.39	0.84	0.86	-0.23	0.04	0.16	-0.17	-0.21	-0.20	-0.14	-0.22	-0.09	-0.24	-0.01	-0.11	-0.06	-0.06	-0.37	-0.12	-0.34	0.02	0.22	0.20	0.05	0.10	0.10	0.10	0.04	0.26	-0.09
OTU0166	-8.69	0.61	1.29	1.48	0.18	-0.12	0.52	-0.38	-0.50	-0.13	0.15	0.21	-0.01	-0.12	0.04	-0.07	-0.18	-0.10	-0.25	0.20	-0.09	0.01	0.00	-0.25	-0.03	-0.18	0.09	0.13	-0.03	-0.01	0.05	0.01	0.09	-0.07	0.15	-0.06
OTU0158	-7.20	-0.07	0.29	0.36	0.27	-0.02	0.47	0.38	0.39	-0.22	0.10	0.11	-0.13	-0.15	-0.11	-0.14	-0.13	-0.15	-0.27	0.03	-0.12	-0.11	-0.03	-0.28	-0.10	-0.25	-0.03	0.19	0.12	-0.02	0.00	0.17	0.15	-0.08	0.28	-0.08
OTU0148	-7.93	1.34	0.77	1.38	0.30	-0.02	0.41	-0.25	-0.64	-0.12	0.18	0.30	-0.06	-0.05	-0.09	-0.07	-0.10	-0.13	-0.21	0.15	0.01	0.00	-0.05	-0.26	0.04	-0.28	0.02	0.18	0.08	0.02	0.01	0.04	0.12	-0.12	0.25	-0.09
OTU0115	-9.57	0.82	0.88	1.49	0.07	0.25	0.55	-0.06	-0.19	-0.14	0.19	0.29	0.02	-0.07	-0.09	-0.02	-0.18	-0.14	-0.22	0.21	-0.01	0.11	0.00	-0.22	0.02	-0.27	-0.01	0.16	0.19	-0.02	0.02	0.10	0.12	-0.01	0.21	-0.04
OTU0077	-7.68	-0.32	0.13	0.21	0.04	0.05	0.53	0.59	0.73	-0.26	0.04	0.21	-0.04	-0.14	-0.12	-0.08	-0.22	-0.07	-0.25	0.07	0.02	0.02	0.02	-0.26	-0.04	-0.29	0.02	0.22	0.20	0.03	0.12	0.14	0.16	-0.02	0.21	-0.10
OTU0071	-8.53	0.31	0.66	0.72	0.06	-0.13	0.60	-0.12	-0.18	-0.24	0.16	0.19	-0.11	-0.14	-0.09	-0.11	-0.09	-0.05	-0.28	0.08	-0.06	-0.13	0.02	-0.23	-0.03	-0.20	0.00	0.08	0.05	0.03	0.04	0.01	0.07	0.00	0.27	-0.10
OTU0064	-8.40	0.42	0.18	0.38	-0.03	0.02	0.56	0.34	0.39	-0.26	0.12	0.27	-0.12	-0.07	-0.11	-0.05	-0.11	-0.02	-0.19	0.06	0.06	-0.03	-0.03	-0.25	0.04	-0.28	0.04	0.22	0.13	0.03	0.09	0.04	0.15	0.05	0.37	-0.05
OTU0043	-9.55	0.87	0.53	1.10	-0.03	-0.09	0.64	0.09	-0.10	-0.20	0.19	0.26	-0.08	-0.05	-0.13	-0.06	-0.16	-0.06	-0.30	0.17	0.03	0.00	0.02	-0.26	0.00	-0.27	0.00	0.13	0.11	0.07	0.02	0.03	0.17	0.01	0.37	-0.11
OTU0021	-9.95	1.28	0.51	1.19	0.05	0.09	0.66	0.18	-0.08	-0.17	0.24	0.30	-0.16	-0.04	-0.10	-0.14	-0.20	-0.06	-0.14	0.13	0.01	-0.10	-0.08	-0.32	-0.09	-0.43	0.03	0.23	0.08	0.01	0.09	0.02	0.06	0.02	0.45	-0.09
OTU0013	-7.61	-0.25	0.13	0.17	0.21	-0.16	0.61	0.50	0.56	-0.23	0.14	0.15	-0.20	-0.14	-0.19	-0.19	-0.24	0.00	-0.22	0.04	-0.07	-0.16	0.03	-0.31	-0.19	-0.31	-0.02	0.10	0.13	0.07	0.08	0.10	0.12	0.05	0.34	-0.13
OTU0008	-8.95	-0.19	0.41	0.38	0.09	-0.08	0.73	0.58	0.58	-0.29	0.25	0.27	-0.14	-0.10	-0.05	-0.13	-0.23	-0.04	-0.26	0.09	-0.03	-0.18	0.03	-0.32	-0.20	-0.31	-0.03	0.04	0.16	-0.02	0.13	0.08	0.15	0.07	0.35	-0.11
OTU0005	-9.02	-0.62	0.13	0.05	0.29	-0.04	0.77	0.77	0.92	-0.30	0.16	0.22	-0.16	-0.11	-0.14	-0.22	-0.26	-0.02	-0.26	0.05	-0.08	-0.20	0.05	-0.37	-0.21	-0.33	-0.03	0.05	0.20	0.00	0.12	0.08	0.11	0.12	0.39	-0.09
OTU0218	-8.64	0.58	0.55	0.62	-0.11	-0.12	0.56	-0.34	-0.43	-0.24	0.13	0.10	-0.15	-0.14	-0.22	0.10	-0.07	0.07	-0.18	0.05	-0.02	-0.08	-0.04	-0.30	-0.13	-0.22	0.07	0.19	0.02	0.12	0.08	-0.04	0.04	-0.05	0.20	0.08
OTU0003	-11.61	2.63	1.87	2.68	0.04	-0.30	0.81	-1.57	-1.91	-0.14	0.46	0.27	-0.26	0.03	-0.08	0.02	-0.17	0.13	-0.17	0.24	0.02	-0.25	0.03	-0.13	-0.18	-0.34	0.12	0.08	-0.18	0.02	0.15	-0.22	-0.07	-0.01	0.33	-0.09
OTU0100	-5.76	0.50	1.47	1.21	0.19	0.12	0.28	-0.50	-0.52	-0.18	0.10	0.49	-0.01	0.00	0.10	0.03	-0.09	-0.04	-0.22	0.12	0.03	-0.03	0.17	-0.15	0.00	-0.02	-0.08	-0.05	0.14	-0.01	0.09	0.05	0.03	-0.09	-0.01	0.06
OTU0207	-6.43	1.65	1.44	1.75	0.23	-0.02	0.27	-1.20	-1.49	-0.22	0.19	0.29	0.13	0.19	-0.01	-0.03	0.00	-0.03	-0.23	0.11	0.05	-0.16	0.11	-0.11	0.01	-0.11	-0.03	0.03	0.01	-0.02	0.06	-0.02	0.01	-0.15	0.13	0.02
OTU0157	-7.03	1.75	1.83	1.88	0.17	-0.13	0.33	-1.43	-1.67	-0.19	0.22	0.27	-0.03	0.04	-0.01	-0.03	0.06	0.07	-0.11	0.29	-0.05	-0.15	0.13	-0.18	-0.04	-0.08	-0.04	-0.04	-0.10	0.11	0.06	-0.09	-0.14	-0.12	0.17	-0.06
OTU0144	-8.36	0.93	1.10	1.30	0.67	0.16	0.47	-0.59	-0.60	-0.28	0.04	0.08	0.04	0.02	-0.07	-0.15	-0.07	0.00	-0.13	0.15	-0.09	-0.21	0.05	-0.18	-0.08	-0.12	-0.17	0.11	0.00	-0.07	0.04	-0.02	-0.05	-0.05	0.19	-0.03
OTU0118	-5.48	0.98	0.54	0.81	0.06	-0.05	0.29	-0.45	-0.36	-0.32	0.12	0.04	-0.09	0.03	-0.14	-0.09	-0.06	0.07	-0.05	0.05	0.05	-0.29	-0.06	-0.13	-0.03	-0.23	-0.13	0.00	-0.01	0.06	0.15	-0.08	-0.04	0.02	0.24	-0.11
OTU0090	-6.99	-0.35	0.22	0.01	0.15	0.01	0.50	0.50	0.74	-0.43	-0.04	0.22	0.06	0.02	-0.07	-0.19	-0.11	0.02	-0.17	0.05	-0.06	-0.16	0.00	-0.33	-0.08	-0.24	-0.12	0.08	0.25	-0.07	0.12	0.09	-0.03	0.07	0.25	0.03
OTU0055	-8.23	1.71	1.84	2.26	0.55	-0.11	0.47	-1.29	-1.54	-0.14	0.17	0.29	0.13	0.19	0.02	-0.15	-0.17	-0.06	-0.19	0.19	-0.02	-0.25	0.14	-0.13	-0.14	-0.20	-0.15	0.01	-0.01	-0.08	0.16	-0.08	-0.07	-0.10	0.28	-0.11
OTU0222	-5.72	0.77	1.22	0.84	0.51	-0.31	0.31	-1.42	-1.60	-0.28	0.04	0.18	-0.07	0.06	0.00	0.01	-0.04	0.09	-0.18	-0.01	0.08	-0.37	0.15	0.00	-0.01	-0.03	-0.13	0.00	-0.11	0.03	0.09	-0.18	-0.05	-0.20	0.04	-0.08
OTU0101	-7.40	2.17	1.29	1.93	-0.35	0.16	0.34	-1.21	-1.62	-0.24	0.18	0.40	-0.04	0.14	0.01	0.13	-0.08	0.10	-0.08	0.22	0.26	-0.20	-0.02	0.04	0.14	-0.24	-0.07	0.15	0.01	-0.09	0.09	-0.14	-0.11	0.07	0.20	-0.04
OTU0069	-7.83	1.63	0.91	1.67	0.05	0.22	0.39	-0.70	-0.94	-0.14	0.09	0.40	-0.01	0.05	-0.01	0.02	-0.12	0.10	0.04	0.19	0.34	-0.11	-0.03	0.03	0.07	-0.29	-0.05	0.16	0.04	-0.10	0.09	-0.06	-0.04	0.03	0.27	-0.04
OTU0086	-5.05	0.62	0.44	0.40	0.42	-0.17	0.31	-1.00	-1.22	-0.27	-0.05	0.13	-0.16	0.08	-0.14	-0.09	-0.14	0.11	-0.12	-0.01	0.14	-0.44	0.11	-0.04	-0.01	-0.24	-0.15	0.10	-0.18	0.04	0.07	-0.15	0.06	-0.03	-0.02	-0.22
OTU0026	-4.59	-0.06	-0.42	-0.60	0.17	0.08	0.35	0.15	0.42	-0.42	-0.09	-0.07	-0.23	-0.06	-0.11	-0.14	-0.13	0.02	-0.12	-0.10	-0.03	-0.36	-0.01	-0.15	-0.01	-0.31	-0.13	0.12	0.01	0.01	0.12	-0.11	0.14	0.09	0.24	-0.14
OTU0146	-5.69	0.87	0.42	0.41	-0.12	-0.14	0.32	-0.50	-0.56	-0.35	0.02	0.23	-0.12	0.04	-0.13	-0.05	0.02	0.14	-0.09	0.01	0.10	-0.24	0.03	-0.27	-0.09	-0.12	-0.08	0.18	0.10	-0.01	0.11	-0.10	-0.22	0.19	0.13	-0.01
OTU0087	-5.42	2.32	0.96	1.47	0.04	-0.21	0.22	-1.31	-1.46	-0.25	0.16	0.22	-0.18	0.13	-0.14	0.03	0.07	0.13	-0.11	0.05	0.05	-0.20	-0.02	-0.19	-0.05	-0.04	0.00	0.18	-0.03	-0.01	0.15	-0.17	-0.15	0.04	0.13	0.05
OTU0052	-4.24	0.49	0.11	-0.07	-0.02	-0.19	0.30	-0.27	-0.18	-0.34	0.05	0.23	-0.27	0.03	-0.15	-0.23	-0.08	0.11	-0.12	-0.07	-0.04	-0.35	0.03	-0.34	-0.23	-0.08	-0.10	0.15	0.17	-0.03	0.25	-0.13	-0.21	0.25	0.15	-0.11
OTU0025	-5.35	0.34	0.36	0.23	0.15	-0.11	0.37	-0.03	0.21	-0.34	0.07	0.22	-0.18	0.05	-0.10	-0.24	-0.04	0.04	-0.12	0.04	0.01	-0.31	0.00	-0.32	-0.20	-0.12	-0.10	0.06	0.06	-0.06	0.27	-0.05	-0.17	0.30	0.13	0.02
OTU0024	-7.36	1.87	1.71	1.94	0.52	-0.13	0.44	-1.61	-1.80	-0.27	0.34	0.52	0.02	0.31	-0.05	0.13	-0.13	0.36	-0.13	0.18	0.33	-0.19	0.27	-0.10	0.02	-0.14	0.11	-0.07	-0.01	0.03	0.05	-0.15	-0.03	-0.16	0.20	-0.10
OTU0149	-8.51	2.60	1.79	2.66	0.08	0.03	0.36	-1.33	-1.83	-0.17	0.17	0.22	0.09	0.22	0.03	0.03	-0.04	-0.03	-0.13	0.21	0.03	-0.18	-0.04	-0.02	0.09	-0.20	-0.07	0.08	-0.26	-0.02	0.00	-0.13	-0.02	-0.10	0.29	-0.14

OTU	(Intercept)	Log type: natural	Year: 2020	Year: 2021	Mortality factor: uprooted	Decay stage	Sequencing depth	Log type: natural x Year: 2020	Log type: natural x Year: 2021	Year: 2020 x *Antrodia piceata*	Year: 2020 x *Antrodiella citrinella*
OTU0067	-8.82	2.67	1.64	2.58	-0.33	-0.03	0.43	-1.40	-1.62	-0.18	0.22
OTU0208	-9.57	1.06	1.00	1.48	-0.37	0.45	0.47	-0.25	-0.18	-0.28	0.02
OTU0174	-7.52	2.25	1.18	1.80	-0.01	-0.13	0.36	-1.00	-1.21	-0.26	0.15
OTU0113	-9.77	0.16	0.64	0.74	0.13	0.22	0.64	0.15	0.33	-0.34	0.02
OTU0028	-7.45	2.65	1.41	1.98	-0.26	-0.26	0.42	-1.41	-1.57	-0.22	0.24
OTU0054	-7.63	1.70	0.96	1.38	-0.02	-0.08	0.43	-0.63	-0.73	-0.32	0.14
OTU0017	-7.11	0.34	0.78	0.87	0.07	-0.21	0.53	-0.33	-0.23	-0.29	0.10
OTU0082	-6.80	1.52	1.40	1.47	-0.11	-0.22	0.36	-0.64	-0.75	-0.19	0.20
OTU0020	-7.90	0.84	1.14	1.42	0.08	-0.07	0.52	-0.14	-0.08	-0.23	0.18
OTU0016	-5.81	1.72	1.74	2.28	0.18	-0.21	0.31	-0.92	-1.06	-0.08	0.24
OTU0007	-7.86	1.39	1.45	1.58	-0.20	-0.27	0.57	-1.24	-1.19	-0.36	0.27
OTU0189	-6.38	1.69	1.14	1.36	-0.28	-0.20	0.30	-0.87	-1.18	-0.25	0.15
OTU0175	-6.48	1.40	1.92	2.20	0.39	-0.14	0.29	-1.17	-1.36	-0.18	0.25
OTU0058	-6.25	0.60	1.36	1.22	0.28	-0.12	0.38	-0.45	-0.41	-0.31	0.15
OTU0032	-5.27	0.61	-0.11	-0.40	-0.20	-0.43	0.44	-0.45	-0.40	-0.33	-0.02
OTU0002	-6.68	1.27	1.23	1.30	0.22	-0.34	0.57	-0.89	-0.95	-0.27	0.31
OTU0001	-6.65	1.78	1.97	1.96	-0.09	-0.67	0.57	-1.98	-2.02	-0.12	0.36

OTU	Year: 2020 x *Fomitopsis rosea*	Year: 2020 x *Perenniporia subacida*	Year: 2020 x *Physisporinus crocatus*	Year: 2020 x *Postia guttulata*	Year: 2020 x *Skeletocutis odora*	Year: 2020 x *Skeletocutis stellae*	Year: 2020 x *Steccherinum collabens*	Year: 2021 x *Antrodia piceata*	Year: 2021 x *Antrodiella citrinella*	Year: 2021 x *Fomitopsis rosea*	Year: 2021 x *Perenniporia subacida*	Year: 2021 x *Physisporinus crocatus*	Year: 2021 x *Postia guttulata*
OTU0067	0.15	0.00	0.12	0.04	-0.01	-0.04	0.00	-0.16	0.29	-0.03	-0.12	-0.02	-0.13
OTU0208	0.29	0.19	0.07	0.02	0.07	-0.01	0.00	-0.05	0.24	0.12	0.09	0.01	-0.13
OTU0174	0.24	0.08	0.22	-0.02	-0.01	0.02	0.02	-0.13	0.16	0.03	-0.11	0.09	-0.18
OTU0113	0.26	0.16	0.08	-0.02	-0.09	-0.11	-0.02	-0.07	0.14	0.11	-0.07	0.11	-0.25
OTU0028	0.41	-0.07	0.21	0.06	-0.04	-0.09	0.22	-0.07	0.17	0.15	-0.20	0.10	-0.12
OTU0054	0.27	0.01	0.11	-0.02	-0.04	0.02	0.08	-0.13	0.15	0.02	-0.17	-0.02	-0.19
OTU0017	0.05	-0.16	0.02	-0.06	-0.27	-0.09	0.01	-0.24	0.16	-0.24	-0.33	0.11	-0.28
OTU0082	0.36	-0.01	-0.08	0.06	-0.08	-0.05	-0.01	-0.02	0.09	0.10	-0.28	-0.06	-0.20
OTU0020	0.31	0.01	0.01	0.01	-0.22	-0.16	-0.08	-0.09	0.16	0.09	-0.29	0.05	-0.28
OTU0016	0.30	-0.05	-0.01	0.10	-0.16	-0.15	-0.03	-0.05	0.23	0.09	-0.30	-0.04	-0.16
OTU0007	0.22	-0.21	0.06	0.07	-0.15	-0.11	0.08	-0.17	0.19	-0.04	-0.38	0.20	-0.23
OTU0189	0.10	-0.09	0.00	-0.05	-0.16	-0.07	0.02	-0.11	0.14	-0.20	-0.22	-0.16	-0.17
OTU0175	0.15	0.08	0.05	0.06	-0.10	-0.13	0.01	-0.11	0.28	-0.13	-0.24	0.07	-0.09
OTU0058	0.16	-0.07	0.04	-0.02	-0.13	-0.12	0.02	-0.12	0.15	-0.16	-0.29	0.05	-0.16
OTU0032	0.07	-0.41	-0.06	-0.11	-0.30	-0.16	0.20	-0.12	-0.10	-0.23	-0.46	-0.10	-0.30
OTU0002	0.18	-0.35	0.13	0.00	-0.32	-0.30	0.21	-0.11	0.13	-0.13	-0.58	0.06	-0.13
OTU0001	0.22	-0.39	0.21	-0.04	-0.30	-0.20	0.22	-0.10	0.23	0.02	-0.64	0.12	-0.13

OTU	Year: 2021 x *Skeletocutis odora*	Year: 2021 x *Skeletocutis stellae*	Year: 2021 x *Steccherinum collabens*	Log type: natural x *Antrodia piceata*	Log type: natural x *Antrodiella citrinella*	Log type: natural x. *Fomitopsis rosea*	Log type: natural x *Perenniporia subacida*	Log type: natural x *Physisporinus crocatus*	Log type: natural x *Postia guttulata*	Log type: natural x *Skeletocutis odora*	Log type: natural x *Skeletocutis stellae*	Log type: natural x *Steccherinum collabens*
OTU0067	-0.01	-0.14	0.04	0.01	-0.14	-0.02	0.08	-0.08	-0.11	-0.08	0.27	-0.07
OTU0208	0.25	-0.11	-0.12	0.15	0.21	-0.07	0.01	0.09	0.02	0.07	0.30	-0.02
OTU0174	0.15	-0.21	-0.12	0.02	-0.01	0.03	0.11	-0.04	0.05	-0.09	0.36	-0.14
OTU0113	0.03	-0.18	-0.17	0.05	0.25	-0.07	0.14	0.10	0.03	0.03	0.30	-0.04
OTU0028	0.03	-0.22	-0.02	0.09	-0.06	-0.03	0.17	-0.19	0.00	0.02	0.39	-0.11
OTU0054	0.03	-0.11	-0.01	0.14	0.00	-0.02	0.12	-0.10	0.04	0.05	0.42	0.03
OTU0017	-0.19	-0.05	-0.10	0.01	0.11	-0.07	0.14	-0.03	-0.05	0.10	0.28	-0.12
OTU0082	-0.04	-0.25	-0.04	0.08	0.03	-0.06	0.02	0.07	-0.16	-0.01	0.28	0.03
OTU0020	-0.15	-0.27	-0.10	-0.01	0.11	-0.07	0.01	0.10	-0.13	0.03	0.35	-0.03
OTU0016	-0.12	-0.24	0.03	0.00	-0.09	-0.15	0.19	-0.01	-0.19	0.14	0.24	-0.09
OTU0007	-0.23	-0.15	-0.02	-0.10	-0.10	-0.07	0.21	-0.21	-0.05	-0.08	0.29	-0.06
OTU0189	-0.17	-0.16	-0.06	0.08	-0.03	-0.05	0.11	-0.06	-0.12	-0.07	0.08	-0.07
OTU0175	-0.12	-0.12	-0.01	-0.01	-0.05	-0.05	0.06	-0.05	-0.10	-0.18	0.07	-0.08
OTU0058	-0.13	-0.07	-0.06	0.00	0.02	-0.06	0.09	0.02	-0.07	-0.07	0.13	-0.08
OTU0032	-0.37	-0.24	0.02	0.05	0.01	-0.01	0.24	-0.11	-0.07	0.08	0.18	-0.18
OTU0002	-0.37	-0.27	-0.05	-0.06	-0.16	-0.08	0.23	-0.24	-0.07	0.05	0.29	-0.25
OTU0001	-0.35	-0.18	0.04	-0.07	-0.26	-0.03	0.19	-0.44	-0.18	0.21	0.15	-0.28

Table S6. Posterior probabilities for regression parameters (Beta) in the community facets model describing the effects of model predictors on the response variables. Response variables included OTU richness, measured separately for all, common, and rare resident OTUs, DNA amount, and community composition measured by two latent variables, LV1 and LV2. Posterior probabilities are shown separately for positive (*+)* and negative (–) responses, and positive and negative responses with considerable statistical support are highlighted with red and blue, respectively (the darkest shade for posterior probability ≥ 0.95, medium shade for posterior probability ≥ 0.90, and the lightest shade for posterior probability ≥ 0.85). Felled log type, sampling year 2019, and the interaction term between the control treatment and sampling years 2020 and 2021 and between the control treatment and felled log type are included in the intercept as reference levels.

		**Response variable**					
	**Response**	***OTU richness***			***DNA***	***Community composition***	
**Predictor**	**type**	*All*	*Common*	*Rare*	***amount***	*LV1*	*LV2*
(Intercept)	+	1.000	0.000	0.000	0.000	0.000	0.000
	–	0.000	1.000	1.000	1.000	1.000	1.000
Log type: natural	+	0.901	0.955	0.996	1.000	1.000	1.000
	–	0.099	0.045	0.004	0.000	0.000	0.000
Year 2020	+	0.330	1.000	1.000	0.998	1.000	1.000
	–	0.670	0.000	0.000	0.002	0.000	0.000
Year 2021	+	0.886	0.993	0.987	1.000	1.000	1.000
	–	0.114	0.007	0.013	0.000	0.000	0.000
Mortality factor	+	0.005	0.688	0.160	0.853	0.328	0.469
	–	0.995	0.312	0.840	0.147	0.672	0.531
Decay stage	+	0.580	0.635	0.831	0.184	0.005	1.000
	–	0.420	0.365	0.169	0.816	0.995	0.000
Sequencing depth	+	0.746	1.000	1.000	1.000	1.000	0.063
	–	0.254	0.000	0.000	0.000	0.000	0.937
Log type: natural x Year 2020	+	0.534	0.884	0.286	0.000	0.002	0.000
	–	0.466	0.116	0.714	1.000	0.998	1.000
Log type: natural x Year 2021	+	0.473	0.999	0.976	0.053	0.001	0.000
	–	0.527	0.001	0.024	0.947	0.999	1.000
Antrodia piceata x Year 2020	+	0.663	0.037	0.086	0.029	0.079	0.769
	–	0.337	0.963	0.914	0.971	0.921	0.231
Antrodiella citrinella x Year 2020	+	0.914	0.819	0.869	0.811	0.865	0.793
	–	0.086	0.181	0.131	0.189	0.135	0.207
Fomitopsis rosea x Year 2020	+	0.874	0.930	0.841	0.764	0.939	0.890
	–	0.126	0.070	0.159	0.236	0.061	0.110
Perenniporia subacida x Year 2020	+	0.608	0.170	0.410	0.108	0.357	0.900
	–	0.392	0.830	0.590	0.892	0.643	0.100
Physisporinus crocatus x Year 2020	+	0.436	0.279	0.447	0.382	0.386	0.634
	–	0.564	0.721	0.553	0.618	0.614	0.366
Postia guttulata x Year 2020	+	0.952	0.656	0.601	0.578	0.553	0.858
	–	0.048	0.344	0.399	0.422	0.447	0.142
Skeletocutis odora x Year 2020	+	0.351	0.451	0.295	0.341	0.413	0.853
	–	0.649	0.549	0.705	0.659	0.587	0.147
Skeletocutis stellae x Year 2020	+	0.665	0.228	0.177	0.043	0.154	0.579
	–	0.335	0.772	0.823	0.957	0.846	0.421
Steccherinum collabens x Year 2020	+	0.701	0.690	0.681	0.295	0.655	0.313
	–	0.299	0.310	0.319	0.705	0.345	0.687
Antrodia piceata x Year 2021	+	0.501	0.192	0.043	0.105	0.144	0.902
	–	0.499	0.808	0.957	0.895	0.856	0.098
Antrodiella citrinella x Year 2021	+	0.333	0.805	0.891	0.761	0.821	0.852
	–	0.667	0.195	0.109	0.239	0.179	0.148
Fomitopsis rosea x Year 2021	+	0.131	0.723	0.376	0.874	0.578	0.993
	–	0.869	0.277	0.624	0.126	0.422	0.007
Perenniporia subacida x Year 2021	+	0.209	0.054	0.134	0.060	0.206	0.835
	–	0.791	0.946	0.866	0.940	0.794	0.165
Physisporinus crocatus x Year 2021	+	0.232	0.534	0.724	0.629	0.778	0.202
	–	0.768	0.466	0.276	0.371	0.222	0.798
Postia guttulata x Year 2021	+	0.430	0.308	0.074	0.357	0.108	0.972
	–	0.570	0.692	0.926	0.643	0.892	0.028
Skeletocutis odora x Year 2021	+	0.283	0.344	0.261	0.116	0.381	0.923
	–	0.717	0.656	0.739	0.884	0.619	0.077

		Response variable					
		OTU richness			*DNA*	*Community composition*	
**Predictor**	**Response type**	*All*	*Common*	*Rare*	*amount*	*LV1*	*LV2*
Skeletocutis stellae x	+	0.233	0.079	0.081	0.153	0.139	0.342
Year 2021	–	0.767	0.921	0.919	0.847	0.861	0.658
Steccherinum collabens x	+	0.500	0.732	0.679	0.448	0.662	0.771
Year 2021	–	0.500	0.268	0.321	0.552	0.338	0.229
Antrodia piceata x	+	0.454	0.712	0.526	0.580	0.547	0.703
Log type: natural	–	0.546	0.288	0.474	0.420	0.453	0.297
Antrodiella citrinella x	+	0.406	0.709	0.523	0.737	0.563	0.483
Log type: natural	–	0.594	0.291	0.477	0.263	0.437	0.517
Fomitopsis rosea x	+	0.675	0.286	0.509	0.089	0.220	0.063
Log type: natural	–	0.325	0.714	0.491	0.911	0.780	0.937
Perenniporia subacida x	+	0.957	0.746	0.659	0.667	0.602	0.268
Log type: natural	–	0.043	0.254	0.341	0.333	0.398	0.732
Physisporinus crocatus x	+	0.384	0.620	0.436	0.304	0.324	0.854
Log type: natural	–	0.616	0.380	0.564	0.696	0.676	0.146
Postia guttulata x	+	0.346	0.304	0.310	0.222	0.226	0.735
Log type: natural	–	0.654	0.696	0.690	0.778	0.774	0.265
Skeletocutis odora x	+	0.878	0.496	0.478	0.357	0.211	0.902
Log type: natural	–	0.122	0.504	0.522	0.643	0.789	0.098
Skeletocutis stellae x	+	0.889	0.977	0.990	0.872	0.974	0.926
Log type: natural	–	0.111	0.023	0.010	0.128	0.026	0.074
Steccherinum collabens x	+	0.177	0.316	0.227	0.201	0.252	0.845
Log type: natural	–	0.823	0.684	0.773	0.799	0.748	0.155

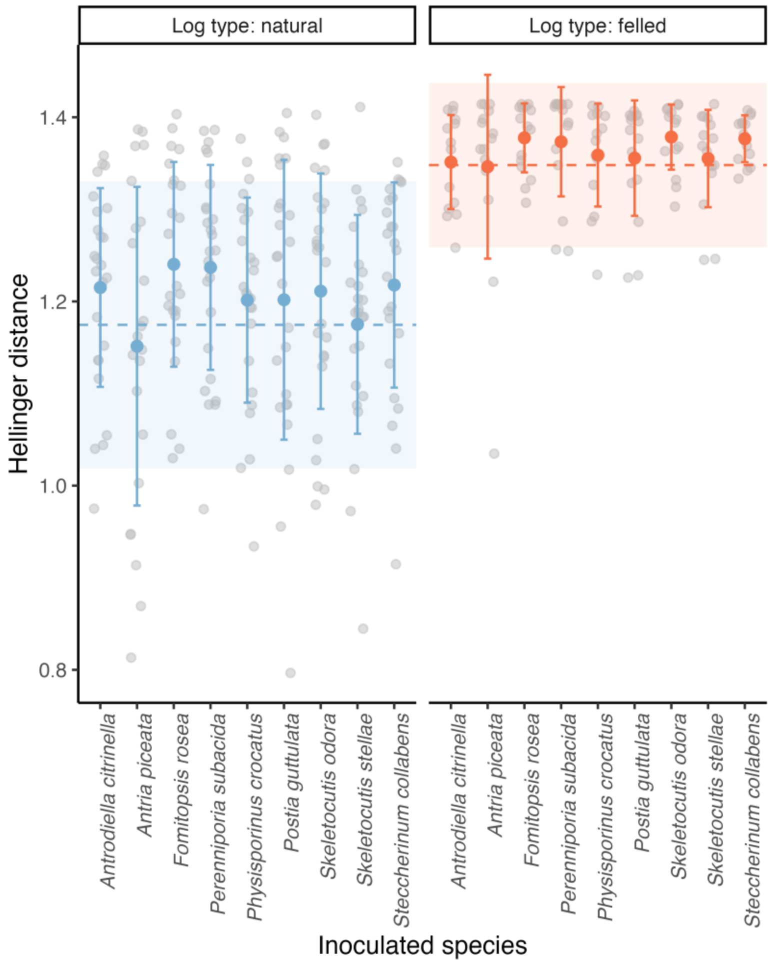

Figure S6. Change in the resident community composition in inoculated treatment logs and in control logs, separately for the natural and the felled log type. Community change was measured as Hellinger distance (Euclidean distance of Hellinger transformed read count data) between two time points: before the inoculations (2019) and two years after the inoculations (2021). Errors bars show the mean community dissimilarities and the standard deviations for treatment logs inoculated with different target species, and the grey points show the raw data for each inoculated species. The dotted lines and the shaded areas show the mean community dissimilarities and standard deviations, respectively, for natural and felled control logs in which no fungal species were inoculated in.

Table S7. Results from one-way ANOVA conducted to compare the resident community dissimilarity in logs with different inoculation treatments, separately for the natural and the felled log type. The table shows degrees of freedom (*Df*), sum of squares (*Sum Sq*), mean of the sum of squares (*Mean Sq*), F value (*F*), and p value (*p*). Treatment included ten levels: the nine inoculated target species (for each target species, $n_{natural}$ = 25, n_{felled} = 15) and the control ($n_{natural}$ = 50, n_{felled} = 50). Community dissimilarity was measured as Hellinger distance (Euclidean distance of Hellinger transformed read count data) between two time points: before the inoculations (2019) and two years after the inoculations (2021).

	Df	Sum Sq	Mean Sq	F	p
**Log type: natural**					
Treatment	9	0.190	0.021	1.207	0.291
Residuals	261	4.576	0.018		
**Log type: felled**					
Treatment	9	0.028	0.003	0.678	0.728
Residuals	173	0.795	0.005		

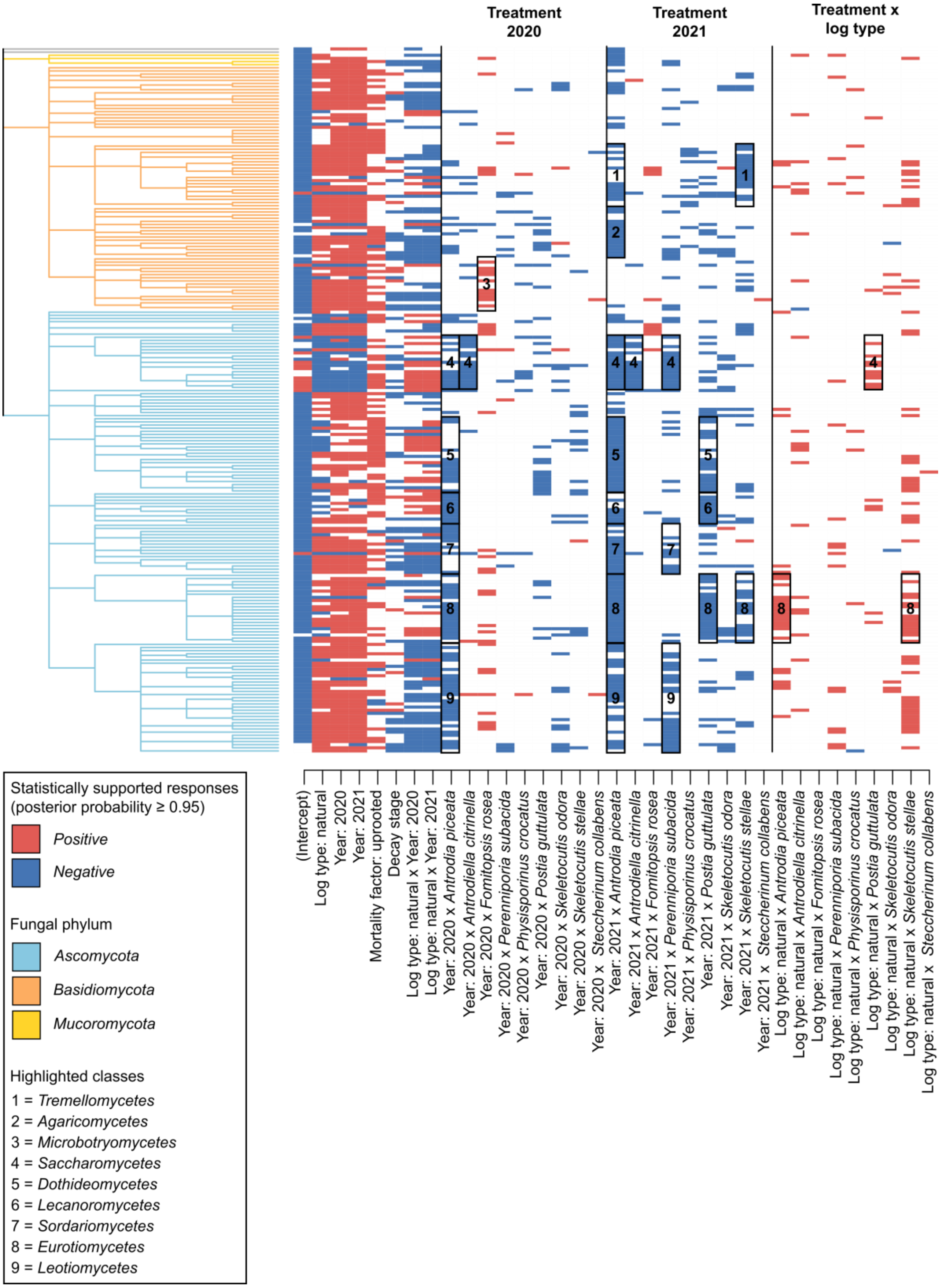

Figure S7. Posterior support for regression parameters (Beta) describing the responses of each OTU to the predictors in the alternative version of presence-absence model where sequencing depth was controlled through rarefaction rather than by including it as a covariate. Plot shows those positive (red) and negative (blue) responses to the model predictors that obtained at least 0.95 posterior probability. OTUs are displayed as rows, and they are ordered based on their taxonomic relationship. The rectangles highlight the fungal classes that show consistent responses, with the criteria that the class has at least 10 OTUs and at least 50% of them show a statistically supported responses of the same sign. The taxonomical tree is colored by the fungal phyla. Taxonomic assignment for each OTU is provided in Supporting Information: Table S5.

Section S5.1. Effect of inoculations in OTU richness, DNA amount, and community composition in the community facets model

In addition to negative OTU-level responses, inoculations had dominantly negative effects on the resident community facets. Inoculations of *Antrodia piceata, Perenniporia subacida,* and *Skeletocutis stellae* decreased the OTU richness and DNA amount, and *Skeletocutis odora* had a negative effect on the DNA amount (Supporting Information: Table S7). Inoculations of *Antrodiella citrinella* had a positive effect on OTU richness, while inoculations of *Fomitopsis rosea* had a positive richness effect in 2020 but a negative richness and a positive DNA amount effect in 2021 (Supporting Information: Table S7). Also, inoculations of *Postia guttulata* increased OTU richness in 2020 but decreased the richness of rare OTUs after two years (Supporting Information: Table S7). Community composition changed as a response to inoculations of most target species (Supporting Information: Table S7).

Table S8. Proportion of OTUs assigned to different taxa at phylum and class level with statistically supported responses (posterior probability ≥ 0.95) to the inoculations of target species in the presence-absence model. Proportions are shown separately for each inoculated target species, for positive (+) and negative (–) responses, and for years 2020 and 2021 corresponding to one and two years after the inoculation treatment, respectively. Number of OTUs assigned to each taxon is showed after the name of the taxon. Taxa including a considerable proportion of statistically supported positive and negative responses are highlighted with red and blue: the darkest shade shows taxa with a proportion of responsive OTUs ≥ 0.75, medium shade with ≥ 0.50, and the lightest shade with ≥ 0.25.

			**Taxon**																
**Inoculated species**	**Response type**	**Year**	Phylum: *Ascomycota* (n = 141)	Class: *Dothideomycetes* (n = 24)	Class: *Eurotiomycetes* (n = 22)	Class: *Lecanoromycetes* (n = 10)	Class: *Leotiomycetes* (n = 35)	Class: *Saccharomycetes* (n = 17)	Class: *Sordariomycetes* (n = 16)	Phylum: *Basidiomycota* (n = 78)	Class: *Agaricomycetes* (n = 16)	Class: *Agaricostilbomycetes* (n = 4)	Class: *Cystobasidiomycetes* (n = 2)	Class: *Dacrymycetes* (n = 6)	Class: *Exobasidiomycetes* (n = 5)	Class: *Microbotryomycetes* (n = 17)	Class: *Pucciniomycetes* (n = 1)	Class: *Tremellomycetes* (n = 20)	Phylum: *Zygomycota* (n = 4)
Antrodia piceata	+	*2020*	0	0	0	0	0	0	0	0	0	0	0	0	0	0	0	0	0
		2021	0	0	0	0	0	0	0	0	0	0	0	0	0	0	0	0	0
	–	*2020*	0.50	0.38	0.50	1	0.54	0.65	0.38	0.06	0.13	0	0	0	0	0.06	0	0.10	0
		2021	0.16	0.33	0.59	0	0.03	0	0.06	0.14	0.44	0.25	0	0	0	0	0	0.05	0.25
Antrodiella citrinella	+	*2020*	0.08	0	0.18	0	0.17	0	0	0.03	0.06	0	0	0	0	0.06	0	0	0
		2021	0.06	0	0.09	0	0.11	0	0	0.10	0.13	0	0	0	0.80	0.06	1	0	0
	–	*2020*	0.14	0.04	0	0	0	0.88	0.06	0.09	0.13	0.25	0	0	0	0.12	0	0.10	0
		2021	0.09	0	0	0	0	0.53	0.06	0.04	0.06	0	0	0	0	0.06	0	0.05	0
Fomitopsis rosea	+	*2020*	0.20	0.04	0.41	0	0.29	0.06	0.13	0.23	0	0	0	0	0.60	0.59	0	0.15	0.25
		2021	0.06	0	0.05	0	0.06	0.06	0	0.08	0	0	0	0	0	0.18	0	0.15	0
	–	*2020*	0	0	0	0	0	0	0	0	0	0	0	0	0	0	0	0	0
		2021	0	0	0	0	0	0	0	0	0	0	0	0	0	0	0	0	0
Perenniporia subacida	+	*2020*	0.01	0	0	0	0	0.06	0	0	0	0	0	0	0	0	0	0	0
		2021	0	0	0	0	0	0	0	0	0	0	0	0	0	0	0	0	0
	–	*2020*	0.08	0	0.05	0	0.11	0.06	0.25	0.10	0.31	0	0	0.17	0	0	0	0.10	0
		2021	0.31	0.17	0.05	0.20	0.49	0.53	0.50	0.12	0.19	0	0	0	0	0.24	0	0.05	0.50
Physisporinus crocatus	+	*2020*	0.01	0	0	0	0.03	0	0.06	0.03	0	0	0	0	0	0	0	0.10	0
		2021	0.02	0.04	0	0	0.03	0	0.06	0	0	0	0	0	0	0	0	0	0
	–	*2020*	0.05	0.04	0	0	0	0.18	0.06	0.09	0.06	0	0.50	0	0.40	0.06	0	0.05	0
		2021	0	0	0	0	0	0	0	0.09	0.13	0	0	0	0	0	0	0.25	0
Postia guttulata	+	*2020*	0	0	0	0	0	0	0	0	0	0	0	0	0	0	0	0	0
		2021	0	0	0	0	0	0	0	0	0	0	0	0	0	0	0	0	0
	–	*2020*	0.03	0.17	0	0	0	0	0	0.06	0.31	0	0	0	0	0	0	0	0
		2021	0.43	0.71	0.77	0.80	0.23	0.06	0.31	0.19	0.19	0.25	0.50	0	0	0.12	0	0.30	0.25
Skeletocutis odora	+	*2020*	0.01	0	0	0	0	0.06	0.06	0.05	0.06	0	0	0	0	0.06	0	0.10	0
		2021	0.01	0	0	0	0	0.06	0.06	0.03	0	0	0	0	0	0.06	0	0.05	0
	–	*2020*	0.04	0	0	0.10	0.11	0.06	0	0.04	0.06	0	0	0	0	0	0	0	0
		2021	0.07	0	0	0.10	0.09	0.18	0.06	0.08	0.25	0	0	0	0	0	0	0	0
Skeletocutis stellae	+	*2020*	0.01	0	0	0	0	0	0.06	0	0	0	0	0	0	0	0	0	0
		2021	0.01	0	0	0	0	0	0.06	0	0	0	0	0	0	0	0	0	0
	–	*2020*	0.15	0.25	0.23	0.40	0.03	0.06	0	0.01	0	0	0	0	0	0	0	0.05	0
		2021	0.28	0.17	0.64	0.60	0.23	0	0.13	0.49	0.31	0.25	0	0.17	0.20	0.35	0	0.95	0
Steccherinum collabens	+	*2020*	0.01	0	0	0	0.03	0	0	0.01	0	0	0	0	0	0.06	0	0	0
		2021	0	0	0	0	0	0	0	0.01	0	0	0	0	0	0.06	0	0	0
	–	*2020*	0	0	0	0	0	0	0	0	0	0	0	0	0	0	0	0	0
		2021	0	0	0	0	0	0	0	0	0	0	0	0	0	0	0	0	0

References

Abarenkov, K. (2022). sh_matching_data_0_5_v3.zip. Supporting files for EOSC-Nordic service (SH matching analysis v2.0.0). URL: https://app.plutof.ut.ee/filerepository/view/5582954

Abarenkov, K., Somervuo, P., Nilsson, R.H., Kirk, P.M., Huotari, T., Abrego, N., *et al.* (2018). Protax-fungi: a web-based tool for probabilistic taxonomic placement of fungal internal transcribed spacer sequences. *New Phytol*., 220, 517–525.

Ahti, T., Hämet-Ahti, L. & Jalas, J. (1968). Vegetation zones and their sections in northwestern Europe. *Ann. Bot. Fenn*., 5, 169–211.

Callahan, B.J. (2020). DADA2 ITS Pipeline Workflow (1.8) [WWW Document]. URL: https://benjjneb.github.io/dada2/ITS_workflow.html (accessed 18 Sep 2020).

Callahan, B.J., McMurdie, P.J., Rosen, M.J., Han, A.W., Johnson, A.J.A. & Holmes, S.P. (2016). DADA2: High-resolution sample inference from Illumina amplicon data. *Nat. Methods,* 13, 581–583.

Edgar, R.C. (2010). Search and clustering orders of magnitude faster than BLAST. *Bioinformatics,* 26, 2460–2461. doi:10.1093/bioinformatics/btq461

Gardes, M. & Bruns, T.D. (1993). ITS primers with enhanced specificity for basidiomycetes – application to the identification of mycorrhizae and rusts. *Mol. Ecol.,* 2, 113–118.

Kotiranta, H., Junninen, K., Halme, P., Kytövuori, I., von Bonsdorff, T., Niskanen, T., *et al*. (2019). Aphyllophoroid fungi. In: *The 2019 Red List of Finnish Species* (eds. Hyvärinen, E., Juslén, A., Kemppainen, E., Uddström, A. & Liukko, U.-M.). Ministry of the Environment & Finnish Environment Institute. Helsinki, pp. 234–247.

Landau, W.M. (2021). The targets R package: a dynamic Make-like function-oriented pipeline toolkit for reproducibility and high-performance computing. *J. of Open Source Softw.,* 6, 2959. doi: 10.21105/joss.02959

Martin, M. (2011). Cutadapt removes adapter sequences from high-throughput sequencing reads. *EMBnet j.,* 17(1), 10–12. doi:10.14806/ej.17.1.200

Natural Resources Institute Finland (2019). Monilähteisen valtakunnan metsien inventoinnin (MVMI) kartta-aineisto 2019. https://kartta.luke.fi/index.html (accessed 19 Apr 2021).

Niemelä, T. (2016). *Suomen käävät*. Finnish Museum of Natural History LUOMUS. Helsinki. Finland.

Ovaskainen, O., Abrego, N., Somervuo, P., Palorinne, I., Hardwick, B., Pitkänen, J.M., *et al.* (2020). Monitoring Fungal Communities With the Global Spore Sampling Project. *Front. Ecol. Evol.,* 7, 1–9.

R Core Team. (2022). R: A language and environment for statistical computing. R Foundation for Statistical Computing. Vienna, Austria. https://www.R-project.org/

Renvall, P. (1995). Community structure and dynamics of wood-rotting Basidiomycetes on decomposing conifer trunks in northern Finland. *Karstenia,* 35, 1–51.

Rognes, T., Flouri T., Nichols, B., Quince, C, & Mahé, F. (2016). VSEARCH: a versatile open source tool for metagenomics. *PeerJ* 4, e2584.

Somervuo, P., Yu, D.W., Xu, C.C.Y., Ji, Y., Hultman, J., Wirta, H., *et al.* (2017). Quantifying uncertainty of taxonomic placement in DNA barcoding and metabarcoding. *Methods Ecol. Evol*., 8, 398–407.

Vu, D., Nilsson, R.H. & Verkley, G.J.M. (2022). Dnabarcoder: An open-source software package for analysing and predicting DNA sequence similarity cutoffs for fungal sequence identification. *Mol. Ecol. Resour.* 22. doi:10.1111/1755-0998.13651.

White, T.J., Bruns, T.D., Lee, S.B. & Taylor, J.W. (1990). Amplification and direct sequencing of fungal ribosomal RNA genes for phylogenetics. In: *PCR protocols: a guide to methods and applications* (eds. Innis, M.A., Gelfand, D.H., Sninsky, J.J. & White, T.J.). Academic Press. New York, pp. 315–322.
